# Protein-Protein Interactions in a Crowded Environment: An Analysis via Cross-Docking Simulations and Evolutionary Information

**DOI:** 10.1371/journal.pcbi.1003369

**Published:** 2013-12-05

**Authors:** Anne Lopes, Sophie Sacquin-Mora, Viktoriya Dimitrova, Elodie Laine, Yann Ponty, Alessandra Carbone

**Affiliations:** 1 Université Pierre et Marie Curie, UMR 7238, Equipe de Génomique Analytique, Paris, France; 2 CNRS, UMR 7238, Laboratoire de Génomique des Microorganismes, Paris, France; 3 Laboratoire de Biochimie Théorique, CNRS UPR 9080, Institut de Biologie Physico-Chimique, Paris, France; 4 LIX, CNRS UMR 7161 - INRIA AMIB, École polytechnique, Palaiseau, France; University of Maryland Baltimore County, United States of America

## Abstract

Large-scale analyses of protein-protein interactions based on coarse-grain molecular docking simulations and binding site predictions resulting from evolutionary sequence analysis, are possible and realizable on hundreds of proteins with variate structures and interfaces. We demonstrated this on the 168 proteins of the Mintseris Benchmark 2.0. On the one hand, we evaluated the quality of the interaction signal and the contribution of docking information compared to evolutionary information showing that the combination of the two improves partner identification. On the other hand, since protein interactions usually occur in crowded environments with several competing partners, we realized a thorough analysis of the interactions of proteins with true partners but also with non-partners to evaluate whether proteins in the environment, competing with the true partner, affect its identification. We found three populations of proteins: strongly competing, never competing, and interacting with different levels of strength. Populations and levels of strength are numerically characterized and provide a signature for the behavior of a protein in the crowded environment. We showed that partner identification, to some extent, does not depend on the competing partners present in the environment, that certain biochemical classes of proteins are intrinsically easier to analyze than others, and that small proteins are not more promiscuous than large ones. Our approach brings to light that the knowledge of the binding site can be used to reduce the high computational cost of docking simulations with no consequence in the quality of the results, demonstrating the possibility to apply coarse-grain docking to datasets made of thousands of proteins. Comparison with all available large-scale analyses aimed to partner predictions is realized. We release the complete decoys set issued by coarse-grain docking simulations of both true and false interacting partners, and their evolutionary sequence analysis leading to binding site predictions. Download site: http://www.lgm.upmc.fr/CCDMintseris/

## Author Summary

Protein-protein interactions (PPI) are at the heart of the molecular processes governing life and constitute an increasingly important target for drug design. Given their importance, it is vital to determine which protein interactions have functional relevance and to characterize the protein competition inherent to crowded environments, as the cytoplasm or the cellular organelles. We show that combining coarse-grain molecular cross-docking simulations and binding site predictions based on evolutionary sequence analysis is a viable route to identify true interacting partners for hundreds of proteins with a variate set of protein structures and interfaces. Also, we realize a large-scale analysis of protein binding promiscuity and provide a numerical characterization of partner competition and level of interaction strength for about 28000 false-partner interactions. Finally, we demonstrate that binding site prediction is useful to discriminate native partners, but also to scale up the approach to thousands of protein interactions. This study is based on the large computational effort made by thousands of internautes helping World Community Grid over a period of 7 months. The complete dataset issued by the computation and the analysis is released to the scientific community.

## Introduction

Protein-protein interactions (PPI) are at the heart of the molecular processes governing life and constitute an increasingly important target for drug design [1]–[4]. Given their importance, it is clearly vital to characterize PPIs and notably to determine which protein interactions are likely to be stable enough to have functional relevance. Computational methods such as molecular docking have rendered possible to successfully predict the conformation of protein-protein complexes when no major conformational rearrangement occurs during the assembly [5]–[11]. However, we [12] and others [13], [14] have demonstrated that docking algorithms are unable to predict binding affinities and thus, presently, cannot distinguish which proteins will actually interact. This leads to ask whether this failure comes from the fact that scoring functions, used to sort the docking solutions, are inefficient for partner identification or whether the difficulty comes from binding promiscuity between proteins in the cell that blurs the interaction signal of the functional partners. In the crowded cell, proteins experience non-specific and unintended interactions with the intracellular environment leading to a severe competition between functional and non-functional partners [15]–[19]. This brings to light the importance of characterizing weak, potentially non-functional, interactions in order to predict functional ones and understand how proteins behave within a crowded environment [16], [20], [21].

In this work, we tackle two distinct but related questions: (i) can a combination of coarse-grain docking and evolutionary information identify true interacting partners among a set of potential ones? (ii) what is the effect of binding promiscuity on a large and variate dataset of protein structures [22]?

Previously, we have shown that knowing the experimental binding site of a protein can help to retrieve its native interacting partner within a set of decoys [12]. On the other hand, recent studies reveal that arbitrary docked partners bind in a non-random mode on protein surfaces [23], [24] suggesting that docking true but also false partners can help to identify protein binding sites. We developed a novel score based on arbitrary docking and evolutionary information to predict protein binding sites. The different docking conformations of a given protein pair are scored according to their associated energy and the agreement between the docked interface and the predicted binding sites. An interaction index is defined, and normalized according to the whole set of proteins tested, in order to discriminate the interacting partners from the set of tested interactions.

We evaluate our method with a complete cross-docking (CC-D) calculation on a set of 168 proteins belonging to the 84 known complexes described in the Mintseris Benchmark 2.0 [25] and covering a large spectrum of different protein interfaces. Enzymes, inhibitors, antibodies, antigens, signaling proteins and others have been considered as well as interfaces that do or do not undergo conformational adjustments during interaction. Docking calculations are made with no knowledge of the experimental complex structure: unbound structures are used. We use a coarse-grain docking algorithm [12], whose energy function relies on both van der Waals and Coulomb potentials. We show that the combination of a coarse-grain docking algorithm with binding sites prediction can significantly contribute to the identification of a reasonably sized set of potentially interacting proteins, that can be further investigated by more precise docking algorithms or laboratory experiments.

The large computational effort necessary to accomplish this work was realized with the help of World Community Grid (WCG), that coordinated thousands of internautes providing their computer time to dock about 300000 conformations per protein pair for the set of 28224 possible pairs in the Mintseris Benchmark 2.0. For each pair, we selected about 2000 decoys. For non-partners, we find weak as well as strong interactions. The decoy set is released and it provides an important reference set of structures that can serve as a proxy for the non-specific protein-protein complexes that occur transiently in the cell or that are avoided by spatial-temporal constraints. These latter are hard to characterize experimentally but they are of biochemical relevance, as highlighted by other studies [26]–[29].

To simulate the variability of crowded environments for a protein in the cell, we study how easily a protein finds its true partner with respect to many random subsets of proteins supposedly competing with it. We realize a thorough analysis of these interactions and we address the question of whether a successful prediction of a protein partner depends on the environment composition or not. We quantify the effect of competing partners in predictions, and we characterize in a quantitative manner three distinguished populations of proteins interacting with a protein 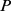: those that strongly compete with the true partner of 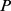, those that never compete with it, and those that interact with 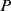 with variable levels of strength. For each protein 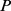, we propose a numerical index that provides the strength of the interaction with all other proteins in the environment, and that gives a signature for 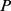.

To our knowledge, this is the first study performing a large-scale CC-D calculation, proposing an analysis of the binding promiscuity of the protein set, and providing to the scientific community the associated dataset of decoys [30], [31] at the same time. Previous large-scale analyses used docking by shape complementarity that quickly scans through several thousands proteins in a matter of seconds [32], [33] but ignore the electrostatic contribution playing however an important role in protein interactions [34]–[37]. We compared our method to two previously done studies [32], [33]. Both of them do not perform a CC-D experiment, but a large-scale analysis of selected protein pairs.

Finally, we checked whether evolutionary information can be used to considerably restrict the number of docking interfaces to be examined and to render molecular computation feasible for a larger scale investigation of PPIs, based on thousands of proteins instead of hundreds. This result makes the protocol proposed here feasible for scaling up the analysis.

## Results

The 168 proteins of the Mintseris Benchmark 2.0 [25] form 84 binary complexes known to interact in the cell. They cover three broad biochemical categories and three difficulty categories related to the degree of conformational change at the protein-protein interface. They are classified as Enzyme-Inhibitors (46 proteins), Antibody-Antigen (20), Antigen-Bound Antibody (24), Others (78), and also as Rigid Body (126), Medium (26), Difficult (16). The set is constituted by 51 multimeric proteins and by 117 monomeric ones forming 41 complexes where at least one of the protein is multimeric.

CC-D was realized on the full dataset from unbound structures, leading to 28224 docking simulations. Each calculation explored about 300000 ligand-receptor orientations, corresponding to ligand and receptor complete surfaces, and asked for more than 7 months computational time on WCG. This CC-D scaled up the one introduced in [12], carried out on 6 enzyme-inhibitor complexes.

The docking algorithm simulates the actual docking process in which ligand-receptor pairwise interaction energies are calculated. The energy function we used takes into account van der Waals (modeled by a Lennard-Jones potential) and electrostatic (modeled by a Coulomb potential) terms (see Methods).

### Predictions of protein partners

For each protein in the dataset, the problem of partner identification is tackled with two main experiments. The first experiment assumes to know the residues belonging to the experimental interface of the proteins. This means that the residues lying at the interface of two proteins in a native complex are supposed to be known while no knowledge of the complex conformation is assumed. The second experiment replaces experimental interfaces by predictions of binding sites based on docking and evolutionary information. The evaluation of the quality of the interaction signal in this PPI large-scale study is of major importance. In particular, the contribution of docking information compared to evolutionary information in partner identification needs to be quantified. To do so, the analysis based on experimental interfaces allows us to evaluate in a precise manner how much a good prediction of the interaction sites improves partners identification, experimental interfaces playing the role of perfect predictions. In the sequel, we also use it to decipher whether a property of protein interactions that has been observed from computational predictions has a biological origin or whether it is a consequence of the noise of the prediction.

#### Knowing experimental interfaces

As pointed out in [12], the combination of the energy score produced by docking and the knowledge of the experimental interface should help to retrieve the true interacting partners. For this, we define a predictive PPI index (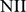) in order to estimate the probability of two proteins to interact. As in [12], we determine what fraction of the docking interface is composed of residues belonging to the experimentally identified interface (named FIR, for Fraction of Interface Residues) for the receptor (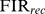) and for the ligand (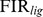), and we define the overall fraction of the complex as 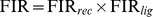. Then, we describe each receptor-ligand orientation by the product of its corresponding FIR and energy. Here, contrary to [12], for each pair of proteins 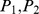, we compute an interaction index (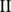)
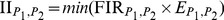
(1)where the minimum is defined over all orientations tested for 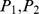, and the interaction energy 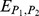 of the corresponding conformation.

To compare interaction indexes computed over different pairs, a normalized interaction index, called 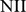, is introduced. In [12], a NII formula is also proposed but it uses a different definition (1) and it does not model the symmetric role played by ligand and receptor (see Methods).

**Figure 1:**
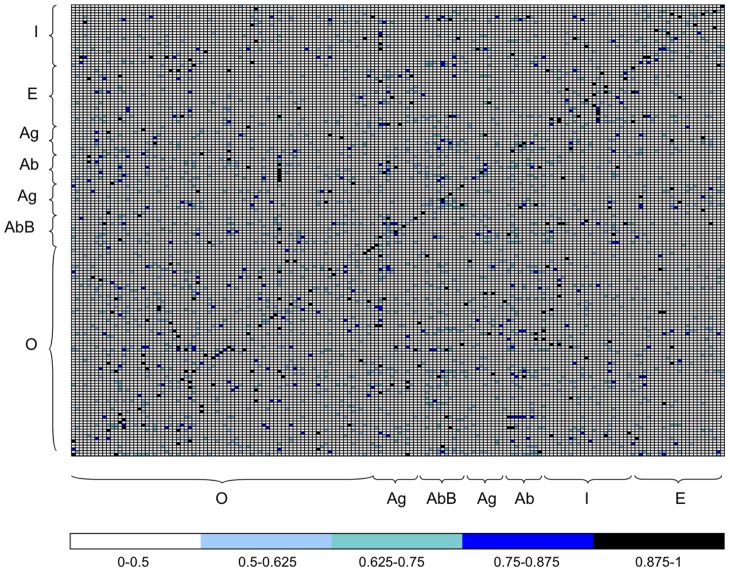
Normalized Interaction Index (NII) matrix for the complete dataset of 168 proteins. The matrix is ordered with the experimental complexes lying on the trailing diagonal. Protein structures corresponding to columns and rows are grouped in functional classes: Enzymes (E), Inhibitors (I), Antibody (Ab), Antigen (Ag), Bound Antibody (AbB), Others (O). Each entry of the matrix corresponds to the NII value computed for the corresponding pair of proteins (receptor on the y-axis and ligand on the x-axis). High interaction scores (between 0.7 and 1, blue and black in the color scale) indicate a high interaction probability. Interaction scores are computed using knowledge of the experimental interfaces. The plot corresponds to an 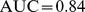. In the color bar the intervals correspond to 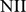 values, where the upper bound is included in each interval. Rows and columns are labeled with protein names in Figure S1 in Text S1.

The results of the analysis are resumed in the squared matrix reporting the 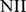 values of each pair of proteins in Figure 1, where one clearly distinguishes the diagonal that indicates a successful prediction for many native complexes (see the third column of Table 1 also, and Figure S1 in Text S1). The performance of the prediction has been evaluated using a receiver operating characteristic (ROC) curve and its area under the curve (AUC) is 0.84. At a NII score threshold of 0.5, one observes a very high specificity (92) and a good sensitivity (52). See Table 2 and Table S8 in Text S1 (for other thresholds and performance measures). The large spectrum of interfaces, the large number of partners in competition, and the usage of unbound structures (compared to the bound ones used in [6], [12]) render this successful result not a forgone conclusion. At the contrary, the results provide a very encouraging insight and confirm search for protein partners by docking simulations, starting from unbound structures, to be a feasible task.

**Table 1:** Interaction ranks distribution for the Mintseris Benchmark 2.0.

	Mintseris Benchmark 2.0 - 168 proteins				
Top %	# top proteins	168 vs 168 (%)		56 vs 168 (%)	
		exp	pred	exp	pred
1	1	42 (25)	6 (4)	16 (29)	3 (5)
5	8	76 (45)	23 (14)	28 (50)	9 (16)
10	17	98 (58)	41 (25)	36 (64)	17 (31)
15	25	118 (70)	50 (30)	45 (80)	20 (36)
20	34	126 (75)	59 (36)	45 (80)	23 (42)
30	50	136 (81)	76 (46)	47 (84)	33 (60)
40	67	145 (86)	98 (59)	49 (88)	38 (69)
50	84	154 (92)	117 (70)	54 (94)	41 (75)

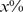
 of complexes obtained by docking the proteins with all 168 proteins in the environment (this means that the NII score of the native complex falls in the top 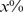 scores). Native complexes identification is realized either by knowing the experimental interface (exp) or by predicting it (pred). Cumulative counts and percentages are displayed. The selected set of 56 monomers considered in [33] is also evaluated against the 168 proteins (fifth and sixth columns). The number of top proteins corresponding to the 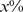 of the total number of proteins in the specified environment is given (second column). Over the 168 proteins in the Mintseris dataset, we report the number of proteins (third and fourth columns) whose native complex is identified within the top

**Table 2:** Partner prediction based on the exploration of the full conformational space.

Protein dataset		Experimental interfaces			Predicted interfaces				
Subset type	# proteins				JET+NIP			NIP	JET
		AUC	*Sen*	*Spe*	AUC	*Sen*	*Spe*	AUC	AUC
Mintseris DB	168	0.84	52	92	0.61^*^	25^*^	89^*^	0.53^*^	0.59^*^
Enzyme-Inhibitor & Others	124	0.84	54	92	0.66^*^	34^*^	87^*^	0.56^*^	0.65^*^
Enzyme-Inhibitor	46	0.85	59	88	0.77	65	78	0.60	0.72
Antibody-Antigen	20	0.89	95	66	0.58	15	70	0.61	0.52
Antigen-Bound Antibody	24	0.91	79	80	0.56	38	74	0.63	0.53
Others	78	0.84	62	89	0.61^*^	25^*^	88^*^	0.52^*^	0.62^*^
Rigid	126	0.87	59	91	0.60^*^	29^*^	85^*^	0.53^*^	0.59^*^
Medium	26	0.85	73	81	0.68	58	80	0.53	0.67
Difficult	16	0.77	69	78	0.65	38	80	0.66	0.63
Monomeric (both partners)	86	0.87	66	89	0.63	36	85	0.55	0.63
Multimeric (at least one partner)	82	0.81	59	88	0.61^*^	32^*^	86^*^	0.51^*^	0.61^*^

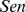
) and specificity (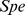) are also given at a NII threshold cutoff of 0.5 for predictions based on experimental interfaces, and at a NII threshold cutoff of 0.25 for predicted interfaces. Calculations based on JET and NIP predicted interfaces use weights 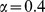, 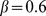 (see Methods). The * symbol refers to values computed on subsets that have been cleaned of the complex 1ML0 for which JET provided no interaction site (leading to a 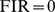 because of no common residue between the small predicted interface and the docked one). The Mintseris dataset and the subsets Enzyme-Inhibitor & Others, Others, Rigid and Multimeric contain 166, 122, 76, 124 and 80 proteins, respectively. See also Tables S8–S9 in Text S1 for other threshold cutoffs and performance measures. The analysis is realized by assuming knowledge of either the experimental interfaces or the predicted interfaces. Performance of partner prediction is evaluated through AUC values computed on the Mintseris dataset and its different subsets. Sensitivity (

#### Analysis of different classes of interaction based on experimental interfaces

**Figure 2:**
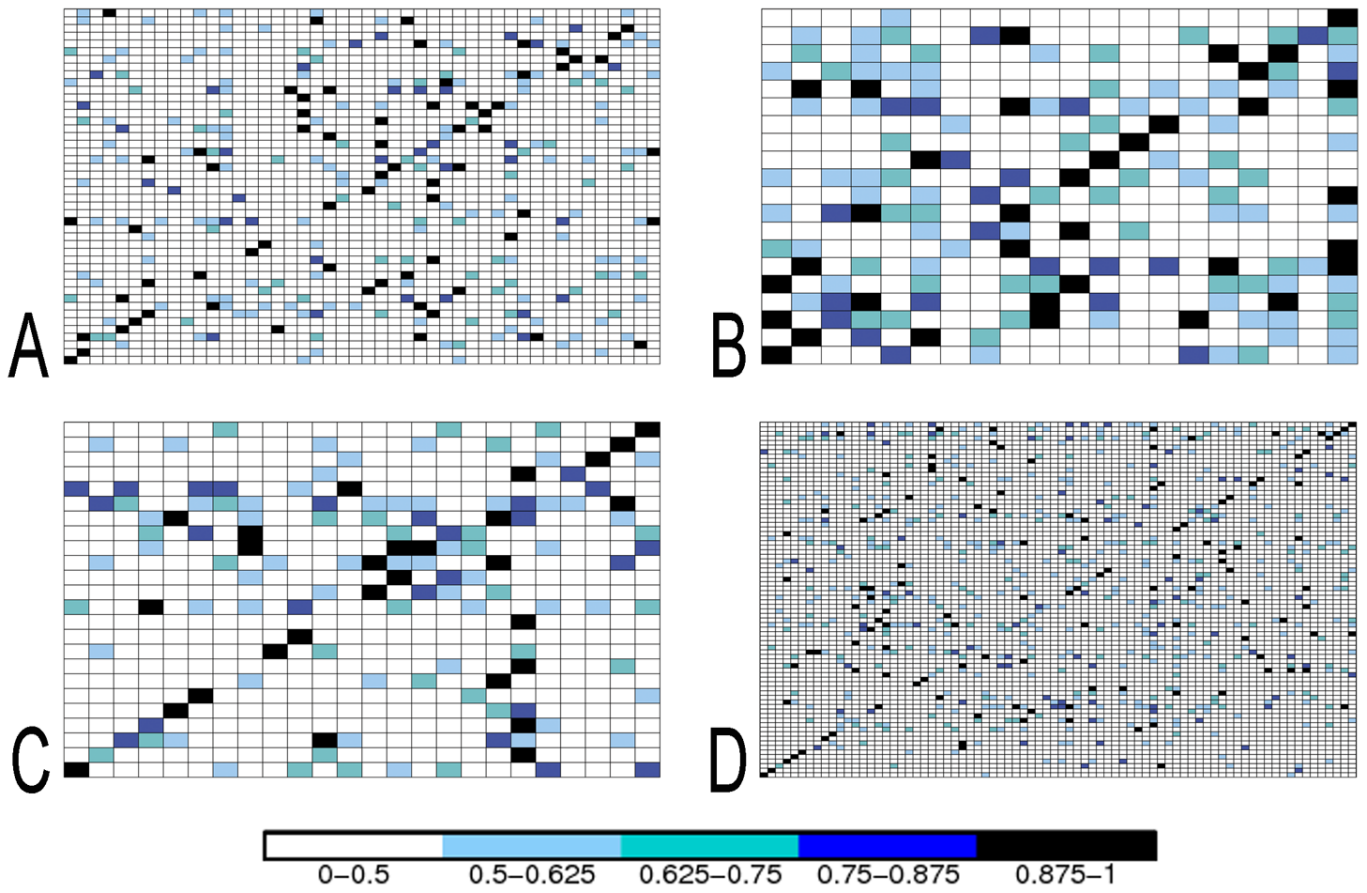
NII matrices for functional classes of proteins. Enzyme-Inhibitors (EI; top left), Antibody-Antigen (AbAg; top right), Antigen-Bound Antibody (AgAbB; bottom left), Others (O; bottom right). See legend of Figure 1 for matrix description and color scale. Protein structures are grouped in functional classes. (See Figure S2 in Text S1 for the version of the figure reporting protein names on matrices columns and rows.)

We systematically analyzed complexes in terms of their biochemical classes and difficulty categories (see Figure 2, Table 2 and Table S8 in Text S1) to verify whether partner identification, based on experimental interfaces, is easier within certain classes than within others. Partners prediction improves to an 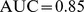 for Enzyme-Inhibitors, 0.89 for Antibody-Antigen, 0.91 for Antigen-Bound Antibody, 0.84 for Others (Figure S2 in Text S1). Similarly, we obtain an 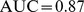 for Rigid Body, 0.85 for Medium and 0.77 for Difficult structures (Figures S3–S4 in Text S1). Therefore, when the binding sites are known, interactions within classes are clearly easier to predict. To understand these results, it is important to observe that Enzyme-Enzyme, Antigen-Antigen and Bound Antibody-Bound Antibody interactions are well discriminated by docking. This is highlighted by a large amount of extremely weak interactions, if any at all, detected within these sub-classes and illustrated by the corresponding sub-matrices in Figure 2. In conclusion, the good behavior of partner prediction within functional classes might be due either to the size effect of the environment on the prediction or to the composition of the protein subset used for predicting. This question is explored in “Native interactions and competing partners”.

#### Using predictions of interaction sites

Here, we drop the information on the location of the experimental interface of true partners and use predicted binding sites instead in order to evaluate our ability to retrieve the true partner in a totally blind experiment. Predicted interfaces are obtained by combining evolutionary information, computed with the program JET [38] (see Methods) and CC-D calculations:

- JET predictions are used to select a set of residues that are likely to belong to the real interface according to conservation and expected physico-chemical properties for interface residues (see Methods) [38];
- Early studies suggested that docking arbitrary partners together can nevertheless point to the correct interaction surfaces [23], [39]. For this, they observed an accumulation of the docking solutions around the experimental location of the true partner. Following this approach, given a protein 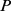, we used the information extracted from CC-D calculations involving all proteins in the database, to propose a set of residues that is highly likely to belong to the binding site of 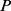. A score (called Normalized Interaction Propensity, or NIP, in Methods, Eq. (7)), associated to each residue in 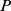, reveals the probability for a residue to belong to the real interface.

**Figure 3:**
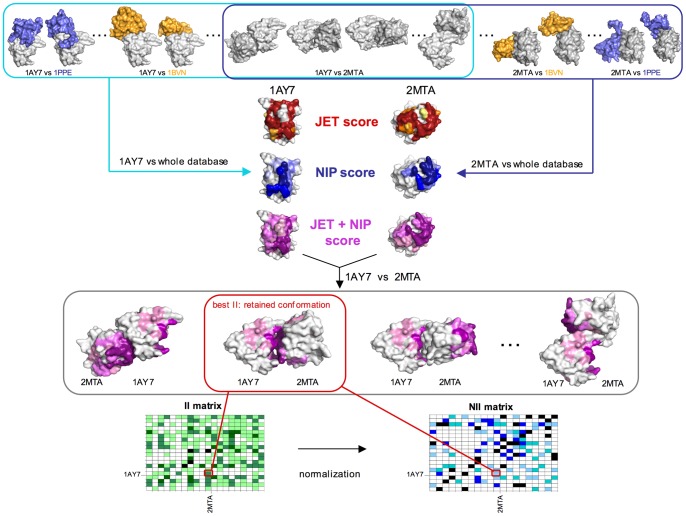
The protocol. The protocol is based on docking calculations and JET predictions and produces an interaction matrix for the proteins in a database. Here, two protein structures, the receptor 1AY7 and the ligand 2MTA, are analyzed. The first step consists in cross-docking 1AY7 and 2MTA respectively, against all structures in the database (see cyan box for 1AY7 and blue box for 2MTA). A structure will be crossed dock against another in several conformations (from 100000 up to 450000, depending on the size of the proteins). In the schema, 1AY7 and 2MTA are also docked one against the other (see intersection between blue and cyan boxes). As a result of the cross-docking, a NIP score is associated to each protein leading to the prediction of an interaction site (color range from light blue to dark blue, corresponding to weak and strong signals respectively). In parallel, each protein is analyzed with JET, a JET score is associated to it and leads to the prediction of an interaction site based on evolutionary information (color range from yellow to red, corresponding to weak and strong signals respectively). JET and NIP scores are finally combined to obtain a JET+NIP score for each protein structure (color range from light pink to deep purple, corresponding to weak and strong signals respectively). Then, for each docked conformation, the JET+NIP score is combined to the corresponding energy value (to compute the FIR) to discriminate the best conformation of 1AY7 and 2MTA among all possible conformations computed by cross-docking (grey box, corresponding to the intersection of cyan and blue boxes - notice that the orientation of 1AY7 is the same in all conformations represented in the box). For the full dataset, the FIR values of the best conformation computed for each pair of proteins are recorded in the 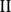 matrix. Notice that the schema describing the computation for 1AY7 and 2MTA leads to one entry of the matrix. Finally, a normalization step produces the NII matrix used to discriminate potential partners.

For a given pair of proteins 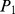 and 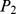, we evaluated all docking conformations by combining NIP residue scores and JET residue scores at the corresponding interface (this defines a 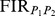 as indicated in Methods, Eq. (8)) with the energy of the conformation (see Figure 3). Intuitively, we select the conformation that shows highest NIP and JET scores at the interface together with a sufficiently low energy. The best conformation satisfies Eq. (1). As in section “Knowing experimental interfaces”, the resulting 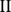 matrix is normalized into a 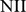 matrix (see Methods, Eq. (3)).

We analyzed partner prediction performances obtained by using NIP, JET or the combination JET+NIP (see Table 2 and Table S9 in Text S1; see also Figure S7–S8 and Table S10 in Text S1). When using only NIP, about half of the dataset (Enzyme-Inhibitors, Antibody-Antigens and Antigen-Bound Antibody) reaches an AUC of about 0.60, while the other half (Others) shows a random behavior. The use of JET highly improves the performance of three quarters of the dataset (Enzyme-Inhibitors and Others) with an AUC increase of more than 10% (for each class), while for the remaining quarter, involving antibodies and antigens, the AUC drops to 0.52.

To take advantage of the different behavior of NIP and JET on different biochemical classes, we combined the two approaches. We obtained a global improvement for three quarters of the database (Enzyme-Inhibitors and Others) compared to the results of NIP alone (0.77 and 0.61, respectively), while increasing the performance for Antibody-Antigens (0.58) and Antigen-Bound Antibody (0.56) compared to JET alone.

The poor performance obtained on Antibody-Antigens and Antigen-Bound Antibody possibly results from (i) a faster sequence evolution that blurs conservation signals leading to bad binding site predictions and (ii) from a large number of JET patches (corresponding to potential binding sites), compared to Enzyme-Inhibitors and Others, generating a large number of potential interactions that render more difficult partners discrimination. By excluding Antibody-Antigen and Antigen-Bound Antibody from the dataset (see “Enzyme-Inhibitors & Others” in Table 2), the AUC reaches 0.66 on the 124 remaining proteins. This is an encouraging outcome considering the absence of experimental information and the important number of competing proteins. Notice that on Enzyme-Inhibitors, the combination JET+NIP improves the already good JET performance to an AUC of 0.77. In particular, JET+NIP obtains a decrease of less than 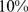 compared to predictions based on experimental data. See Tables 1, 2 and Table S9 in Text S1 (for several performance measures and score thresholds). Finally, very weak interactions among Enzyme-Enzyme and Bound Antibody-Bound Antibody proteins are observable, as already noticed for docking based on experimental interfaces. This is highlighted by the sub-matrices in Figure S8 in Text S1 and constitutes another encouraging outcome for exploring interacting networks with docking based on interface predictions.

#### Analysis on difficulty categories based on interface predictions

When using JET+NIP scores, the partitioning of the Mintseris dataset on Rigid, Medium and Difficult structures leads to 0.60, 0.68 and 0.65 AUC values respectively (Table 1 and Table S9 in Text S1). Unexpectedly, Difficult and Medium perform similarly, and better than Rigid. This suggests that the interface prediction based on JET+NIP is robust to conformational changes that could occur upon complex formation, probably due to the fact that 1. JET is based on sequence information, and 2. JET is designed to predict surface residues that are possibly highly buried [38], and that can pass from a buried to an exposed state (and conversely) during the assembly.

The Rigid class shows the worst performance, possibly due to the presence of Antibody-Antigen and Antigen-Bound Antibody in this subset. The comparison of Rigid with the Enzyme-Inhibitors & Others subset, displaying a similar size but an AUC of 0.66, shows that the AUC is independent of the size of the evaluated subset and suggests that the subset composition might play an important role in the prediction reflecting the partners competition occurring in the cell. This leads to ask whether the prediction of a given complex is dependent on the proteins composing the environment or whether it is dependent on intrinsic properties of the complex itself. This idea is explored in the next section.

### Native interactions and competing partners

We performed a series of tests to check whether the composition of a set of competing partners for a given protein influences partnership prediction. The analysis is performed on both JET+NIP predictions and experimental interfaces (see Figures S10–S16, S17–S23 and Table S1 in Text S1).

#### Are predictions dependent on environment composition?

**Figure 4:**
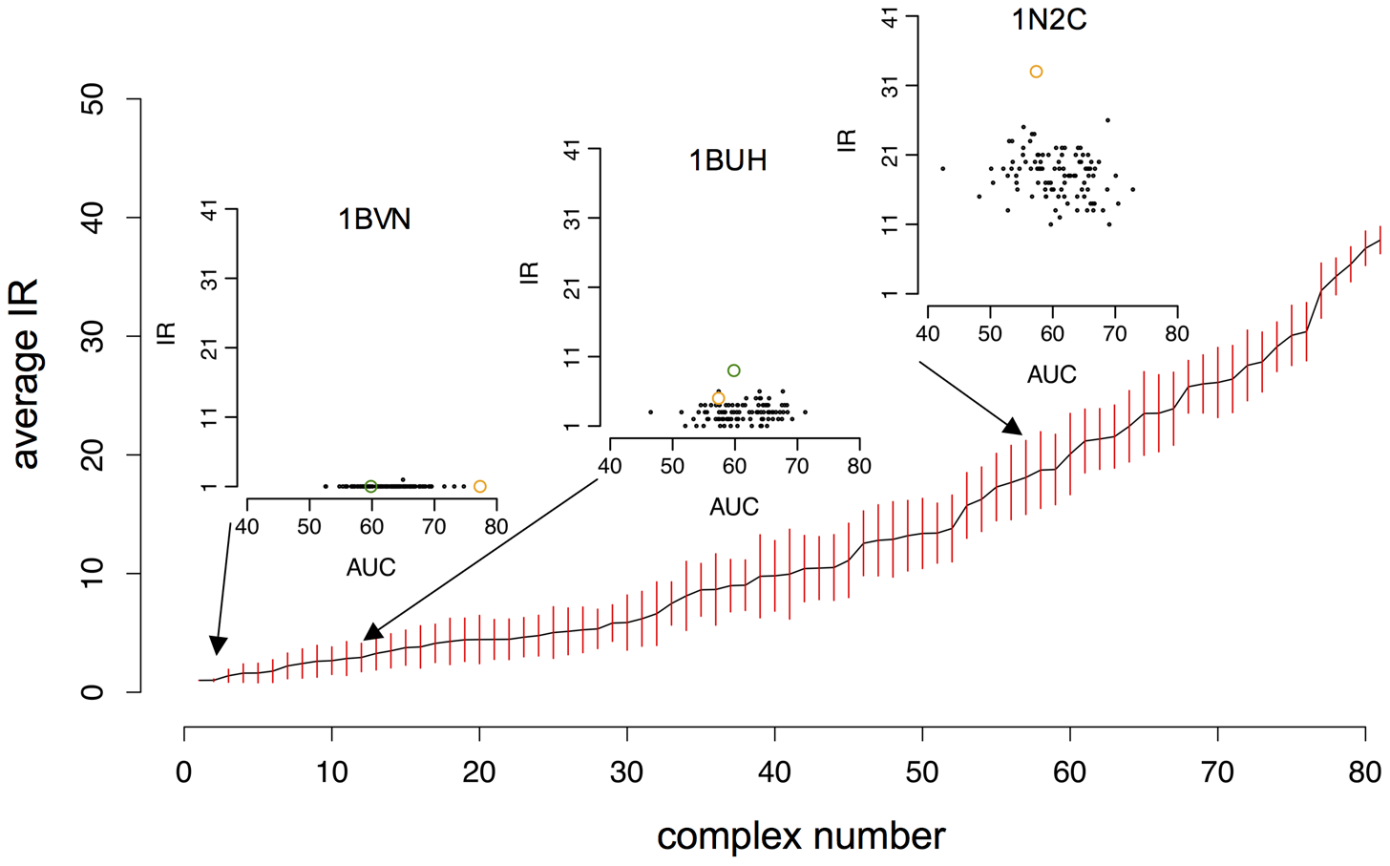
Robustness of the native complex predictions with respect to the environment composition. Partner predictions are based on predicted interfaces. Average Interaction Rank (IR) of the true partners is computed over 100 random sets made of 40 proteins each (with error bars in red). The 84 complexes are ordered with respect to their increasing average IR value. For three of the 84 complexes (1BVN, 1BUH, 1N2C), detailed plots show the IR of the complex within each of the 100 random sets and the corresponding AUC value (black dots); green dots correspond to the IR of the complex computed over the Mintseris dataset; orange dots correspond to the IR of the complex computed over complexes in the same functional class. Note that the absence of the green dot on the 1N2C plot corresponds to a too large IR (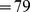) of the complex. See Table S2 in Text S1 for the names of complexes ranked on the 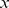-axis. See Figure S55 in Text S1 for robustness of predictions based on experimental interfaces.

We investigated the robustness of the prediction of a given complex among different random sets of proteins, these sets containing potential competitors for the proteins forming the complex. To do so, we defined the interaction rank (IR) of a complex within an environment to be the position of the NII value of the complex in the ordered list of NII values associated to the complexes involving at least one of the two partners. For each of the 84 native complexes, we generated 100 protein sets containing the desired complex and 19 other randomly chosen complexes (40 proteins). Figure 4 illustrates the average and standard deviation of the distribution of IRs for the native complexes, ordered by increasing average rank. We can see that about the half of the native complexes are predicted in the top 10 (41 complexes over the 84), and 62% (52 over 84) are predicted on the top 15. This finding strongly suggests that these well-behaved complexes display some intrinsic properties leading to the correct prediction whatever the associated random set of potential partners is.

In Figure 4, one can also observe an increase in the variability of the IR (see the size of red bars) for those complexes whose average IR is greater than 10, and a strong decrease for a few complexes having a very large average IR value, that is complexes that are hard to predict (see right of Figure 4). Receptor and ligand forming these latter complexes may interact with many proteins in the cell thus displaying some binding promiscuity. In particular, these bad-behaved complexes are proteases, kinases, cell adhesion molecules and MHC (major histocompatibility complex) class II molecules. The first two kinds of proteins are known to interact with many partners, while the last two are normally located on cell surfaces and display conserved interfaces, usually buried within the cell wall. On the contrary, these conserved interfaces are exposed in our experiments, possibly diverting the JET signal. Notice that the observation remains true when using experimental interfaces, showing that this behavior is not due to noise coming from the prediction (see Figure S55 in Text S1).

For a given complex, we also plot the detailed values of AUC and IR of the associated 100 random sets (see the three detailed plots in Figure 4 and Figures S17–S23 in Text S1 for the complete analysis of the 84 complexes). Intuitively, a high AUC value corresponds to a random set of complexes for which the large majority of the predictions is correct, while low AUC values (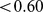) correspond to a majority of bad predictions. Two groups of proteins with distinct behavior emerge. The first one is constituted by complexes whose IR is independent on the random set composition. They display a small variability of the IR and a large variability of the corresponding AUC (see Figure 4, 1BVN). This group is constituted by 24 complexes over 84 and it corresponds to complexes possibly displaying intrinsic physico-chemical properties always leading to the same IR (average IR 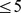), whatever the associated random set is. The second group corresponds to complexes displaying a correlation between IR and AUC values. It reveals that the composition of the different random sets might influence the ranking of the reference complex (see Figure 4, 1N2C) that might vary from set to set, thus mimicking, to some extent, the competition that occurs in the cell. With the exception of a few complexes, IRs range within at most 10 positions reflecting some stability of the complex ranks, whatever the subset is. Notice that when experimental interfaces are known, these two behaviors hold true, supporting the idea that they are not a consequence of a loss of interaction signal due to unsuccessful predictions. See Figures S10–S23 in Text S1.

Overall, many complexes display a very good average IR (Table S1 in Text S1). Among the 84 complexes, 41 show an average IR 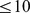 and 52 an average IR 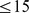. Here, the good performance of Enzyme-Inhibitors observed before, is confirmed with 11 over 23 complexes showing an average IR 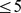, and 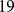 an average IR 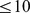. For Antibody-Antigen and Others, a bit more than a third of the complexes show an average IR 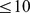, which is an encouraging result taking into account the absence of any experimental information. If we suppose to know experimental interfaces, there are 15 complexes with average IR 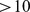, indicating a set of complexes for which coarse-grain docking does not provide sufficient information to discriminate partnership. These complexes mostly belong to Enzyme-Inhibitors and Others (see Figures S10–S16 in Text S1).

#### A protein signature based on the variability of its interaction ranks

The notion of average IR (computed over a large number of random sets) can be used to measure the strength of the interaction between two arbitrary proteins. Based on it, we ask whether complexes involving wrong partners might display the same average IR value and IR standard deviation of native complexes. Given a protein 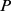, we run our previous test using each of the 168 proteins as a partner, and for each pair of (possibly false) partners we compute average IR and average AUC over 100 random sets of 40 proteins. By representing complexes with these pairs of values, we aim to analyze the whole set of complexes associated to 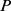. See Figure 5 for the analysis of receptor and ligand of complex 1BUH, where a few conformations formed either by the receptor or the ligand are reported. See Figures S24–S37 and S38–S51 in Text S1 for the analyses on the whole Mintseris dataset.

**Figure 5:**
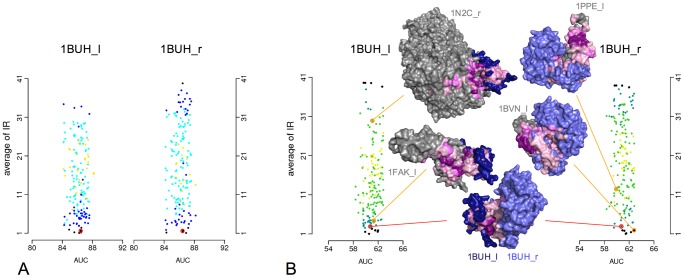
Robustness of the IR for the true partners and the false ones for the 1BUH complex. A. Each partner of the 1BUH complex is coupled with one of the 168 proteins (including the monomer itself) of the dataset forming either a false (167 cases) or the native complex. For each complex, we computed the corresponding average IR and average AUC over 100 random sets of 40 proteins, obtained by using the experimental interfaces and the full exploration of the conformational space. These values are reported as a point in a plot. Each plot contains 168 points. The red circle in each plot corresponds to the values of the native complex. Dots are colored in a scale from black, blue, cyan to yellow. A color corresponds to the value of the standard deviation 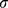 of the distribution of 100 IRs computed for a complex: black if 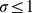, blue if 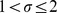, cyan if 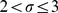 and yellow otherwise (i.e. 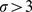). B. The analysis in A is realized here with 1BUH coupled with 166 proteins (here we have not considered the complex 1ML0 of the Mintseris Benchmark 2.0 because JET made no predictions and this turned out to provide no 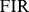 value), with predicted interfaces and the full exploration of the conformational space. Dots are colored in a scale from black, cyan, green to yellow. A color corresponds to the value of the standard deviation 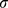 of the distribution of 100 IRs computed for a complex: black if 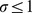, cyan if 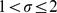, green if 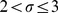 and yellow otherwise (i.e. 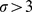). The structures of the native complex (red circle) and of four selected false complexes (orange circles) are shown to illustrate the conformations corresponding to the best II value. Notice that the II value is always the same for the 100 random runs while the NII varies with respect to the dataset of proteins used in a run. The receptor 1BUH_r is colored in light blue while the ligand 1BUH_l is colored in dark blue. The four other proteins are colored in grey. All residues with a JET+NIP score 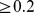 display interaction propensity and are colored in a color range going from light pink (weak signal) to deep purple (strong signal) for the 6 structures. See Figures S24–S37 and S38–S51 in Text S1 for the same analysis on all complexes in the Mintseris Benchmark 2.0.

As seen on the 1BUH complex, there is a strong variability of the average IR values associated to the interactions of a protein with different partners. One distinguishes three populations of proteins in the environment that interact with 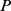:

1. those that rank always on the top positions: they strongly compete with the native complex by creating structures of very low energy score and displaying the interaction on the expected binding sites, these latter being either predicted or experimentally validated. They are represented by black dots and lie on the bottom of the plots in Figure 5B. Notice that they are not the same for different 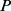
's and do not belong to a particular functional class (see Figures S56 and S57 in Text S1).
2. those that rank always as the last: they never enter in competition with the native complex, possibly due to their physico-chemical characteristics. They are represented by black dots and lie on the top of the plots in Figure 5B.
3. those that rank on the middle: they interact with different levels of strength with 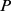. They form the larger group and they can be distinguished in subgroups with respect to the rank value and its stability. They are represented by cyan, green and yellow dots in Figure 5B (see legend). Green and yellow non-native complexes display important IR variations appearing to be dependent on subsets composition.

For each protein, partners belonging to these three populations and level of strength of their interactions measured by the average IR, are precisely computed and they form a signature for the protein interaction with its environment.

#### Average IR of true partners

In many cases (41 complexes over 84), true partners display an average IR 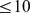, and on a predictive perspective, one of the major difficulties is to discriminate the true partners from the wrong ones displaying good average IR (black and cyan dots at the bottom of the plots in Figure 5B). Also, the stability of the average IR gradually decreases with the incrementation of the IR values until rank 20, and then gradually increases (see variation of the colors from black to yellow and from yellow to black in Figure 5B). This is a pattern observed for all proteins and reveals that extreme IR values are very stable. Based on this observation, some proteins could be eliminated from the list of potential partners with a very high confidence.

**Figure 6:**
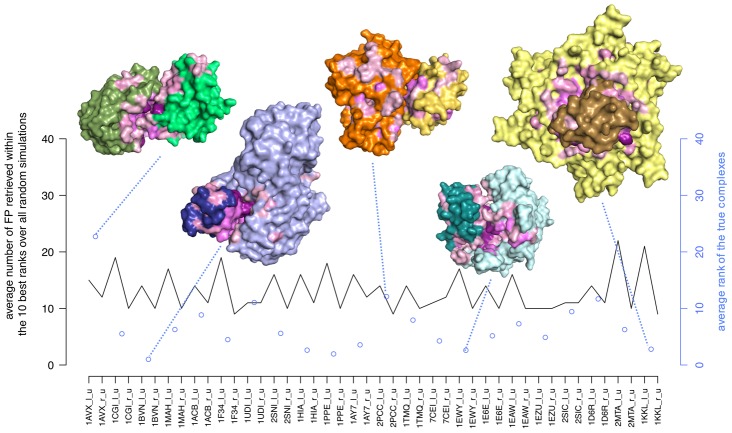
Average IR for true Enzyme-Inhibitor complexes and number of false positives. For each protein, we plot as false positives (FPs, black curve) the number of partners (excepted the true one) showing an average IR 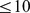, where the IR is computed over 100 random sets of 20 complexes selected from the set of 46 Enzyme-Inhibitor proteins. The cyan dots indicate the average IR of the true partner. A dot corresponds to a complex. For five complexes, conformations associated to the best FIR are represented with different colors: 1AVX (green), 1BVN (blue), 2PCC (orange), 1EWY (cyan), 1KKL (yellow). All residues with a JET+NIP score 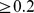 display interaction propensity and are colored in a color range going from light pink (weak signal) to deep purple (strong signal). 2PCC: the JET+NIP signal is distributed all around the receptor surface enabling different possibilities for the ligand to bind. The predicted interacting site covers only the 5% of the true binding site of the receptor. 1AVX: the predicted receptor binding site shares no residue with the real interaction site, leading to a bad prediction. 1BVN and 1KKL: despite the important size of the receptors 1BVN (496 residues) and 1KKL (3 chains of 205 residues each), corresponding binding sites are well predicted and true partners are identified with 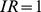 and 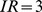 respectively.

For Enzyme-Inhibitors, Figure 6 shows that for each protein, the number of potential partners (showing an average IR 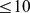 with the protein) is relatively limited. There are in average 12 such partners among the 46 tested ones. The true partner is found, in most cases (19 over 23), to have an average IR 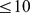 and this suggests that, in a predictive perspective, a limited set of about 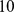 potential partners can be proposed to experimentalists with a good associated sensitivity. (See also Figure S9 in Text S1.)

#### Average IR of small versus large proteins

We can distinguish ligands from receptors with respect to the distribution of their average IR values. In fact, ligands display a higher density of good IR values (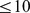) compared to receptors. This is probably due to the smaller size of the ligand and its possibility to bind to a larger number of partners. For instance, in Figure 5B, a large fraction of the surface of the small partners 1BUH_l, 1PPE_l and 1BVN_l is prone to interact (see pink regions corresponding to a high JET+NIP score) enabling multiple types of potential interactions and rendering the interaction with the true partner difficult to discriminate. At the contrary, large partners as 1N2C_r display very localized interaction sites. This observation is validated by the whole set of proteins as illustrated in Figures S38–S51 in Text S1. It should be noticed that the same observation does not hold anymore when experimental interfaces are known. This means that specific interfaces in small proteins do not glue everywhere, and therefore, that small proteins are not more promiscuous than large ones but simply that their behavior is harder to predict because of the several potential interaction sites that they might display. See Figure S54 in Text S1 for a comparative analysis of partners of small proteins when predictions and experimental interfaces are considered.

#### Species representation in the Mintseris Benchmark 2.0

**Figure 7:**
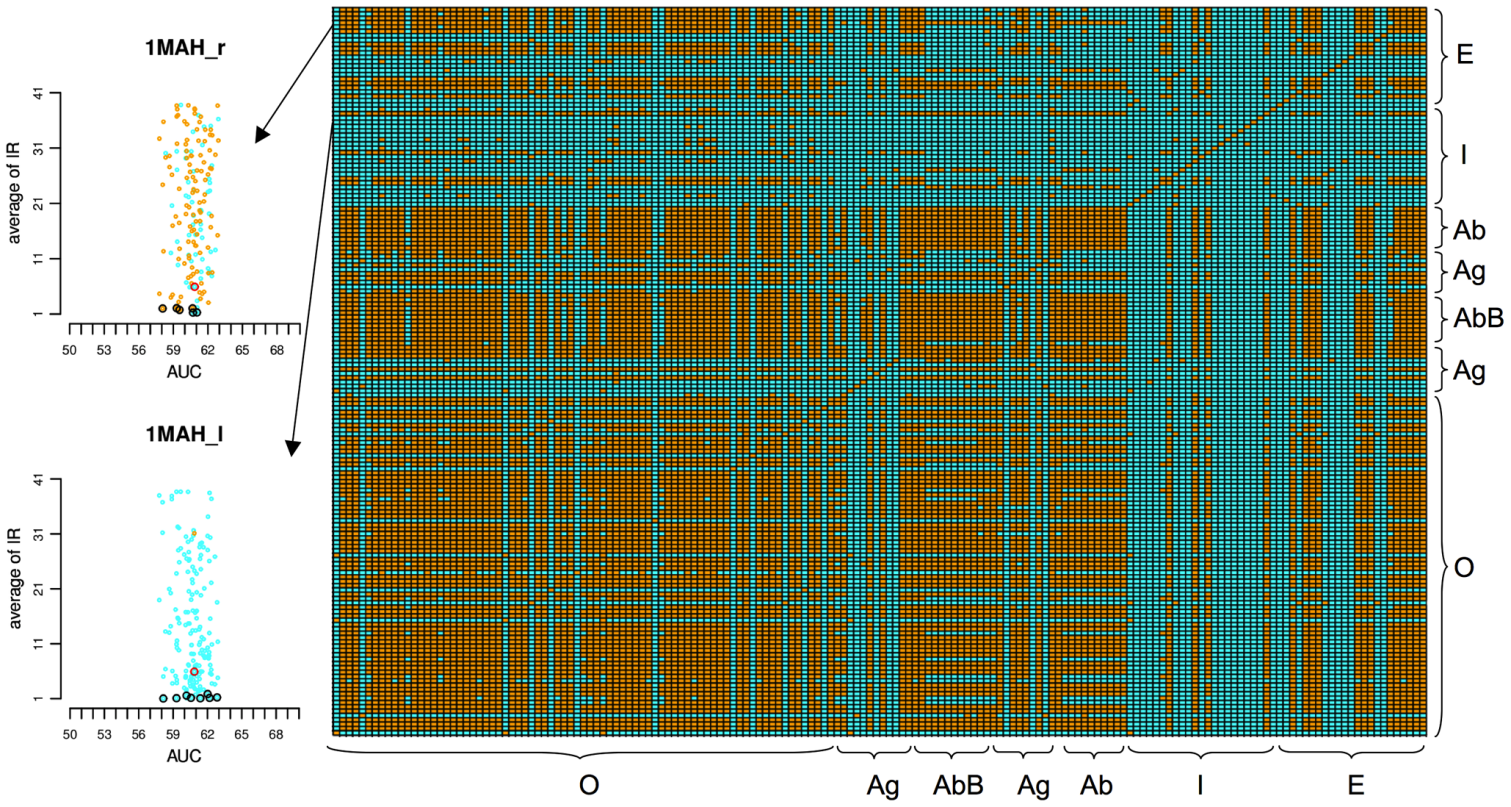
Species represented in the Mintseris Benchmark 2.0. Right: matrix reporting whether (orange entries) or not (cyan entries) any two protein structures of the Mintseris Benchmark 2.0 are represented by a common species at 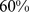 sequence identity. Each line in the matrix represents a protein and the matrix is not symmetric (see Methods). The proteins are ordered by functional classes: Others (O), Antibody (Ab), Bound Antibody (AbB), Antigens (Ag), Inhibitors (I) and Enzymes (E). The 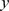-axis follows the same order as the 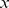-axis, from bottom to top. Compare with the matrices of Figures S74 and S75 in Text S1, based on homology computed for 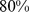 and 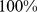 sequence identity respectively. The matrix labelled with protein names is reported in Figure S76 in Text S1. Left: an example of IRs analysis where the species information reported in the matrix on the right is plotted. Colors in the two lines of the matrix corresponding to the Enzyme-Inhibitor complex 1MAH are mapped on the dots of the plots for the receptor 1MAH_r and the ligand 1MAH_l (see legend of Figure 5 for the plots description). The black contour line on some of the proteins identifies bottom black dots in the IR analysis of Figures S38–S51 in Text S1. The red contour identifies the true interacting partner. 1MAH_r is a *Mus musculus* protein structure and 1MAH_l a *Dendroaspis angusticeps'* one, a highly venomous snake. The analysis of all proteins in the dataset is reported in Figures S60–S73 in Text S1.

In the perspective of exploring the competition among potential partners occurring in a crowded environment, we analyzed the distribution of species within the Mintseris dataset. For any pair of protein structures in the dataset, we checked whether given one of the proteins, the other has an homolog at 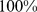, 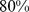 or 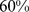 of sequence identity coming from the same species (see Methods). Such homologs are expected to display the same structure and functional characteristics of the original structure, and homologs up to 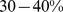 of sequence identity have been shown to interact the same way [40], [41]. When proteins are asked to be 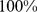 identical between species, Antibodies and Others are well represented (see Figure S74 in Text S1). When dropping the sequence identity down to 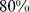 and 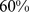, the pairs of proteins displaying homologs of the same species considerably increase in number and cover most functional classes (see orange dots in Figures S75, S76 in Text S1 and Figure 7). Notice that most of the represented species are mammalian (112 over 168; Tables S3–S6 in Text S1). This is not the case for Inhibitors which belong to species that are especially under-represented in the Mintseris dataset.

To go further in the analysis, we mapped this information on the IR plots in Figures S38–S51 in Text S1 (see Figures S60–S73 in Text S1) in order to verify whether there was a particular behavior of the proteins towards partners coming from the same species. No tendency has been observed and the uniform distribution of orange dots across the IR interval in Figures S60–S73 in Text S1 supports our hypothesis of the existence of three populations of proteins in cells (see “A protein signature based on the variability of its interaction ranks”). As an example, the enzyme 1MAH_r, coming from *Mus musculus*, shows an homogeneous repartition of the orange dots versus the blue ones (Figure 7). This holds true for all mammalian proteins (see Tables S3–S6 in Text S1) which are highly represented in the dataset. Notice that a number of proteins in the dataset are poorly represented such as the inhibitor 1MAH_l in Figure 7, a toxin protein coming from the venomous snake *Dendroaspis angusticeps*, for which all partners in the dataset come from remote species (see blue dots in Figure 7). In conclusion, despite the fact that the Mintseris dataset does not represent an actual crowded environment, the large number of shared species between proteins in the dataset and the conservation of the interaction modes between close homologs [40], [41] support the approach for exploring both protein interactions within a real environment and functional annotation.

### Comparison with other docking large-scale studies

A few large-scale studies that wish to identify true interacting partners among a set of potential ones, have been recently proposed. They are computationally demanding and they remain, for this reason, rare. All large-scale studies we compared to have been based on shape complementarity to quickly scan through several thousand ligands in a matter of seconds. These approaches do not include any electrostatic component in their energy model, while electrostatic forces are known to play an important role in PPI.

Notice that, given a protein 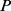, no other docking studies besides this one tries to quantify the effect of binding promiscuity of a large and variate dataset of protein structures interacting with 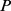.

#### Comparison with Wass et al. [33]

Docking by shape complementarity between 56 monomers (carefully) chosen from the Docking Benchmark 2.0 and a background of 922 potential interactors (excluding all partners in the Mintseris dataset) has been analyzed in [33]. A precise quantitative comparison of this computational experiment with our has been impossible because the set of protein partners of the Mintseris dataset considered in [33] is smaller and constituted only by a selected subset of receptors (with no ligand), ligands (with no receptor) and complexes (receptor and ligand) extracted from the classes Enzyme-Inhibitors and Others.

**Figure 8:**
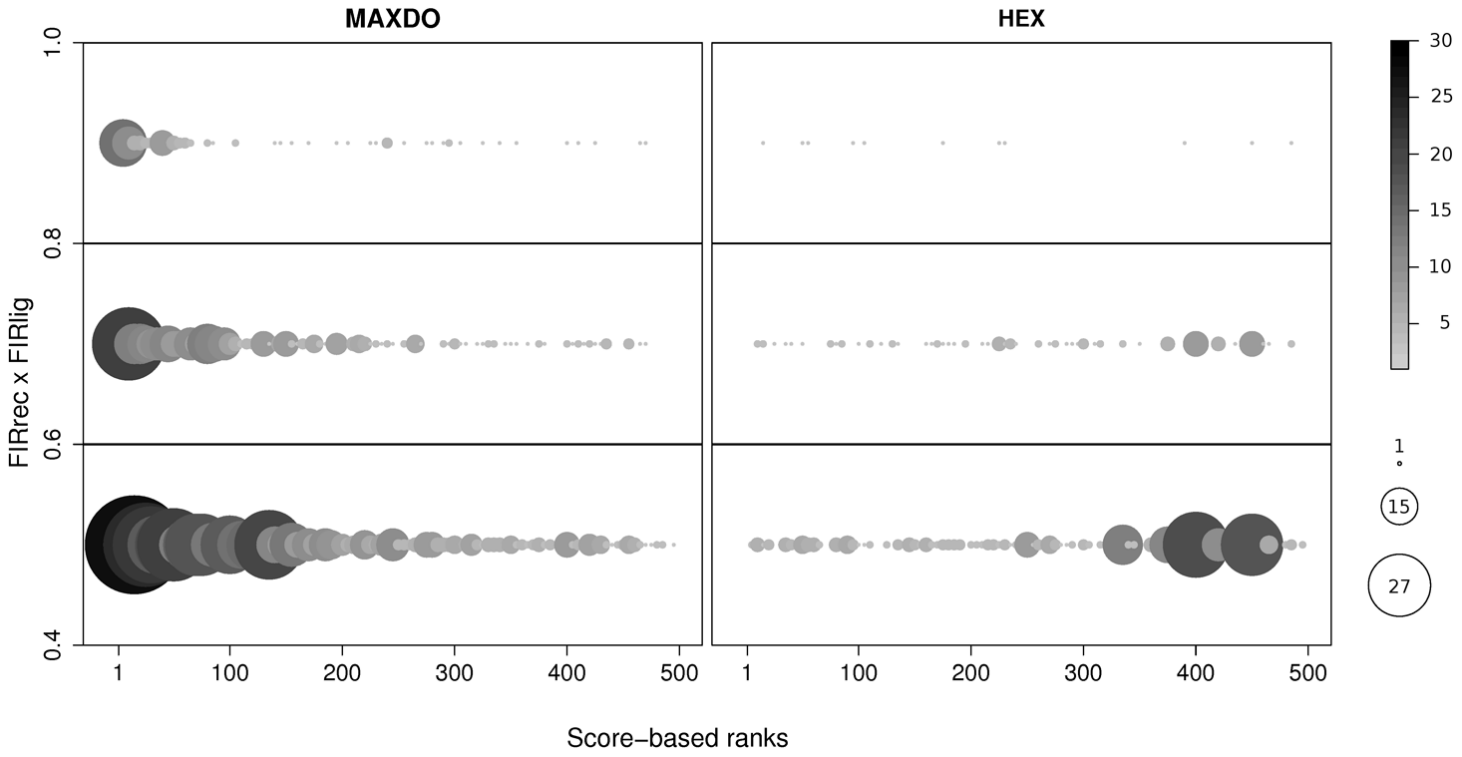
Comparison of MAXDo and HEX on the Enzyme-Inhibitor dataset. The 500 best scored conformations, computed with MAXDo and HEX, for each of the 46 native complexes in the Mintseris' Enzyme-Inhibitor dataset are plotted with respect to 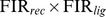 (computed on experimental interfaces; 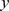-axis) and score-based ranks (computed with JET+NIP; 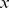-axis). The 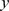-axis is defined with respect to three main intervals, 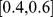, 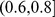 and 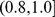, and the 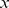-axis varies between 1 and 500. Each interval on the 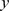-axis is associated to a distribution of ranks, where a bin in the distribution corresponds to 5 consecutive ranks. Bins are represented as circles and their sizes vary from 1 to 20. Colors are redundant with sizes.

A qualitative comparison with our predictions based on JET+NIP scores could have been made on the set of 10 complexes discussed in the SI of [33], but these results are not reproducible with more recent versions of HEX [42] (see Figure S58 in Text S1), the docking program used in [33]. Therefore, we decided to realize a CC-D with a more recent version of HEX (v6.3) on the Mintseris' Enzyme-Inhibitors dataset and to analyze HEX behavior either by assuming knowledge of the experimental interfaces or by considering predicted binding sites based on JET+NIP scores. In this latter case, NIP scores come from docking calculations using HEX. The distribution of interaction ranks for both our docking algorithm MAXDo (see Methods) and HEX are shown in Table 3, where we report how many proteins among the 46 enzymes and inhibitors are identified by each method within increasing sets of best partners, with respect to an environment of 46 proteins. From Table 3, MAXDo and HEX behave similarly on experimental interfaces while on predicted binding sites, MAXDo performance is definitely superior to the one of HEX. This shows that as the binding site prediction is not perfect, HEX is less suitable for partner identification. HEX performance has been further evaluated using ROC analysis and the AUC of the associated curve. On experimental interfaces, HEX reached an AUC of 0.81 against the AUC of 0.85 obtained with MAXDo. On predicted interfaces, HEX reached AUC values of 0.60, 0.61 and 0.60 when combined with JET+NIP, NIP and JET scores respectively, while MAXDo reached AUC values of 0.77, 0.60 and 0.72. The fact that the use of interface predictions (JET+NIP vs NIP) does not improve the AUC when using HEX, pinpoints that the conformational space of best energy solutions proposed by HEX and MAXDo are not the same. This asked for a precise analysis of the correlations between FIRs (computed on experimental interfaces and thus reflecting the overlap with experimental binding sites) and docking scores (that is, NII scores based on JET+NIP) on native complexes. We considered the conformational space of MAXDo and of HEX, each made of 11500 (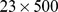) best energy conformations associated to the 23 native complexes. In Figure 8, we show the distribution of conformations for MAXDo and HEX. Two main observations can be made: first, the total number of conformations with highest FIR (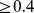) is much larger for MAXDo than for HEX, and second, among these conformations, the number of those with highest rank (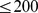) is much larger for MAXDo than for HEX. This means that the MAXDo conformational space of best energy conformations is enriched with interfaces that are close to the experimental interface, contrary to HEX.

**Table 3:** Interaction ranks distribution for the Mintseris' Enzyme-Inhibitors dataset.

	MAXDo vs HEX - Enzyme-Inhibitors dataset				
Top %	# top proteins	MAXDo		HEX	
		exp	pred	exp	pred
1	1	9 (20)	4 (9)	7 (15)	2 (4)
5	2	16 (35)	10 (22)	16 (35)	5 (11)
10	5	24 (52)	21 (46)	27 (59)	6 (13)
15	7	27 (59)	25 (54)	30 (65)	8 (17)
20	9	28 (61)	28 (61)	35 (76)	8 (17)
30	14	35 (76)	33 (72)	37 (80)	18 (39)
40	18	38 (83)	36 (78)	38 (83)	28 (61)
50	23	41 (89)	41 (89)	41 (89)	33 (72)

+JET scores obtained with weights 0.4 and 0.6 for NIP and JET respectively. For each CC-D, over the 46 Enzyme-Inhibitors in the Mintseris dataset, we report the number of proteins whose native complex is identified within the top 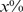 of complexes obtained by docking the protein with all 46 proteins in the environment. Cumulative counts and percentages (in parenthesis) are displayed. See legend of Table 1. CC-D has been realized with MAXDo and HEX v6.3 docking algorithms. Calculations based on predicted interfaces (fourth and sixth columns) are made with NIP

This analysis shows that shape complementarity docking is not yet ready for PPI identification, due to a currently insufficient performance of binding sites prediction methods. In fact, perfect predictions of interaction sites would strongly support the usage of docking algorithms such as HEX (Table 3), because of their computational efficiency. At the moment though, the usage of coarse-grain docking algorithms such as MAXDo, based on interaction energy scores including both Lennard-Jones and electrostatic contributions, increases manifestly the accuracy of binding partner identification compared to shape complementarity docking.

The analysis points out two more general observations. First, large environments of the order of a thousand proteins, as the one of 922 proteins considered in [33], are not useful for protein identification. To see this, we computed how many monomers among the 56 were identified by our method within increasing sets of best partners, with respect to an environment of 168 proteins. Table 1 shows that by looking at 17 best partners out of 168, we are able to identify the true partner for a fourth of the proteins in the full Mintseris dataset and for a third in the 56 proteins dataset of [33]. In contrast, in [33], it was highlighted that a third of the 56 proteins were identified by looking at 46 best partners out of 922. In practice, these results suggest that it is feasible to work with sufficiently small environments of a few hundred proteins (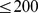) to be able to propose to the biologist a reasonable (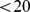) subset of proteins to test, but that large datasets, as the one considered in [33], will not be useful for protein identification unless current predictive methods improve at the point to identify most native complexes within the 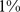 of top predictions. Possibilities for improvement exist as shown by the results based on experimental interfaces in Table 1. Notice that a perfect binding sites identification allows for the detection of native complexes for 36 out of 56 proteins within the top 17 predictions. Notice that true partners for 36 proteins are obtained in [33] by considering 184 best predictions out of 922, a set that is far too large to be experimentally tested.

The second observation concerns the composition of the set of proteins analyzed in [33]. Antibody-Antigens complexes are absent from the CC-D experiment in [33], are intrinsically difficult for interface prediction and they could constitute good test cases where both methods might highlight their respective weaknesses. Also, all 922 proteins (except 3) coming from the Mintseris or the background dataset in [33] are monomeric with two third of the background dataset having only one domain. The use of monomeric structures (especially when they are constituted by a single domain) renders the prediction easier as it is generally associated with a decrease of the number of potential interactions. Notice that our experiment is run on the full Mintseris database for which about a half of the complexes (41 over 84) involve a multimeric structure (spanning from 2 up to 4 chains), hence enhancing the difficulty of the prediction. We observe an 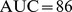 for monomeric complex predictions based on experimental interfaces, that decreases to 81 for multimeric complexes, as detailed in Table 2 and in Table S8 in Text S1. This performance on multimeric complexes is affected even more when interface predictions are considered, since sensitivity and precision of interface predictions decrease for multimeric proteins as shown in Table S11 in Text S1.

#### Comparison with Yoshikawa et al. [32]

We compared our predictions with those obtained in [32], who also studied interactions within the complete Docking Benchmark 2.0. This method, called Affinity Evaluation and Prediction (AEP), is based on shape complementarity. Contrary to our approach, (i) it indirectly uses information coming from the experimental complex, that is the bound protein structure which is expected to improve predictions, and (ii) it does not perform CC-D calculations but it only crosses the 84 receptors against the 84 ligands, by reducing in a non naive manner by the half the number of competing partners for a given protein. The complexity of the problem is, then, reduced because of the splitting between receptors and ligands that is usually not obvious to make. Even though they consider 7056 bound protein pairs while we deal with 28224 unbound ones, we obtain an AUC of 0.61 while they reach an AUC of 0.58.

### Restriction of the conformational space based on evolutionary information

The docking technique we used is computationally expensive (see “Computational implementation and data analysis” in Methods). To reduce the conformation space to be explored, we predicted the location where the interaction takes place and confined the docking to this region. This is done by predicting binding sites for the receptor protein by using JET [38] and by defining an appropriate cone around the predicted interface (see Methods and Figures S5, S6 in Text S1). When restricting the docking conformational space with JET, we observe a slight decrease of the AUC. By using experimental data, the AUC goes from 0.84 to 0.80 while using predictions, it goes from 0.61 to 0.59 (Table 4), revealing a reduced loss in precision. This shows that using evolutionary information from sequences is a very promising approach to reduce docking computational time.

**Table 4:** Partner prediction based on a restricted conformational space.

Protein dataset		Experimental interfaces			Predicted interfaces		
Subset type	# proteins	AUC	*Sen*	*Spe*	AUC	*Sen*	*Spe*
Mintseris DB	162^*^	0.80	35	95	0.59	17	90
Enzyme-Inhibitor & Others	118^*^	0.81	53	92	0.65	29	88
Enzyme-Inhibitor	44^*^	0.83	59	86	0.74	77	67
Antibody-Antigen	20	0.91	95	77	0.54	35	68
Antigen-Bound Antibody	24	0.83	50	88	0.65	12	73
Others	74^*^	0.79	55	90	0.59	34	84
Rigid	120^*^	0.81	28	96	0.54	26	82
Medium	26	0.83	73	82	0.50	19	84
Difficult	16	0.77	69	81	0.61	19	79
Monomeric (both partners)	82^*^	0.84	59	90	0.64	32	86
Multimeric (at least one partner)	80^*^	0.79	38	92	0.58	30	88

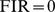
 because of no common residue between the small predicted interface and the docked one); hence, we cleaned the original Mintseris dataset of these three complexes and marked the affected subsets with the * symbol. Performance of protein prediction is evaluated through AUC values computed on the Mintseris dataset and its different subsets. Sensitivity (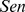) and specificity (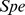) are also given at a threshold cutoff of 0.5 for predictions based on experimental interfaces, and at a threshold cutoff of 0.25 for predicted interfaces. Calculations based on JET predicted interfaces use weights 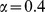, 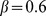 (see Methods), with the exception of the analysis run for Antibody-Antigen and Antigen-Bound Antibody where 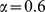, 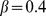. The analysis is realized by assuming knowledge of either the experimental interfaces or the predicted interfaces. In both cases, we report the results obtained on the restricted (by evolutionary information) conformational space. On three complexes (1ML0, 1GCQ, 1DFJ), JET provided too small interaction sites (leading to a

To evaluate the impact of our restriction on MAXDo execution time, we computed how many docked conformations between protein pairs were dropped. When the 168 proteins are considered together, the average portion of the conformational space that is explored after reduction is 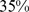 of the original space. This value should be understood at the light of protein sizes, as illustrated in Figure S59 in Text S1. In fact, small proteins require to explore about 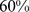 of their original conformational space, while for large ones, the space is reduced to 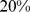 of the initial one. This is because small proteins are rather conserved and JET predicts large patches as their interaction sites, covering a large portion of their protein surface. Notice that this calculation takes into account a reduced number of conformations for the receptor, independently on whether the conformational space of the ligand is completely explored or not. Clearly, the actual computational time depends on the number of conformations that are tested, and if both the conformational spaces of the receptor and of the ligand are reduced, the effect will be quadratic. The small difference in AUC obtained by exploring the reduced space of the receptor compared to the whole (with a fully explored surface of the ligand), is due to the high specificity of JET and to the definition of the cone (see Methods) that takes into account JET's lower PPV.

## Discussion

We have addressed the problem of predicting protein interactions using high-throughput CC-D calculations on a dataset of 168 proteins. We have shown that a simple docking algorithm combined with evolutionary information, can be used to discriminate interacting from non-interacting proteins. The purpose of the method is the *in silico* large-scale screening of protein structures to find a small set of potential protein partners that could be tested experimentally. The approach reminds the one of drug design aiming to screen large sets of small molecules in order to identify a small set of potential drugs that becomes experimentally testable. These approaches do not pretend to exactly identify a unique solution but rather a set of reasonable candidates, and reduce, in this manner, the amount of experimental time and costs. This means that we are not focused on the correct docking of experimentally known partners, which can be achieved via other more effective but much more computationally demanding methods [43]. However, one can envisage to use such more sophisticated methods on the small set of candidates that our coarse method identifies to propose more precise models of the potential complexes.

We have realized a large-scale PPI analysis by assuming to know the residues forming the experimental interface of the native complexes (no associated experimental conformation is considered) and by using predictions of binding sites. Experimental binding sites can be seen as perfect predictions, and the analysis based on them is realized for two reasons: 1. to understand how much evolutionary information can contribute to PPI reconstruction when coupled with a coarse-grain docking algorithm using an energy function, and 2. to decouple true PPI signal from noise and identify PPI properties that are not consequences of accumulated errors due to predictive algorithms. This second reason allowed us to be confident, for instance, on the promiscuity observed in Figure 5B (bottom black dots) by ensuring that it is not generated by noise in predictions (see Figure 5A).

A few large-scale analyses, that are similar in spirit, have been performed [32], [33]. A comparison of our results with [33], based on the ten protein complexes discussed in detail in [33], reveals a similar performance of the two methods. However, a full comparison with [33] is impossible since they treat only a subset of the Mintseris dataset, use a large background set and do not provide a detailed measure of the performance of their method. On the contrary, our method is tested on all complexes of the Mintseris dataset, a good testing platform for methods dedicated to protein partner prediction due to its numerous structural differences. The global analysis of the two methods (over the subset of 56 proteins; see Table 1 and [33]) highlights that we can reasonably search for protein partners within sets of a few hundred monomers. We demonstrated that improving current predictive methods is possible through a better prediction of binding sites, and we precisely estimated the effect of such predictions.

We could only partially compare to [32] since they do not perform a CC-D of the Mintseris dataset but only cross the 84 receptors against the 84 ligands, that is a fourth of the interactions explored in our analysis. Performances of our method and the one reported in [32] are comparable on the common subset, but notice that contrary to [32], we use unbound structures and we make no use of the non-naive split of the dataset (that is, receptors versus ligands).

The predictive performance of the method is encouraging for the whole Mintseris Benchmark 2.0 and very satisfactory for the enzyme-inhibitor subset (Table 2). For this latter, the AUC reaches a very high value of 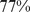 while the AUC for the whole Mintseris dataset is 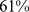. Notice that the way we computed the AUC is very strict, since we asked the true partner to be ranked first over the tested dataset. A more relaxed evaluation is reported in Table 1 where we show that a fourth of the 168 proteins in the Mintseris dataset are recognized by looking at the top 17 predictions over the 168 tested partners. If the binding site of the proteins is correctly predicted, the half of the proteins in the dataset are recognized by looking at the top 8 predictions, and two third by looking at the top 17. This is a very encouraging result with respect to the potential applicability of this *in silico* predictive approach to the reconstruction of PPI networks. In fact, proposing to a biologist a set of less than 20 interactions to test is very reasonable.

The analysis on the average IR for the enzyme-inhibitor subset highlights that an average IR threshold 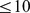 allows the method to propose about 12 partners, a reasonable number of proteins to be selected for experimental tests. In 38 cases over 46 (Figure 6), the true partner is present in the retained subset showing a very high sensitivity. For the whole Mintseris benchmark, for roughly the half of the dataset (82 proteins), the true partner is retrieved with an average IR 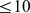. Notice that when considering the experimental binding site of each partner, 138 proteins over 168 display an average IR 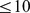. This means that a precise binding site prediction method will lead to a successful partners discrimination, a problem that could be considered as being much more ambitious than the binding site prediction problem. Again, these results support the feasibility of the approach to identify potential partners but, most of all, they highlight the interest of testing a protein within a large environment, by randomly choosing many small subsets of proteins in the environment, and by selecting as potential partners to be experimentally tested, those proteins that present a stable average IR 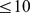 (black dots, Figure 5) with the protein under study. The selection of 10 potential partners instead of 17 (as suggested by the direct evaluation of the NII matrix in Figure 1 and Table 1) might be crucial for experimental validation. This observation opens a way to new computational schema for partner predictions.

The analysis highlights an important point on the behavior of all proteins with respect to their partners. For each protein, there is a small set of partners that displays a systematic (black points in the bottom of Figure 5AB) very low average IR that lead to ask whether these partners might physically interact and not be false positives. Three reasonable explanations for this set of highly potential partners can be given: (i) partners can interact on a merely physical base but never meet in the cell due to different cellular compartments localization, (ii) partners can interact for functional purposes, possibly not described until now (several different partners are expected to interact with a protein), (iii) partners can interact in the cell not for functional purposes but generating a competition with the functional partner, possibly participating to the regulation of the protein interactions in the cell. Taking into account these possibilities, this set of highly potential partners becomes interesting for further studies. For instance, these interactions would deserve to be experimentally tested to see how strongly they interact, and whether they form a structurally well-defined complex. Also, for a given protein and a set of highly potential partners, one could ask whether general structural (geometrical or physico-chemical) features of the interface exist and in the positive case, classify these interfaces. These further studies could contribute to give important insights into protein partnership discrimination.

For each protein 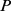, we defined a signature representing the strength of interaction of 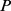 with all other proteins. As mentioned above, signatures found for all 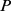
's in the Mintseris dataset demonstrate the existence of strong interactions with some proteins, but also the absence of interactions with other proteins, and so on. The spectrum of strengths of interactions suggests the notion of PPI to be revisited so to include the larger panel of potential complex formations between a protein and its potential partners. Several questions could then be asked on proteins presenting similar signatures [44], but they go beyond the aim of this work.

We have shown that evolutionary information can also be used to restrict the conformational space of the docking exploration without an important loss in sensitivity. This result is very important in view of reducing the computational cost of highly time demanding docking calculations (all atom description and precise energy functions) and the perspective of enlarging the dataset size for future CC-D calculations.

To conclude, we are the first to perform a CC-D of a pool of proteins covering a large spectrum of functions and interaction modes, performing it on unbound structures and providing energy values (even though simplified) taking into account electrostatic forces. Our approach is the first combining evolutionary information with CC-D simulations. The evaluation of the performance of these two contributions to the problem of partner identification, suggests that there is still room for improvement in the solution. In particular, we have shown that a precise identification of protein binding sites allows for very satisfactory predictions. Data coming from the CC-D calculations and the evolutionary analysis are provided and they will help the community to evaluate further CC-D studies and methodological developments. In particular, the decoy set constitutes a unique dataset of “negative” partners. For them, we provide about 2000 conformations and an associated coarse-grain energy score. It might be extremely useful to suitably parametrize docking scoring functions, more refined than our coarse-grain scoring function, to discriminate partners. In the context of this study, a subset of these decoy structures filtered by our coarse-grain scoring function could be re-scored for a better partnership evaluation by using a more refined score function better discriminating the interaction signals.

## Methods

### The protein dataset

The Docking Benchmark 2.0 [25] is constituted by 168 proteins belonging to 84 known complexes. We used the unbound conformations of the proteins with the exception of 12 antibodies for which the unbound structure is unavailable. For those, the bound structure is used instead. Any reference to the proteins uses either their name or the Protein Data Bank (PDB) code [45] of the experimental complex they belong to with the 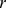 or 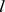 extension denoting a receptor or a ligand protein respectively. For example, 1AY7_r and 1AY7_l refer to barnase (receptor) and barstar (ligand) in the barnase-barstar complex 1AY7. The coordinates for the bound and unbound structures of both receptor and ligand proteins are available in the PDB and can be found at http://zlab.bu.edu/zdock/benchmark.shtml.

### The docking algorithm

Molecular docking is performed with the MAXDo (Molecular Association via Cross Docking) algorithm, developed for complete cross-docking (CC-D) studies [12]. Since CC-D involves a much larger number of calculations than simple docking, we chose a rigid-body docking approach using a reduced protein model in order to make rapid conformational searches.

#### A reduced protein representation

We used a coarse-grain protein model developed in [46], where each amino acid is represented by one pseudo-atom located at the C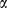 position, and either one or two pseudo-atoms representing the side-chain (with the exception of Gly). Ala, Ser, Thr, Val, Leu, Ile, Asn, Asp, and Cys have a single pseudo-atom located at the geometrical center of the side-chain heavy atoms. For the remaining amino acids, a first pseudo-atom is located midway between the C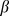 and C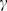 atoms, while the second is placed at the geometrical center of the remaining side-chain heavy atoms. This description, which allows different amino acids to be distinguished from one another, has already proved useful in protein-protein docking [46]–[48] and protein mechanics studies [49], [50]. Interactions between the pseudo-atoms of the Zacharias representation are treated using a soft LJ-type potential with appropriately adjusted parameters for each type of side-chain, see Table 1 in [46]. In the case of charged side-chains, electrostatic interactions between net point charges located on the second side chain pseudo-atom were calculated by using a distance-dependent dielectric constant 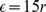, leading to the following equation for the interaction energy of the pseudo-atom pair 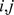 at distance 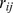:
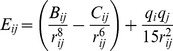
(2)where 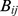 and 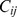 are the repulsive and attractive LJ-type parameters respectively, and 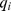 and 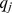 are the charges of the pseudo-atoms 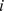 and 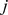.

#### Systematic docking simulations

Our docking algorithm (see Figure S52 in Text S1) was derived from the ATTRACT protocol [46] and uses a multiple energy minimization scheme. For each pair of proteins, the first molecule (called the receptor) was fixed in space, while the second (termed the ligand) was used as a probe and placed at multiple positions on the surface of the receptor. The initial distance of the probe from the receptor was chosen so that no pair of probe-receptor pseudo-atoms came closer than 6 Å. Starting probe positions were randomly created around the receptor surface with a density of one position per 10 Å^2^, and for each starting position, 210 different ligand orientations were generated, resulting in a total number of start configurations ranging from roughly 100,000 to 450,000 depending on the size of the receptor. During each energy minimization, the ligand protein was kept at a given location over the surface of the receptor protein, using a harmonic restraint to maintain its center of mass on a vector passing through the center of mass of the receptor protein. The direction of this vector was defined by two Euler angles 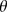 and 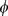, (where 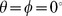 was chosen to pass through the center of the binding interface of the receptor protein) as shown in Figure S52 in Text S1. By using a Korobov grid [51] and varying the Euler angles from 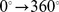 and 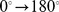 respectively, it was possible to uniformly sample interactions over the complete surface of the receptor and to represent its binding potential using 2D energy maps (each point corresponding to the best ligand orientation for the chosen 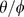 pair). These maps where developed for validating the docking algorithm [12].

#### Computational implementation and data analysis

Each energy minimization for a pair of interacting proteins typically takes 15 s on a single 2 GHz processor. As noted above, approximately 100,000 to 450,000 minimizations are needed to probe all possible interaction conformations, as a function of the size of the interacting proteins. Therefore, a CC-D search on the benchmark, namely 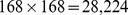 receptor/ligand pairs, would require several thousand years of computation on a single processor. However, since each minimization is independent of the others, this problem belongs to the “embarrassingly parallel” category and is well adapted to multiprocessor machines, and particularly to grid-computing systems. Our calculations have been carried out in 2007 by the public World Community Grid (WCG, www.worldcommunitygrid.org), with the help of thousands of internautes donating their computer time to the project. It took approximately seven months to perform CC-D calculations on the complete dataset of 168 proteins. More technical details regarding the execution of the program on WCG can be found in [52]. The data analysis was partly realized on Grid'5000 (https://www.grid5000.fr).

### Definition of surface and interface residues

Surface residues are residues with at least 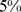 of accessible surface. Accessibility is calculated with NACCESS 2.1.1 [53] with a probe size of  = 1.4 Å. Interface residues are residues with a change of at least 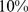 decrease in accessible surface area compared to the unbound protein.

### Protein interaction index and its normalization

In order to improve the quality of the predictions of protein interaction partners, in our earlier study we developed a normalized interaction index (NII) that takes into account whether a protein-protein interface involves amino acids belonging to a known interaction site [12]. This information can potentially be obtained using predictive tools (see below), but here we use the experimentally determined interfaces of the 84 binary complexes in the Docking Benchmark. We however recall that all our docking trials involve unbound protein conformations. For each protein partner in a given complex 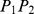, we determine which fraction of the docked interface residues (abbreviated as 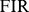) are found in the experimental interface for 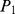 (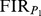) and 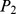 (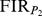). Thus defining an overall fraction for the complex as 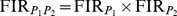. It is important to notice that the 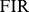 formula can be computed from either experimental interfaces (as defined above) or predicted interfaces (where prediction could be realized, for instance, with evolutionary information; see paragraph below). The notion of “FIR” proposes a new concept for docking evaluation that can be used as an alternative to the usual docking metrics 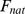 [54] originally designed to evaluate the accuracy of pairwise protein docking models. While the 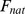 measure denotes the coverage of the experimental interface, that is the sensitivity of the predicted interface, the FIR denotes the PPV of the predicted interface. Also, for the 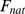 measure, contacts are defined with respect to a 5 Å cutoff on the RMSD of heavy atoms, while for FIR, contacts are defined from a change of solvent accessibility.

For every protein pair 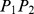, we calculate an energy-weighted optimal interaction index (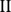) defined in Eq. (1).

To allow comparison among different partners we defined a normalized index 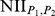 by taking into account all of the four lines/columns that feature either 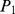 or 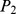 in the 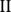 matrix as follows:
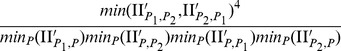
(3)where 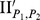 is a symmetrized version of the interaction index 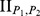 and it is defined as:
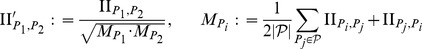
(4)where 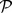 are the 168 proteins of our dataset. 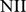 values vary between 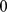 and 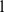. Values close to zero imply that two proteins cannot form an interface involving a significant fraction of the experimentally identified residues, or that interfaces involving these residues have poor interaction energies. Values close to one indicate predicted interfaces with good energies and composed of experimentally identified residues.

For each protein 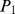, we define as *predicted partner of* 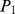, the protein 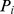 that leads to 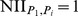.

### Partner prediction evaluation

We consider as true positives (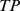) all the predicted pairs that belong to the Docking Benchmark 2.0 and as true negatives (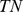) all the pairs that are correctly predicted as non interacting. We define a False Positive Rate (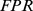) and the True Positive Rate (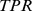) to be 
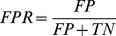
 and 
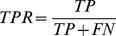
, where 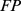 is the set of False Positives (partners incorrectly predicted as interacting) and 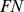 is the set of False Negatives (partners incorrectly predicted as non interacting). The computation of 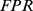 and 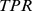 for various thresholds enables the Receiver Operating Characteristics (ROC) curve to be drawn. The performance of the prediction is given by the resulting AUC (Area Under the Curve) value. Values of 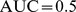 and 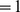 correspond to random and perfect predictions respectively. AUC calculations were performed with the R package [55]. Also, given a threshold on the NII values, we use five standard measures of performance: sensitivity 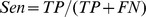, specificity 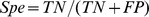, precision or positive predictive value 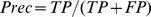, balanced 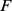-score 
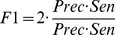
 and Matthews correlation coefficient 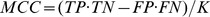 where 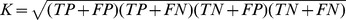.

### Prediction of partners based on docking and evolutionary information

To predict protein partners without using any experimental information, we define a new FIR measure by combining docking and evolutionary information. From FIR values, NII matrices are computed as above.

#### Residue scoring based on docking

In order to see whether CC-D simulations could give us information on protein interaction sites, we developed an energy-weighted interaction propensity (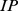) index which estimates the probability for residue 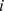 of protein 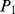 to belong to an interaction site (without hypothesis on the corresponding partner). For doing this, similarly to [24], we counted the number of docking hits for each exposed residue in 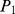, that is the number of times that a residue is seen in interaction with all the docked partners within a range of best energy conformations. Namely, for each arbitrary partner 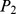, all energies between 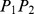 conformations are first normalized according to a Boltzmann weight that favors conformations with the most negative interaction energies:
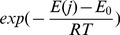
(5)where 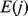 is the interaction energy in conformation 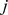, 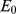 is the lowest interaction energy obtained for the 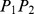 complex, 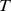 is the temperature (300 K), and 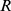 is the gas constant. These normalized values, named *Boltzmann normalized energies*, range from 0 to 1. All conformations with Boltzmann normalized energy above 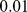 (this value corresponds to an energy difference from the best one of 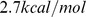) have been retained for the pair 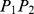. Finally, each surface residue 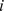 in 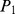 is scored by the total number of times it appears in the interface of the retained conformations involving all partners 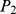, normalized by the total number of retained conformations:
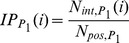
(6)where 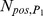 is the number of retained conformations of 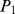 and 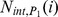 is the number of these conformations where residue 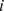 belongs to the binding interface.

To allow comparison between residues belonging to the same protein 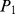, the index 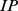 can be normalized as
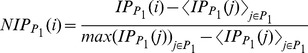
(7)where 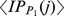 is the average computed over all residues 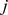 at the surface of 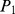, and 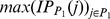 is the maximum 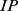 value obtained at the surface of 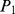. NIP can be positive, indicating that residue 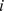 is favored to occur at 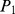 potential binding sites, or negative, indicating that it is disfavored. We used 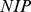 as a parameter for the prediction of protein binding sites, dividing the residues into two groups: 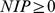 predicted as belonging to the binding interface; 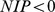 predicted as not belonging to the binding interface.

#### Residue scoring based on evolutionary information

Protein interfaces are predicted with the Joint Evolutionary Trees (JET) method [38]. JET is a large-scale method designed to detect very different types of interactions. It predicts interface patches for protein families by combining residue conservation with physico-chemical properties expected at the protein interfaces. Conserved patches are then extended by using heuristics leading to alternative interaction sites for different JET runs. For each protein 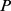, 10 runs of JET were launched and we defined a score 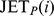 for each surface residue 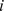 to be the number of occurrences of the residue in extended patches divided by 10. This score reveals the likelihood of the residue to belong to the interface.

#### Predictions by combining docking and evolutionary information

For each residue 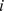 in protein 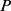, we define a score 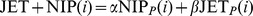, where 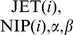 range between 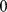 and 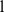. Different combinations of 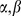 have been tested and our final results are obtained with 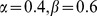. Since Antibody-Antigens evolve more rapidly than other interface surfaces, the conservation signal is less sharp and predictions are intrinsically difficult. For this, we lowered the weight of JET contribution by fixing 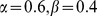. Evaluation of the performance of the score “
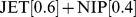
” for partner prediction compared to the performance of NIP alone or JET alone on the Mintseris dataset and its subsets is illustrated in Figure S77 and Table S10 in Text S1.

Based on the 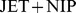 score, we can associate a 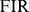 value to a docked interfaces 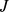 between 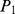 and 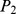. As above, we select those conformations of 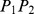 with Boltzmann normalized energy 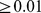 and compute their 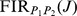 as:
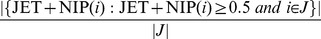
(8)where 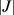 is the number of residues in the interface 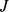. Namely, we count the number of residues 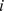 whose score 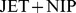 is 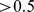, that is the residues that display either a very good score obtained with one of the two methods (based on docking or on evolutionary information) or relatively good scores with both. These residues are likely to belong to an interaction site.

Like previously, in all protein pairs 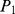 and 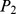, we can compute a 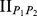 and a 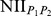. The 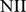 matrices are evaluated by computing their AUC.

### The interaction rank

The interaction rank of a protein pair 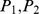 is defined to be the best rank of the pair 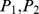 among all the pairs that have either 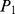 or 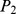 as receptor. This means that given a 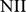 matrix, we look at the rank of the pair 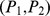 with respect to the 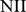 values 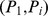, that is the line indexed by 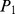, and at the rank of the pair 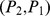 with respect to the 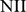 values 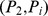, that is the line indexed by 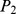. The best rank computed for each line is retained for the pair 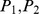.

### Cone definition for the conformational space restriction

To restraint the conformational space of the docking algorithm, we combine JET interface predictions with MAXDo, in such a way that only surface regions containing residues predicted by JET will be analyzed by MAXDo. To do so, for each docked orientation, we computed the center of mass of the ligand and defined the axis linking it to the center of mass of the receptor. We remind that the position of the receptor is fixed. Along this axis, we define an imaginary tube of radius *r* = 2.9 Å. For each ligand orientation, we check whether the interface of the resulting ligand-receptor complex involves residues predicted by JET or not (Figure S53 in Text S1). Each residue is approximated to a point whose coordinates represent the average of the atom's coordinates. The distance of this point from the axis of the tube, allows to establish whether the residue falls inside the tube or not, and therefore, whether the ligand orientation should be retained or not. Strictly speaking, one should also use the scalar product between the vectors going from the receptor center of mass to the residue and to the ligand center of mass (this product decides whether the residue lies on the side of the ligand-receptor interface). We ask for just one single residue in the orientation interface to be within the tube to retain this latter.

### HEX docking

CC-D of the Mintseris' Enzyme-Inhibitors dataset was performed with HEX v6.3 using the shape complementarity based-only score [42]. Docked conformations were clustered using a 3 Å cutoff and the best-scored conformations of the 500 first clusters were retained for the analysis. A protocol similar to that described for MAXDo was applied to evaluate partner prediction based on HEX results, (i) by assuming knowledge of the experimental interfaces and (ii) by crossing docking scores with evolutionary information. All 500 conformations were considered for residue scoring based on docking and for protein interaction index calculation. Parameter values are reported in Table S7 in Text S1.

### Analysis of the origins for the proteins in the Mintseris dataset

Given a protein 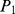, we searched in the Mintseris dataset for those proteins 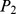 that have a homolog coming from the same species as 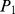. Namely, for each 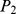, we searched with Blast (E-value threshold at 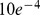, alignment coverage 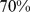) for the set of sequences that are at least 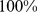, or 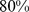 or 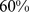 identical to the 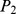 original sequence. This provides a set of species that we say to be representing 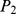. We then checked that the species of 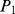 is included in the set of the species representing 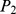. Notice that the protocol does not necessarily provide the same answer when it is applied to the protein pairs 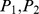 or 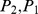 due to the non-symmetrical Blast result.

### Data release

We release the first large decoy database comprising not only structures of true complexes but also structures of non-functional complexes potentially forming in the cell. For the 28224 possible protein pairs (involving the 168 proteins) of the Mintseris Benchmark 2.0, we considered about 2000 best ligand orientations (represented on 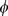 and 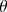 angles as described above) for each receptor. We provide the associated decoys together with the corresponding energy values. A program to reconstruct the PDB structure of the conformation given 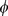 and 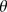 angles is also provided. For each protein in the Mintseris dataset, we also furnish the evolutionary analysis for the detection of the binding sites. The download site is http://www.lgm.upmc.fr/CCDMintseris/

## Supporting Information

Text S1 Supplementary Figures S1–S77 and Tables S1–S11.
(PDF)
