# Supplementary material for: Protein-Protein Interactions in a Crowded Environment: An Analysis via Cross-Docking Simulations and Evolutionary Information
Source: PLoS Comput Biol. 2013 Dec 5;9(12):e1003369. doi: 10.1371/journal.pcbi.1003369 (PMC3854762; doi:10.1371/journal.pcbi.1003369)
Supplement: Text S1 — Supplementary Figures S1–S77 and Tables S1–S11. (PDF) [file pcbi.1003369.s001.pdf]

Text S1

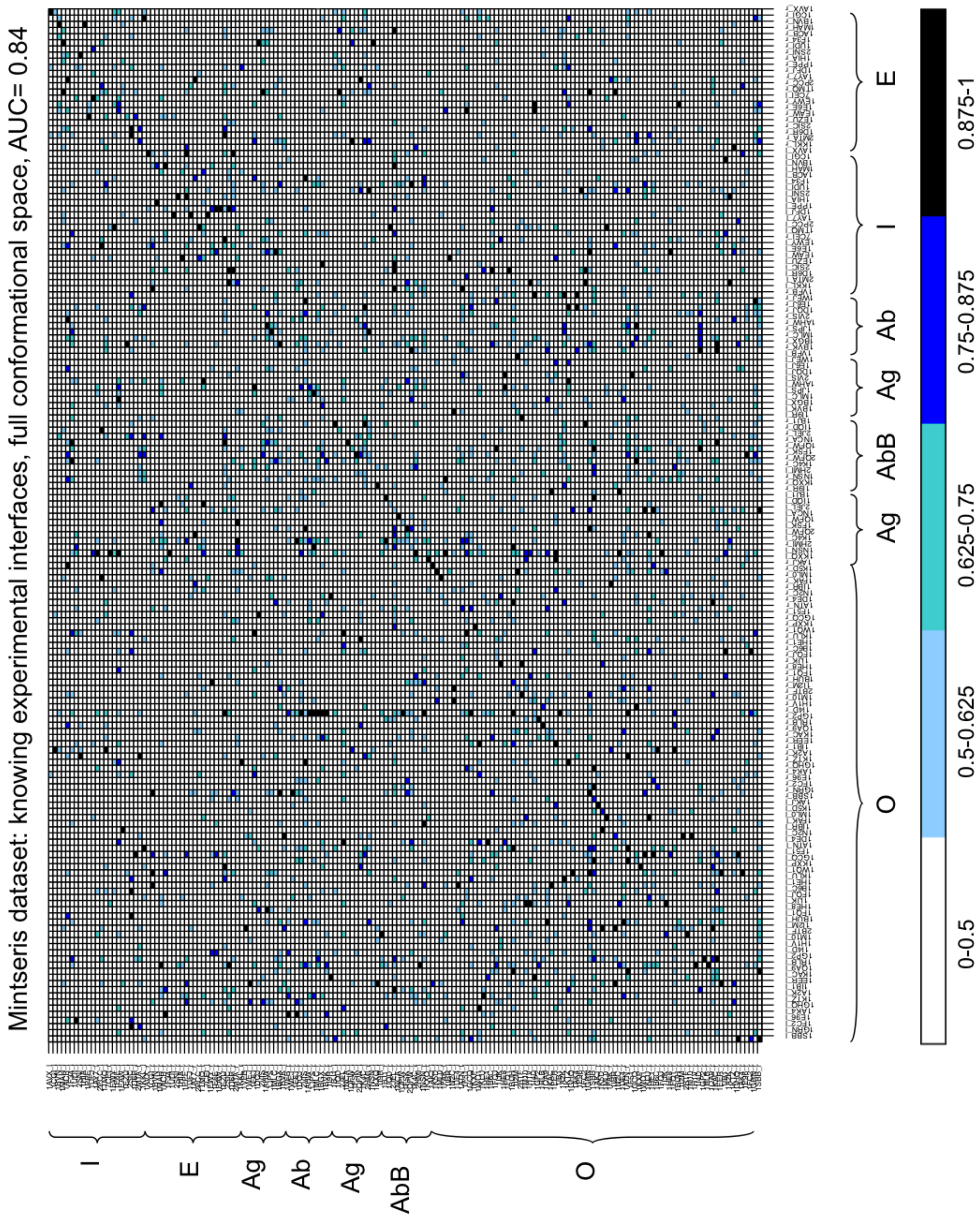

Figure S1: **NII matrix for the Mintseris Database 2.0 - experimental interfaces computed over the full conformational space**. The matrix is ordered with the experimental complexes lying on the trailing diagonal. Protein structures labelling columns and rows are grouped in functional classes: Enzymes (E), Inhibitors (I), Antibodies (Ab), Antigens (Ag), Bound Antibodies (AbB), Others (O). Each entry of the matrix corresponds to the NII value computed for the corresponding pair of proteins. High interaction scores (between 0.7 and 1, blue and black in the color scale) indicate a high interaction probability. Interaction scores are computed using knowledge of the experimental interfaces. The matrix has AUC=0.84.

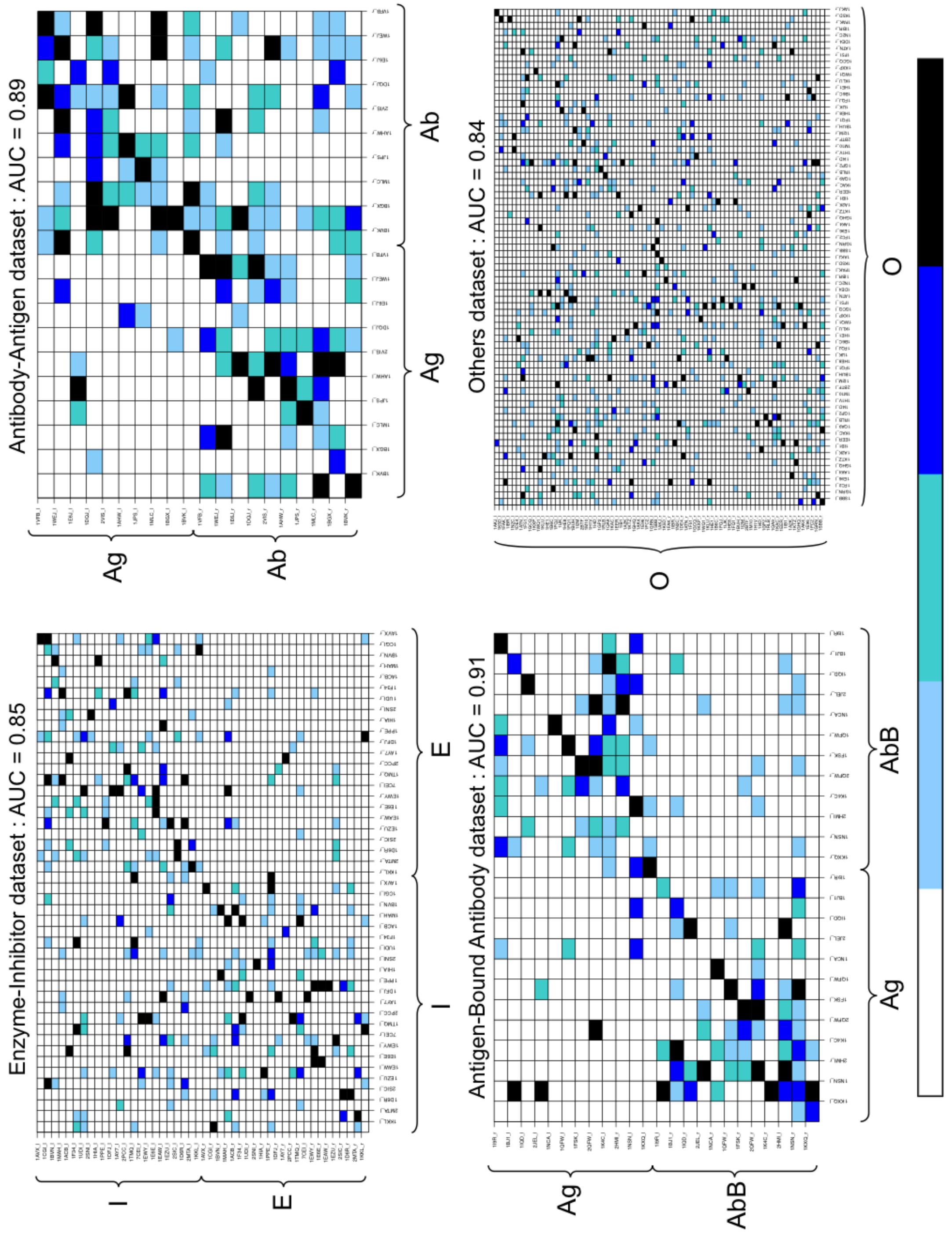

Figure S2: **NII matrices for functional classes of proteins - experimental interfaces computed over the full conformational space**. Enzyme-Inhibitors (EI; top left), Antibody-Antigen (AbAg; top right), Antigen-Bound Antibody (AgAbB; bottom left), Others (O; bottom right). Matrices are ordered with the experimental complexes lying on the trailing diagonals and, for each matrix, protein structures are grouped in functional classes. Each entry of a matrix corresponds to the NII value computed for the corresponding pair of proteins. High interaction scores (between 0.7 and 1, blue and black in the color scale) indicate a high interaction probability. Interaction scores are computed using knowledge of the experimental interfaces.

Rigid dataset: knowing experimental interfaces, full conformational space, AUC = 0.87

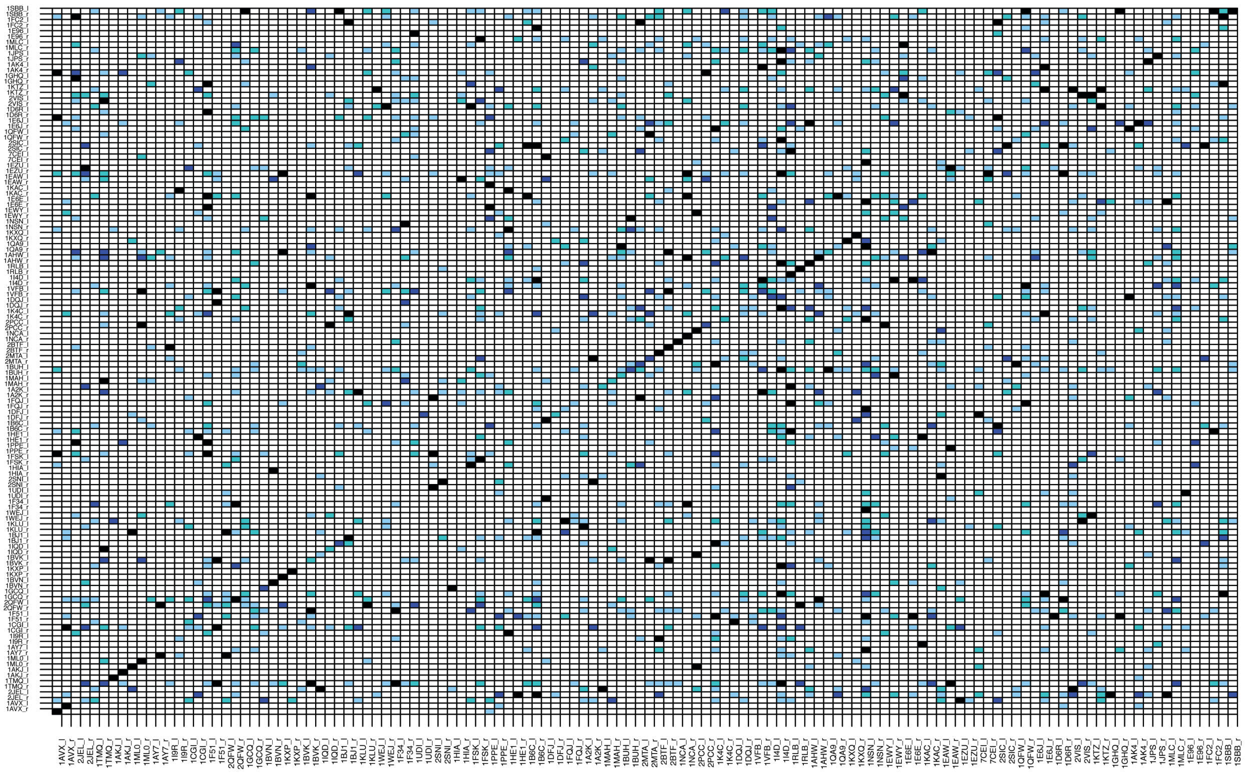

Figure S3: **NII matrix for Rigid proteins - experimental interfaces computed over the full conformational space**. The matrix is ordered with the experimental complexes lying on the trailing diagonal. Each entry of the matrix corresponds to the NII value computed for the corresponding pair of proteins. High interaction scores (between 0.7 and 1, blue and black in the color scale) indicate a high interaction probability. Interaction scores are computed using knowledge of the experimental interfaces. The matrix has AUC=0.87.

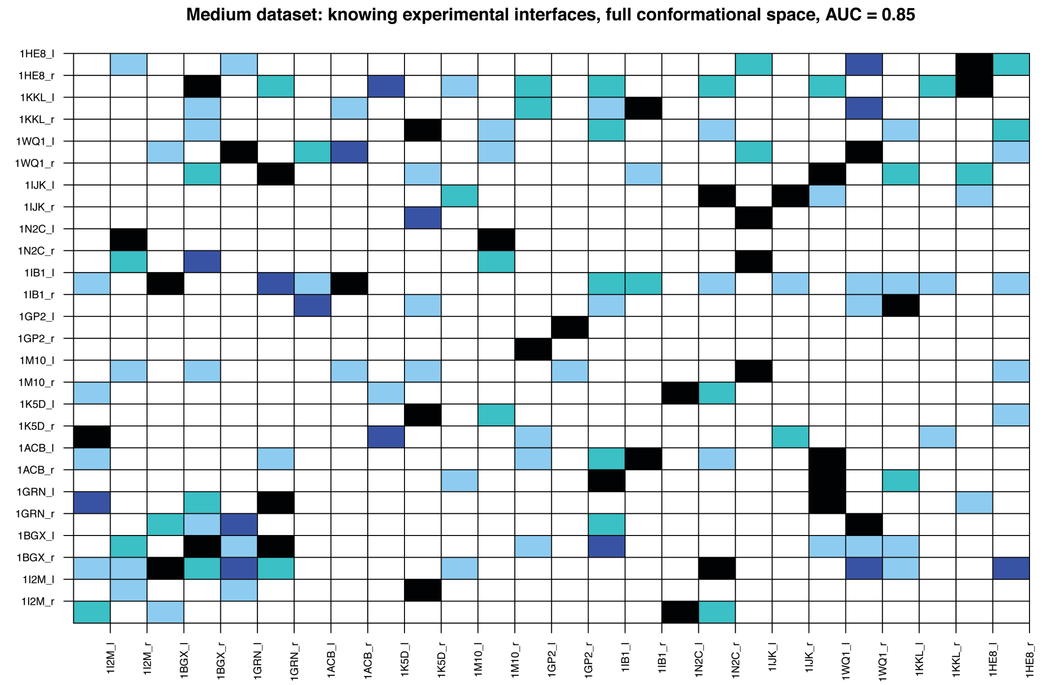

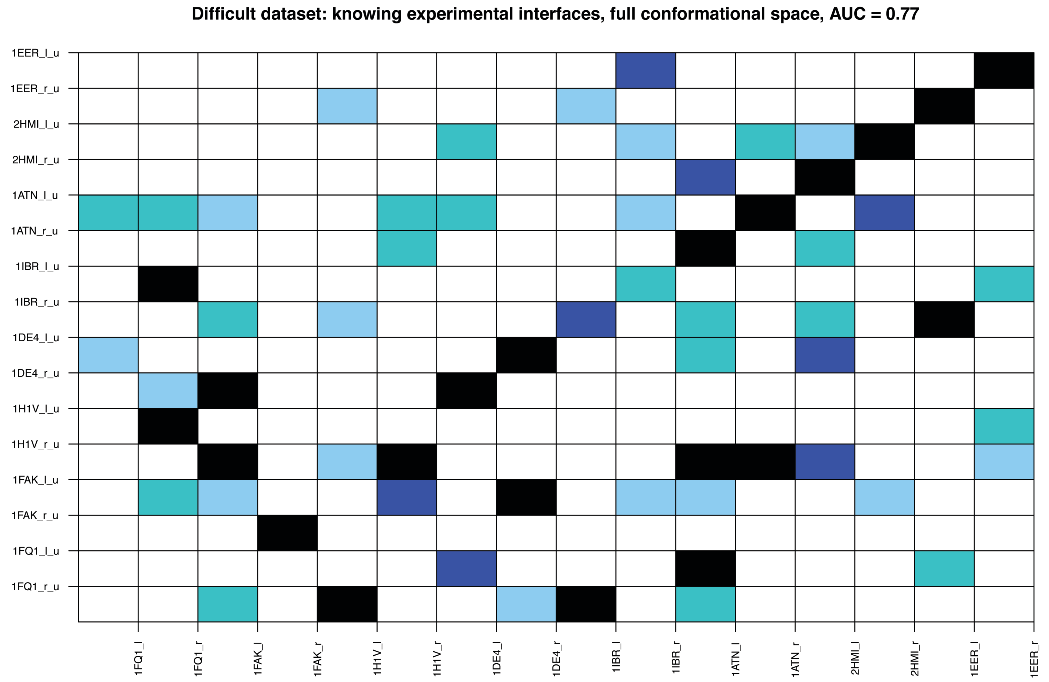

Figure S4: **NII matrix for Medium (top) and Difficult (bottom) proteins - experimental interfaces computed over the full conformational space**. Matrices are ordered with the experimental complexes lying on the trailing diagonals. Each entry of a matrix corresponds to the NII value computed for the corresponding pair of proteins. High interaction scores (between 0.7 and 1, blue and black in the color scale) indicate a high interaction probability. Interaction scores are computed using knowledge of the experimental interfaces. The matrix of medium interfaces has AUC=0.85, and the one with proteins with difficult interfaces has AUC=0.77.

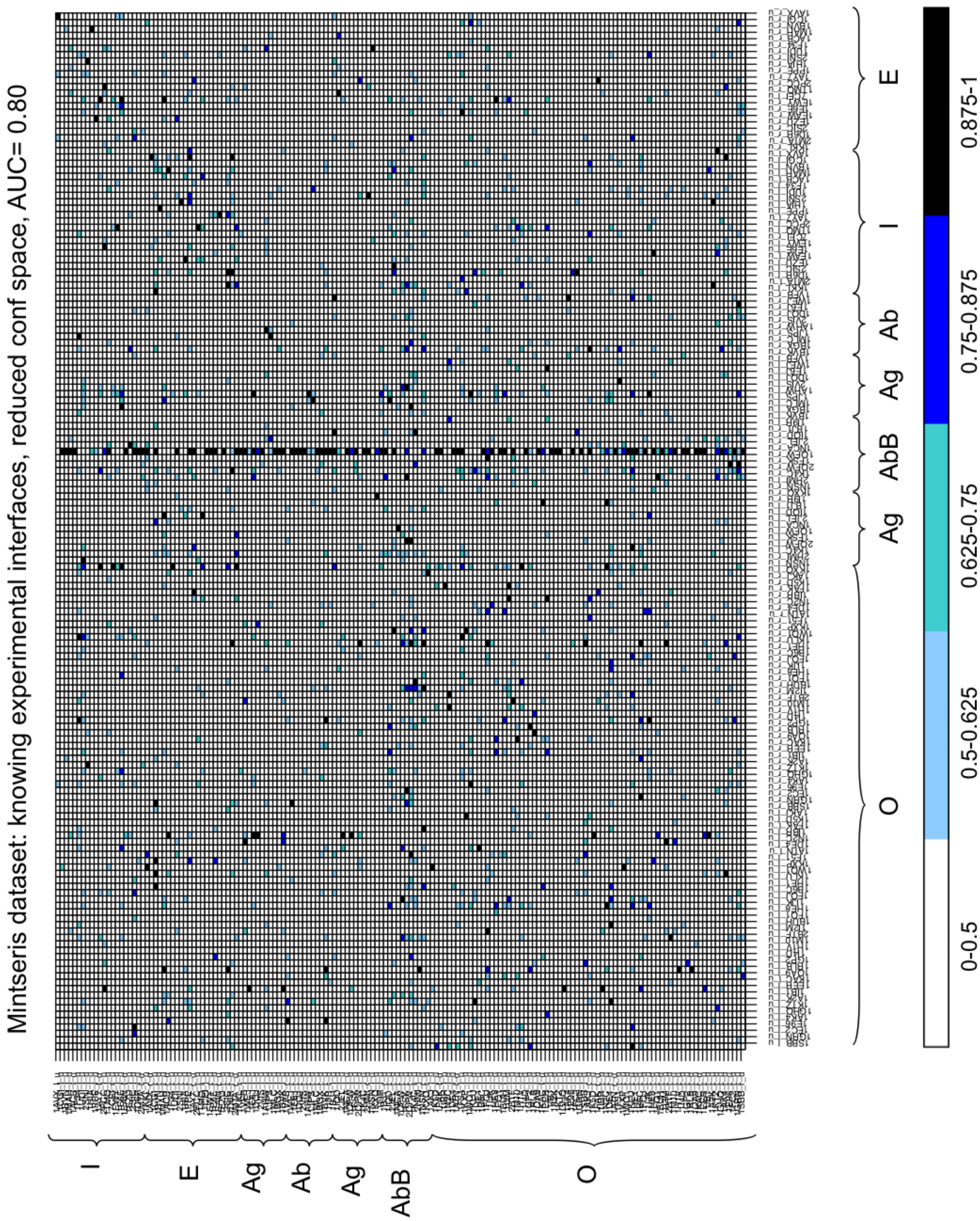

Figure S5: **Normalized Interaction Index (NII) matrix for the complete dataset of 168 proteins restricted to a cone around the receptor**. The matrix is ordered with the experimental complexes lying on the trailing diagonal. Each entry of the matrix corresponds to the NII value computed for the corresponding pair of proteins. High interaction scores (between 0.7 and 1, blue and black in the color scale) indicate a high interaction probability. Interaction scores are computed using knowledge of the experimental interfaces. The plot corresponds to an AUC=0.80.

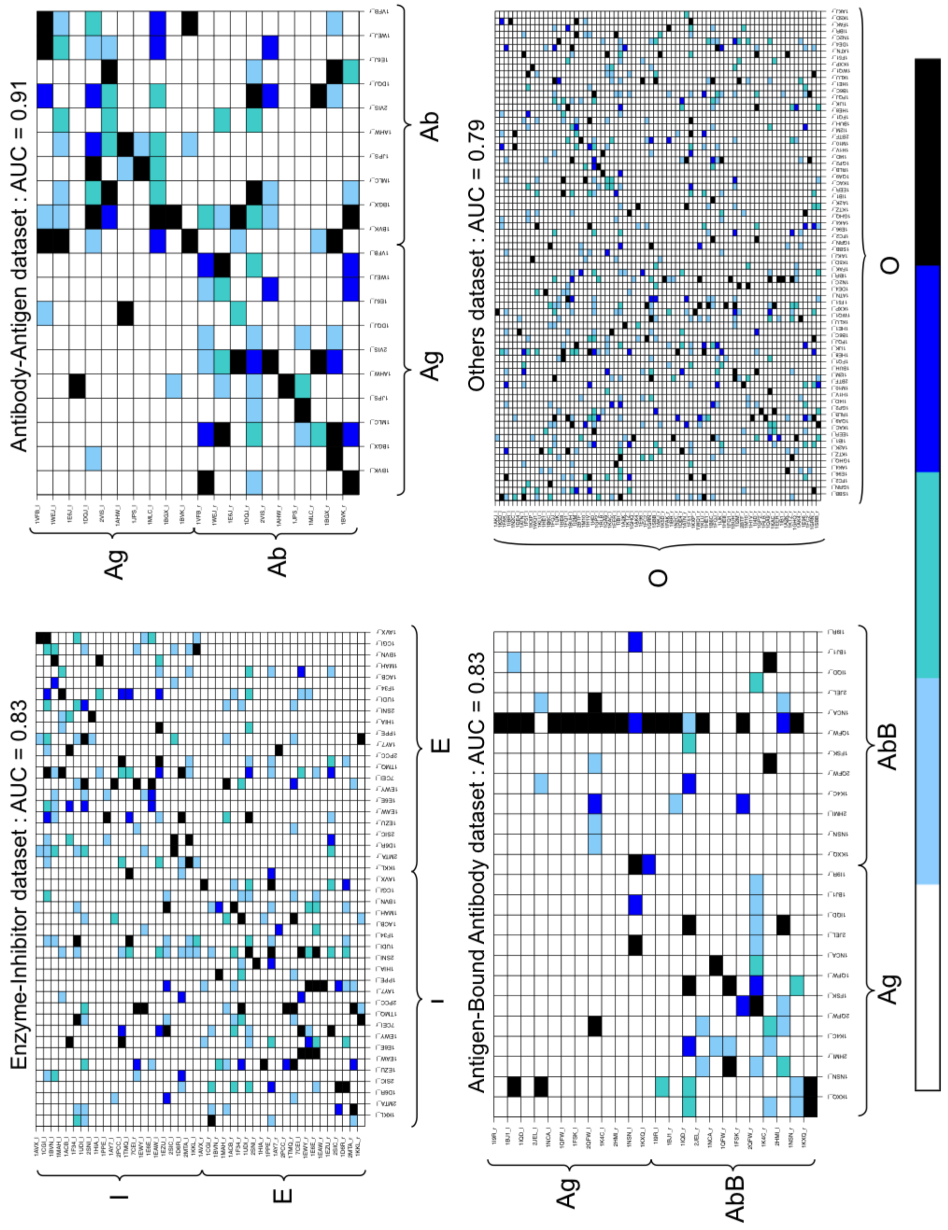

Figure S6: **NII matrix for functional classes of proteins - experimental interfaces computed over the restricted conformational space**. Enzyme-Inhibitor (top left), Antigen-Antibody (top right), Antigen-Bound Antibody (bottom left), Others (bottom right). Matrices are ordered with the experimental complexes lying on the trailing diagonals. Each entry of a matrix corresponds to the NII value computed for the corresponding pair of proteins. High interaction scores (between 0.7 and 1, blue and black in the color scale) indicate a high interaction probability. Interaction scores are computed using knowledge of the experimental interfaces.

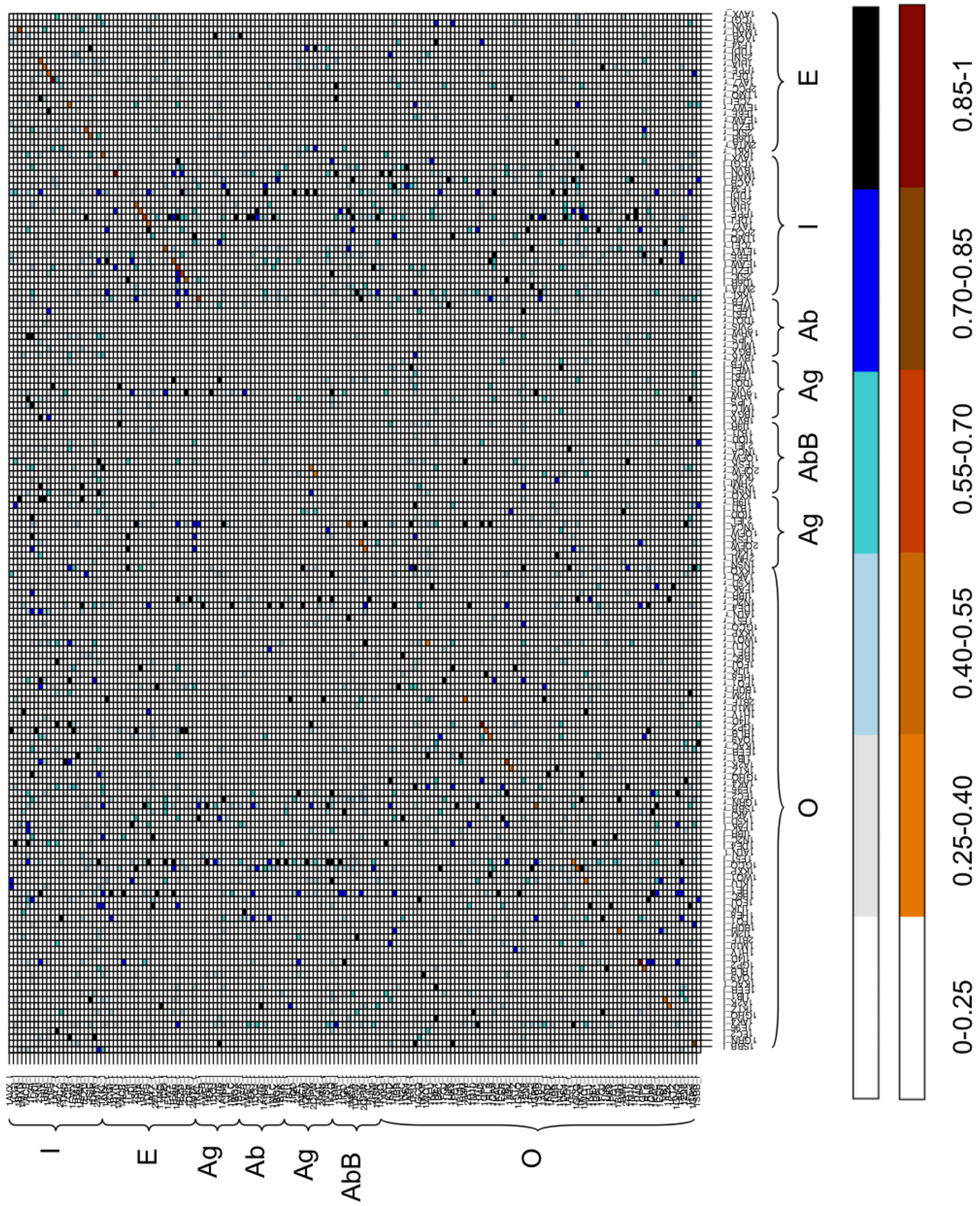

Figure S7: **NII matrix for the Mintseris Database 2.0 - predicted interfaces computed over the full conformational space**. The matrix is ordered with the true complexes lying on the trailing diagonal. Protein structures labeling columns and rows are grouped in functional classes: Enzymes (E), Inhibitors (I), Antibodies (Ab), Antigens (Ag), Bound Antibodies (AbB), Others (O). Each entry of the matrix corresponds to the NII value computed for the corresponding pair of proteins. Two color scales are used for entries in the matrix (blue tones) and for entries in the trailing diagonal (red tones). High interaction scores (between 0.66 and 1, blue and black/dark red and brown) indicate a high interaction probability. Interaction scores are computed using knowledge of the predicted interfaces. The matrix has AUC=0.61.

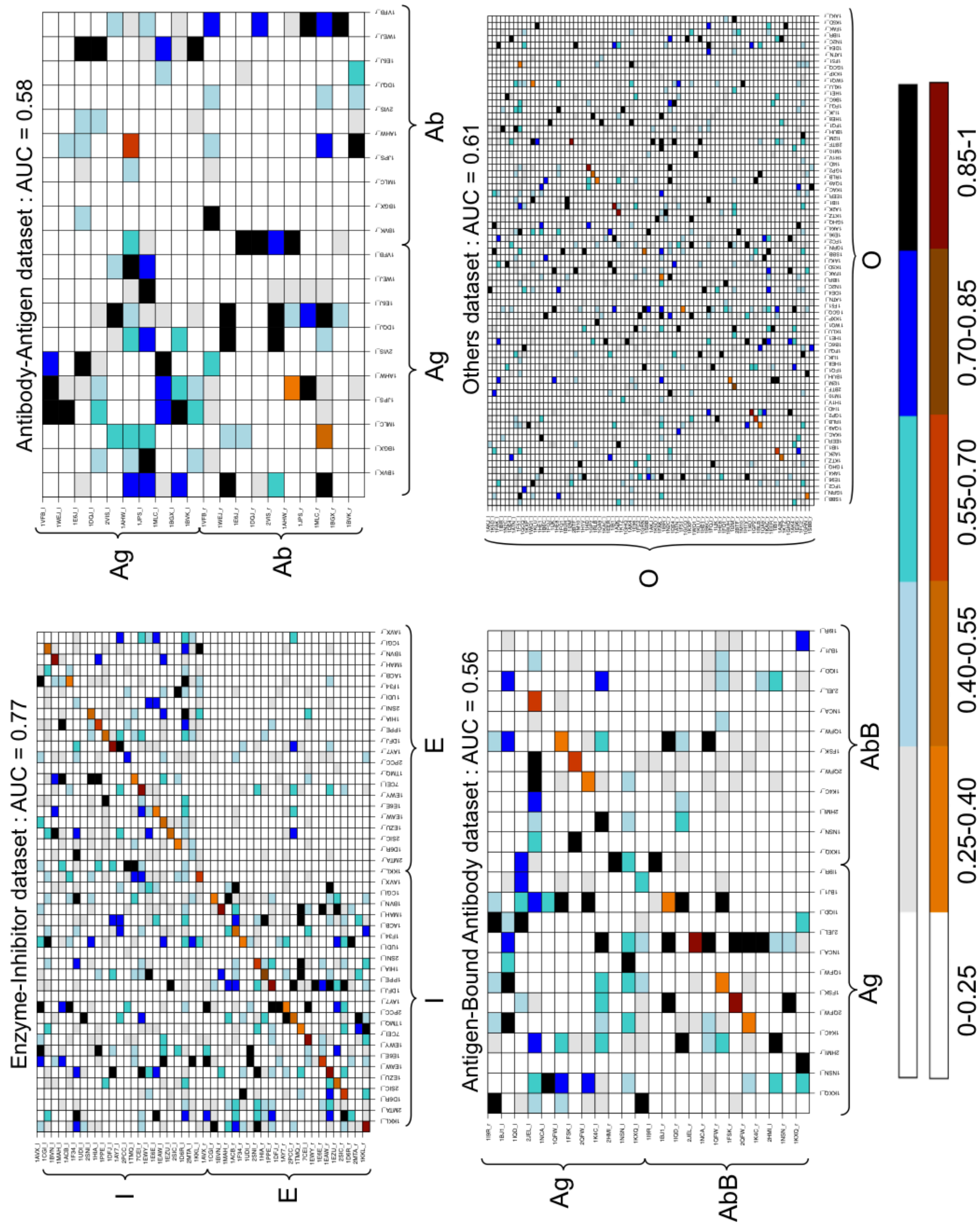

Figure S8: **NII matrices for functional classes of proteins - predicted interfaces computed over the full conformational space**. Enzyme-Inhibitors (EI; top left), Antibody-Antigen (AbAg; top right), Antigen-Bound Antibody (AgAbB; bottom left), Others (O; bottom right). Matrices are ordered with the true complexes lying on the trailing diagonals and, for each matrix, protein structures are grouped in functional classes. Two color scales are used for entries in the matrix (blue tones) and for entries in the trailing diagonal (red tones). Each entry of a matrix corresponds to the NII value computed for the corresponding pair of proteins. High interaction scores (between 0.66 and 1, blue and black/dark red and brown) indicate a high interaction probability. Interaction scores are computed using knowledge of the predicted interfaces.

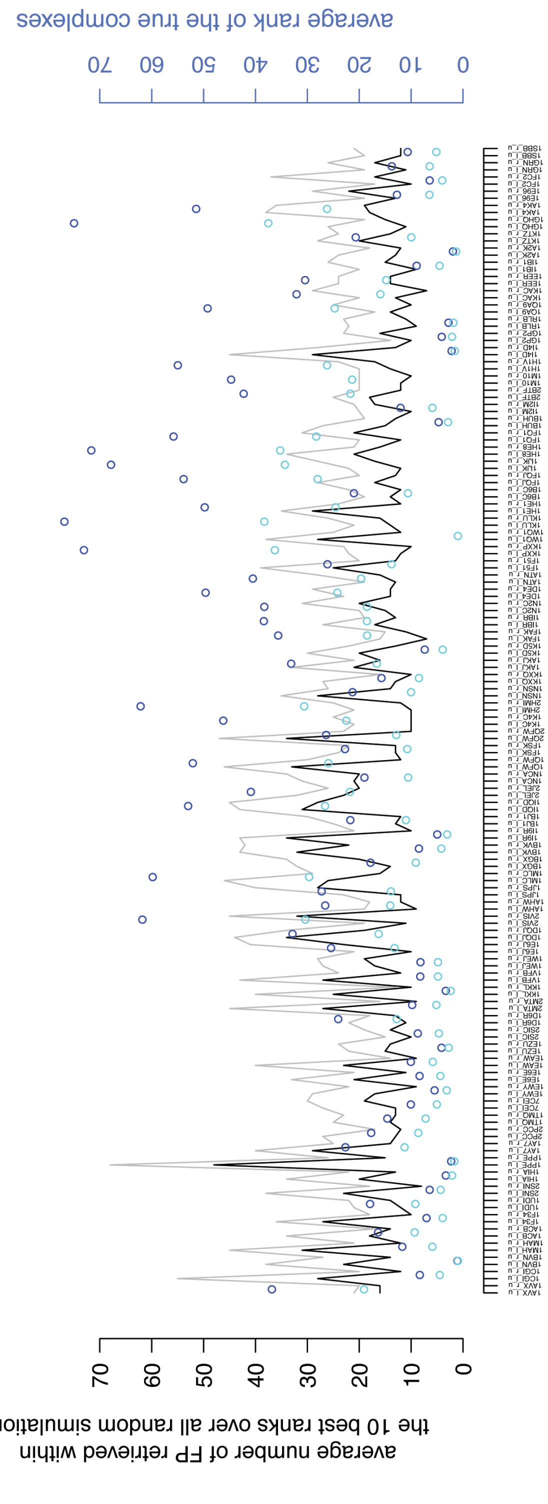

Figure S9: **Average IR for true complexes in the Mintseris Benchmark 2.0 and number of false positives**. The cross docking has been performed on the full conformational space and the IR is computed based on predictions. For each monomer, we plot as false positives (FPs, black curve) the number of partners (excepted the true one) showing an average IR ≤ 10, where the IR is computed over 100 random sets of 20 complexes. The cyan dots indicate the average IR of the true partner over the 100 experiments. The grey curve corresponds to the same experiment run over random sets of 40 complexes and the blue dots to the average IR of the true partners in this second experiment.

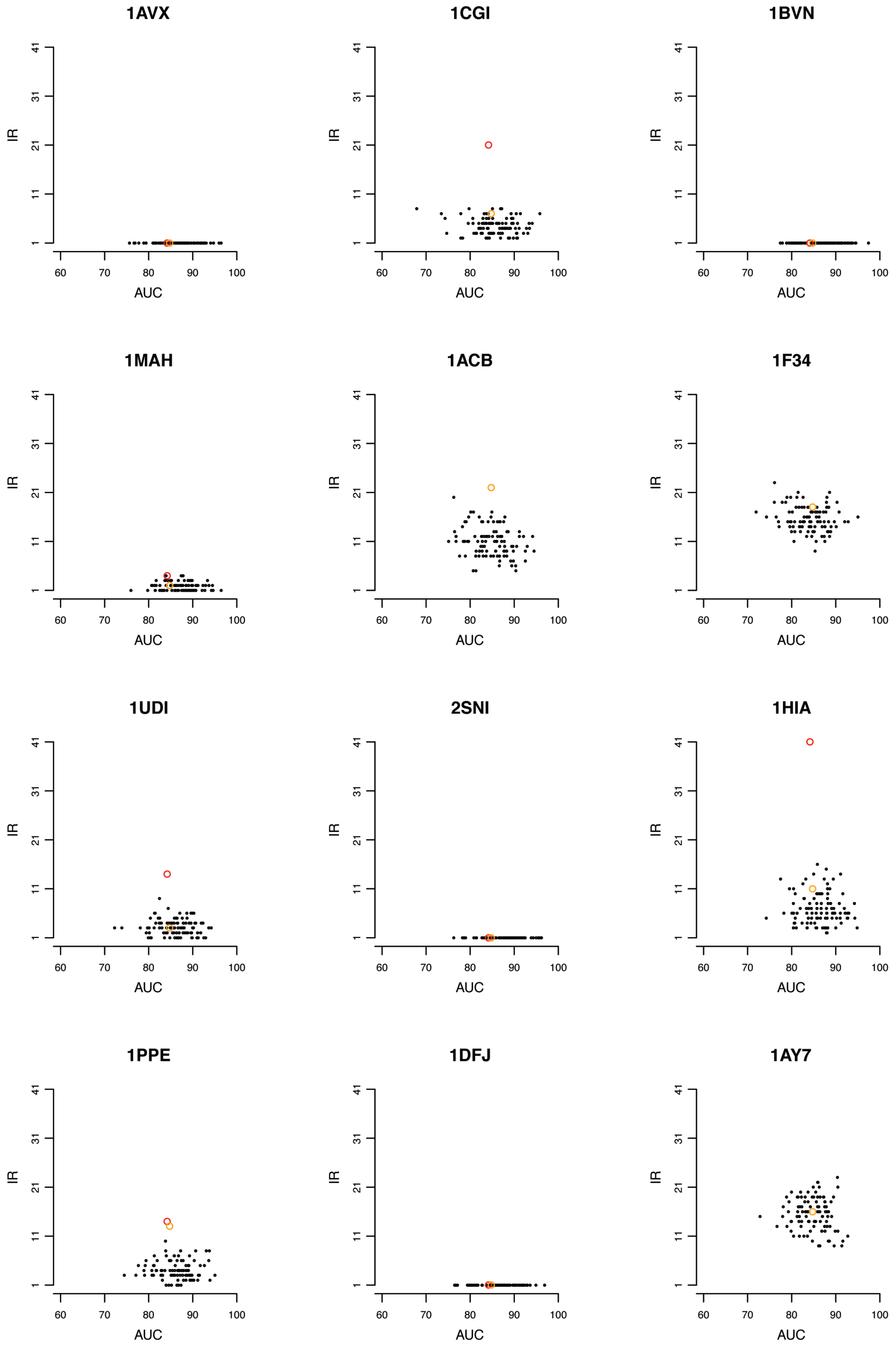

Figure S10: **IR analysis based on experimental interfaces computed over the full conformational space - datasets of 20 complexes randomly chosen in Mintseris Benchmark 2.0**. Each plot corresponds to a complex of the Mintseris Benchmark 2.0. Complexes are organized by functional classes: enzyme-inhibitors (EI, black), antibody-antigenes (AA, dark blue), antibody-bounded antigenes (AbA, light blue), others (0, green). Each colored dot corresponds to the reference complex analyzed with respect to a different random dataset of 40 proteins. Each plot contains 100 black points. The red dot in each plot corresponds to the rank of the complex when evaluated against the 168 proteins of the whole Benchmark Database, and the orange point corresponds to the rank of the complex when evaluated against the set of proteins of the same functional class (EI, AA, AbA, O).

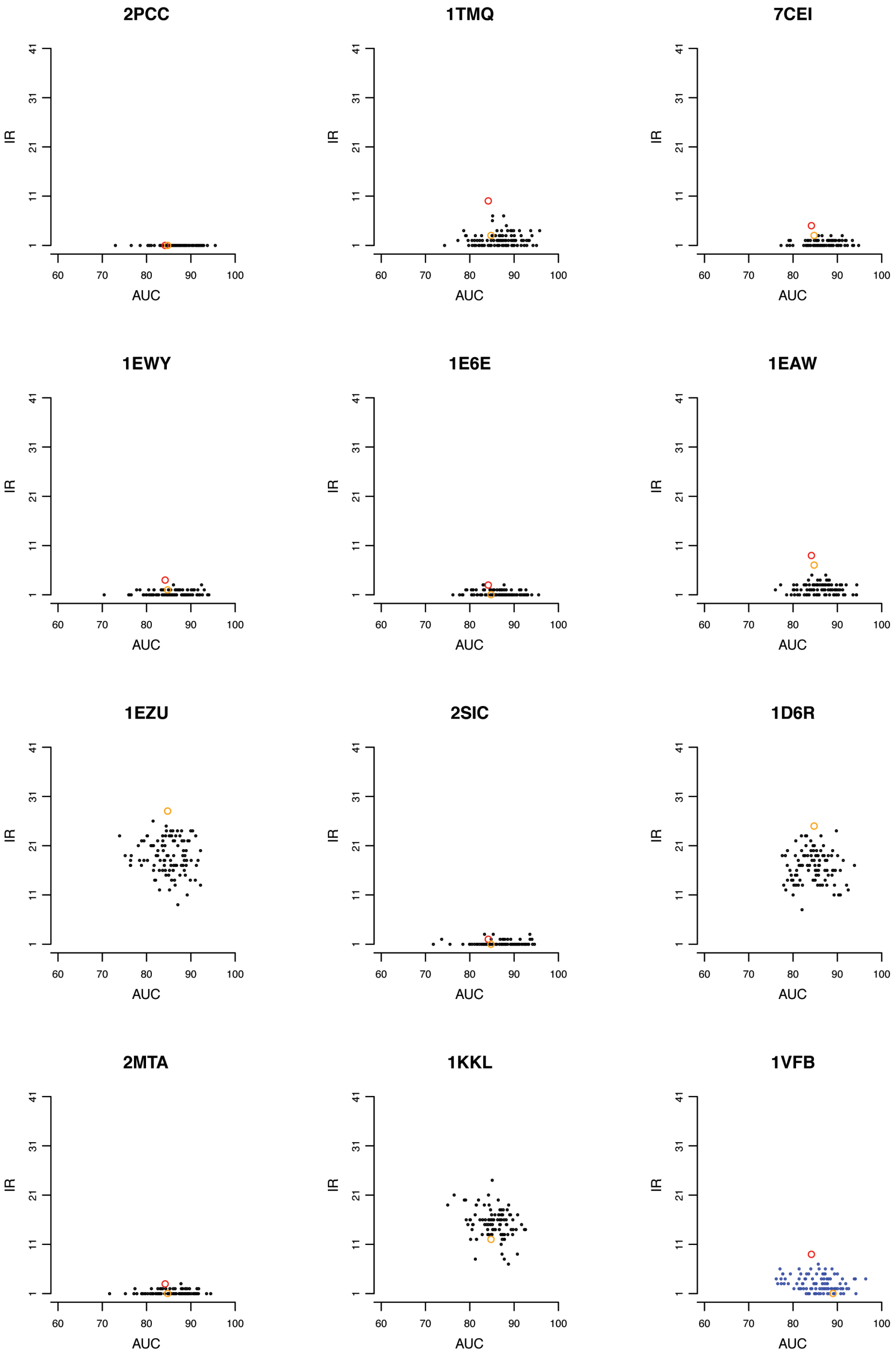

Figure S11: **IR analysis based on experimental interfaces computed over the full conformational space - datasets of 20 complexes randomly chosen in Mintseris Benchmark 2.0**. See legend in Fig. S10.

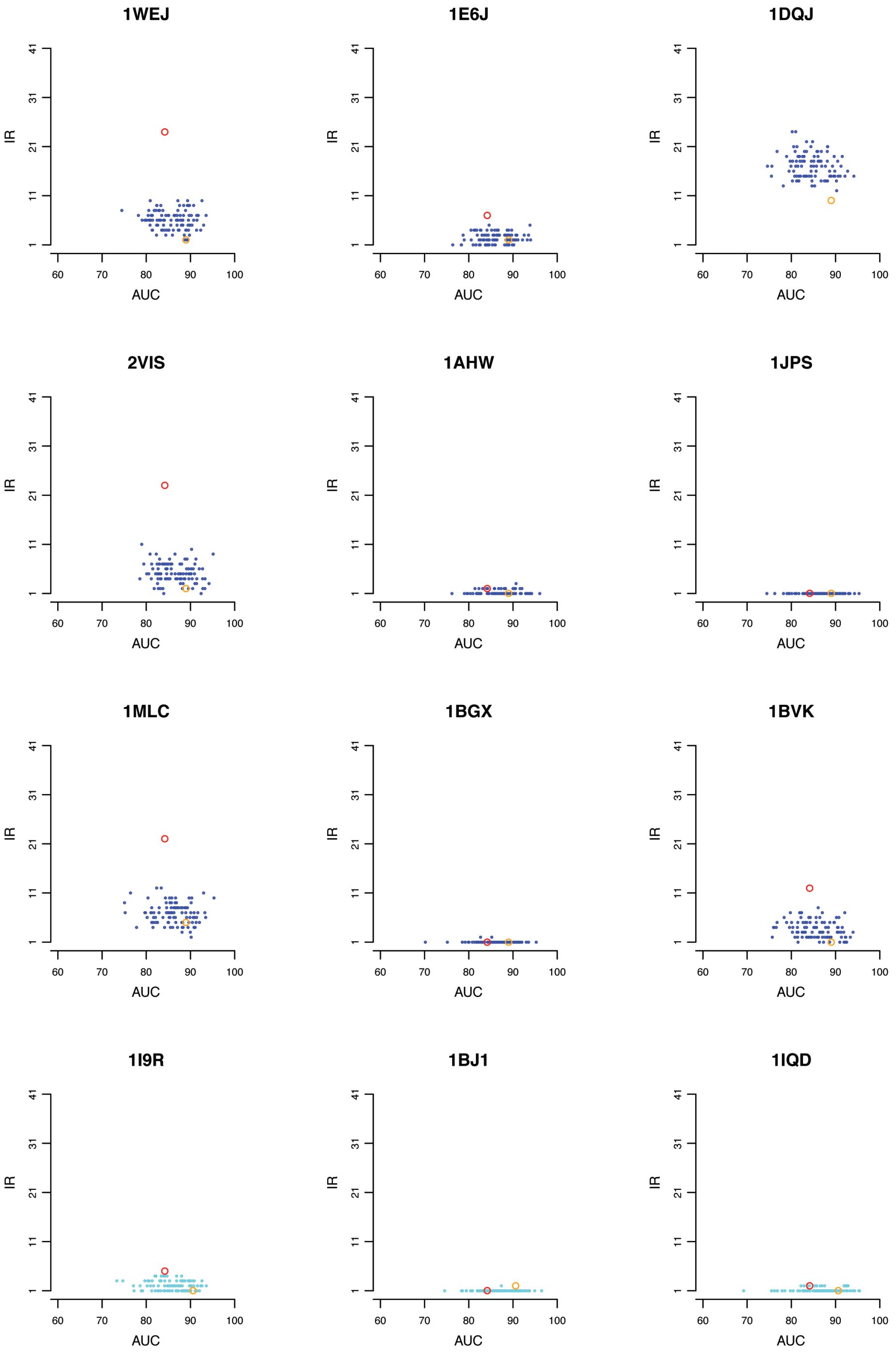

Figure S12: **IR analysis based on experimental interfaces computed over the full conformational space - datasets of 20 complexes randomly chosen in Mintseris Benchmark 2.0**. See legend in Fig. S10.

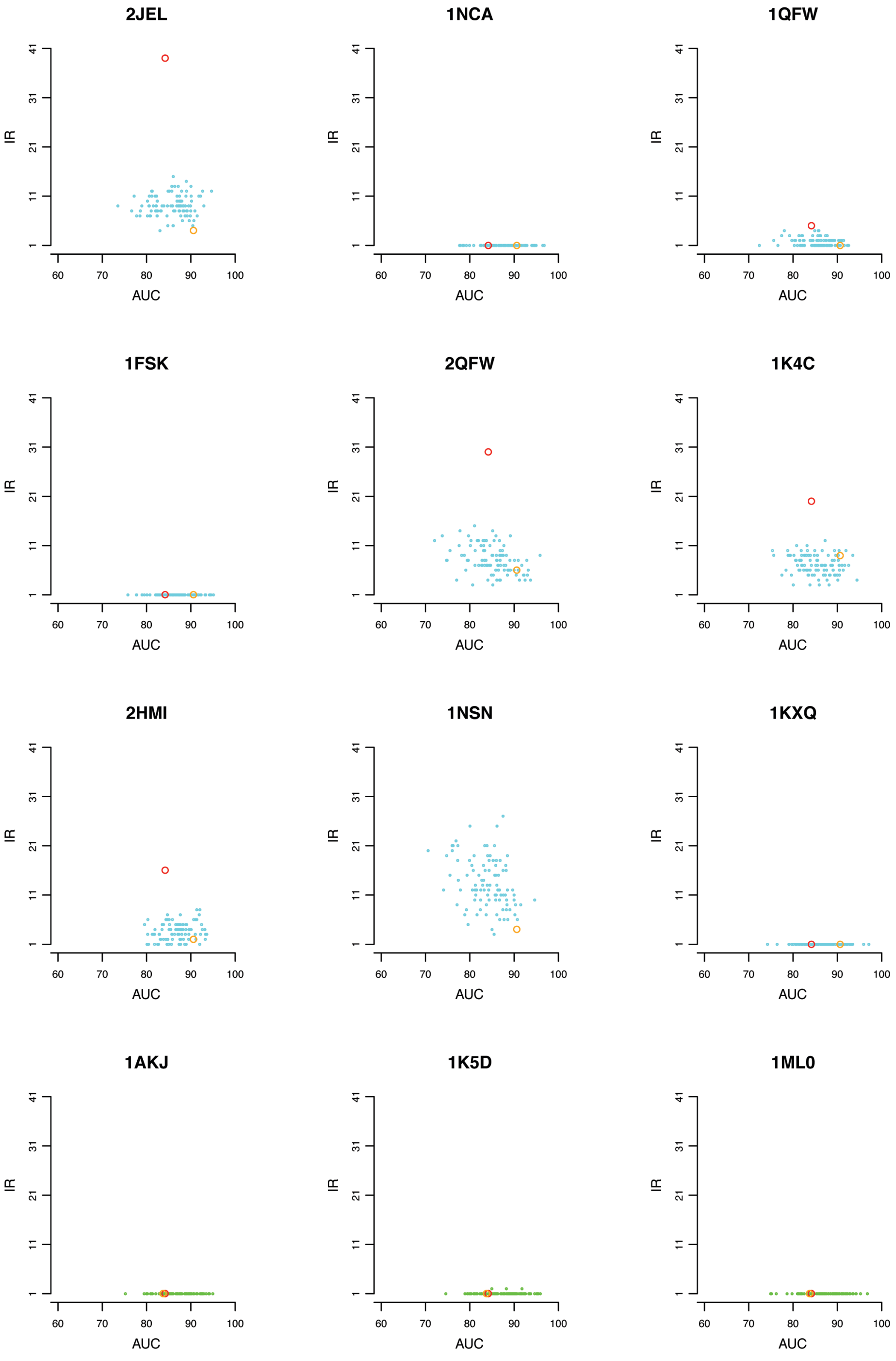

Figure S13: **IR analysis based on experimental interfaces computed over the full conformational space - datasets of 20 complexes randomly chosen in Mintseris Benchmark 2.0**. See legend in Fig. S10.

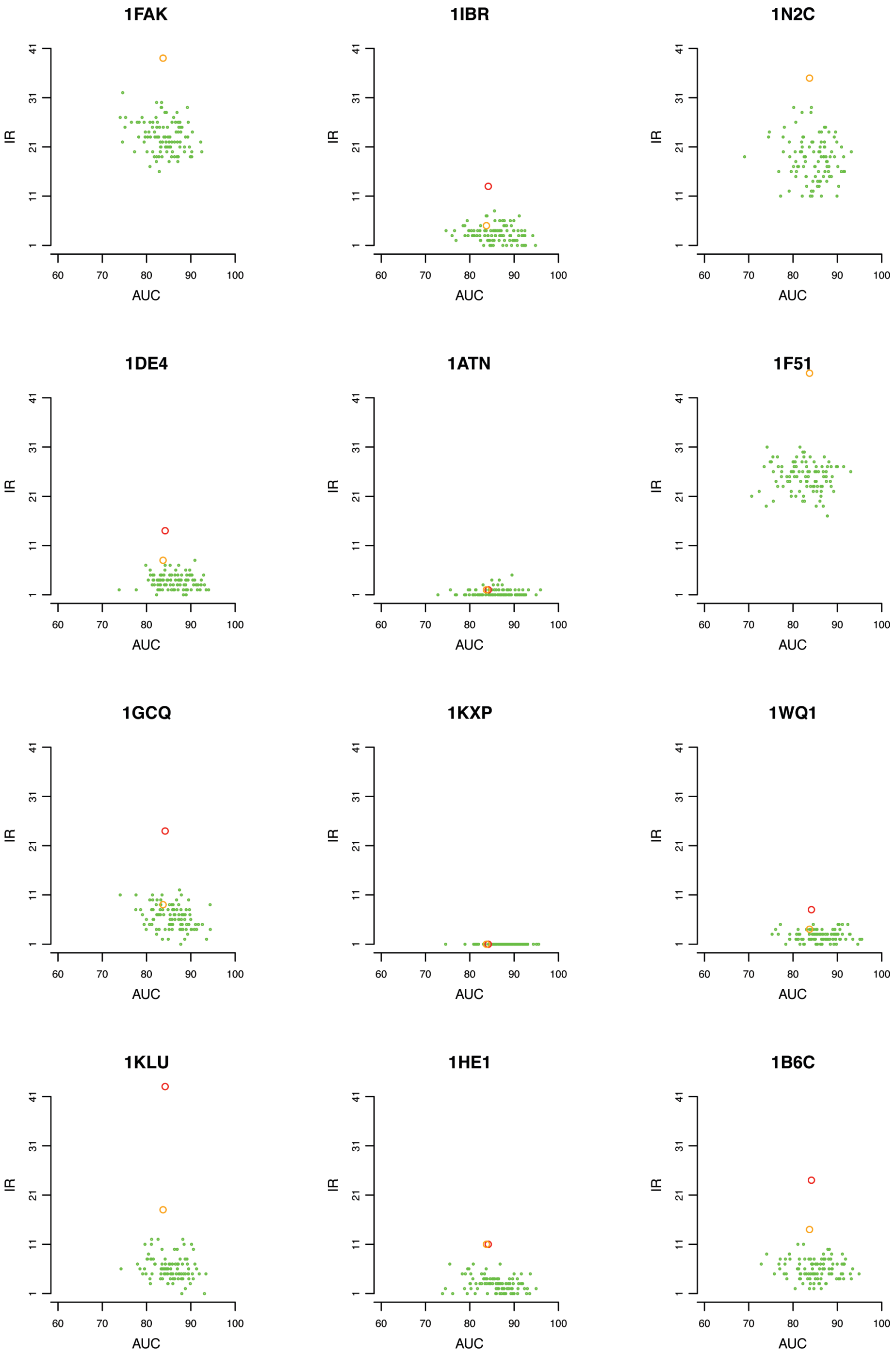

Figure S14: **IR analysis based on experimental interfaces computed over the full conformational space - datasets of 20 complexes randomly chosen in Mintseris Benchmark 2.0**. See legend in Fig. S10.

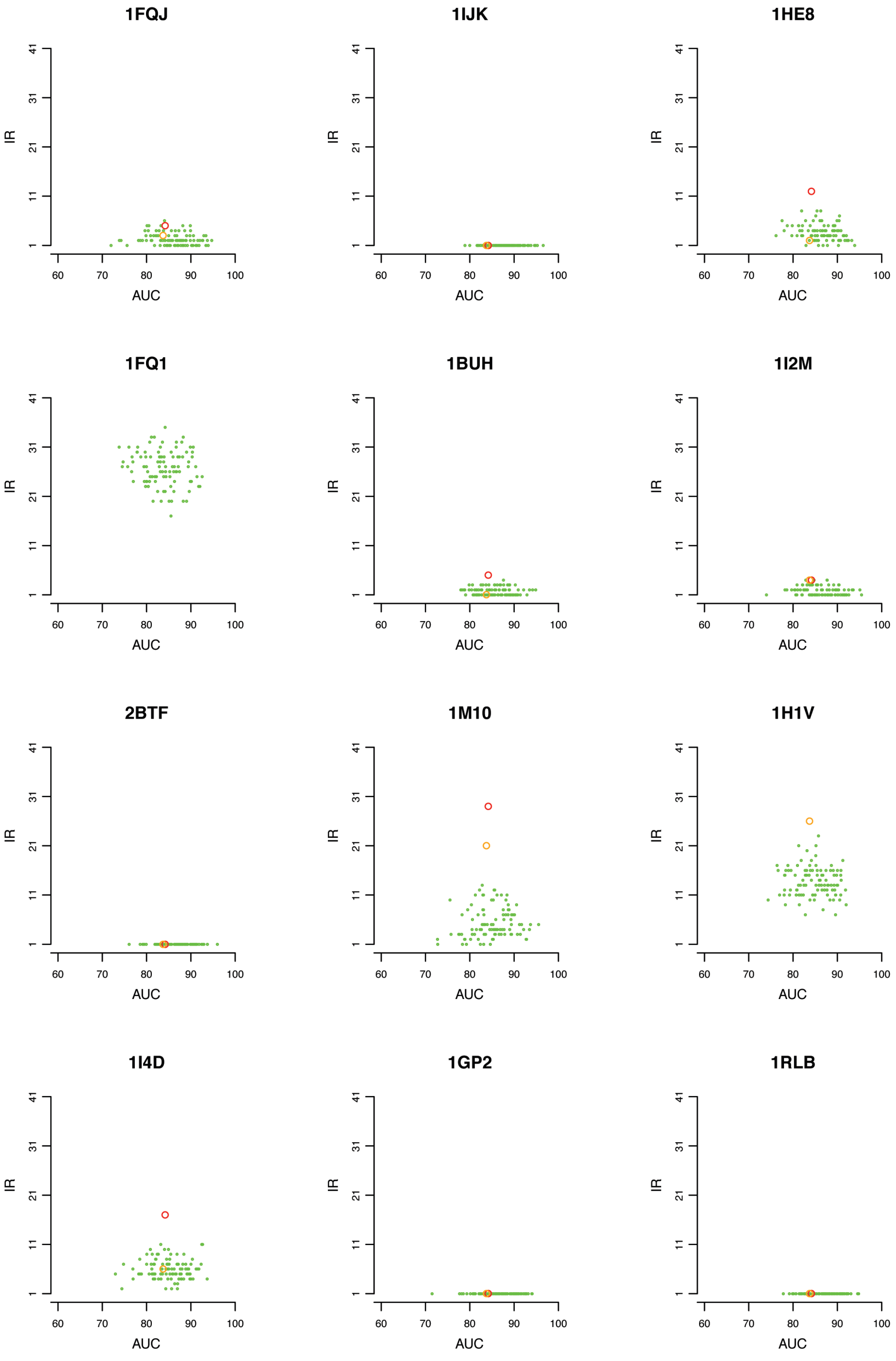

Figure S15: **IR analysis based on experimental interfaces computed over the full conformational space - datasets of 20 complexes randomly chosen in Mintseris Benchmark 2.0**. See legend in Fig. S10.

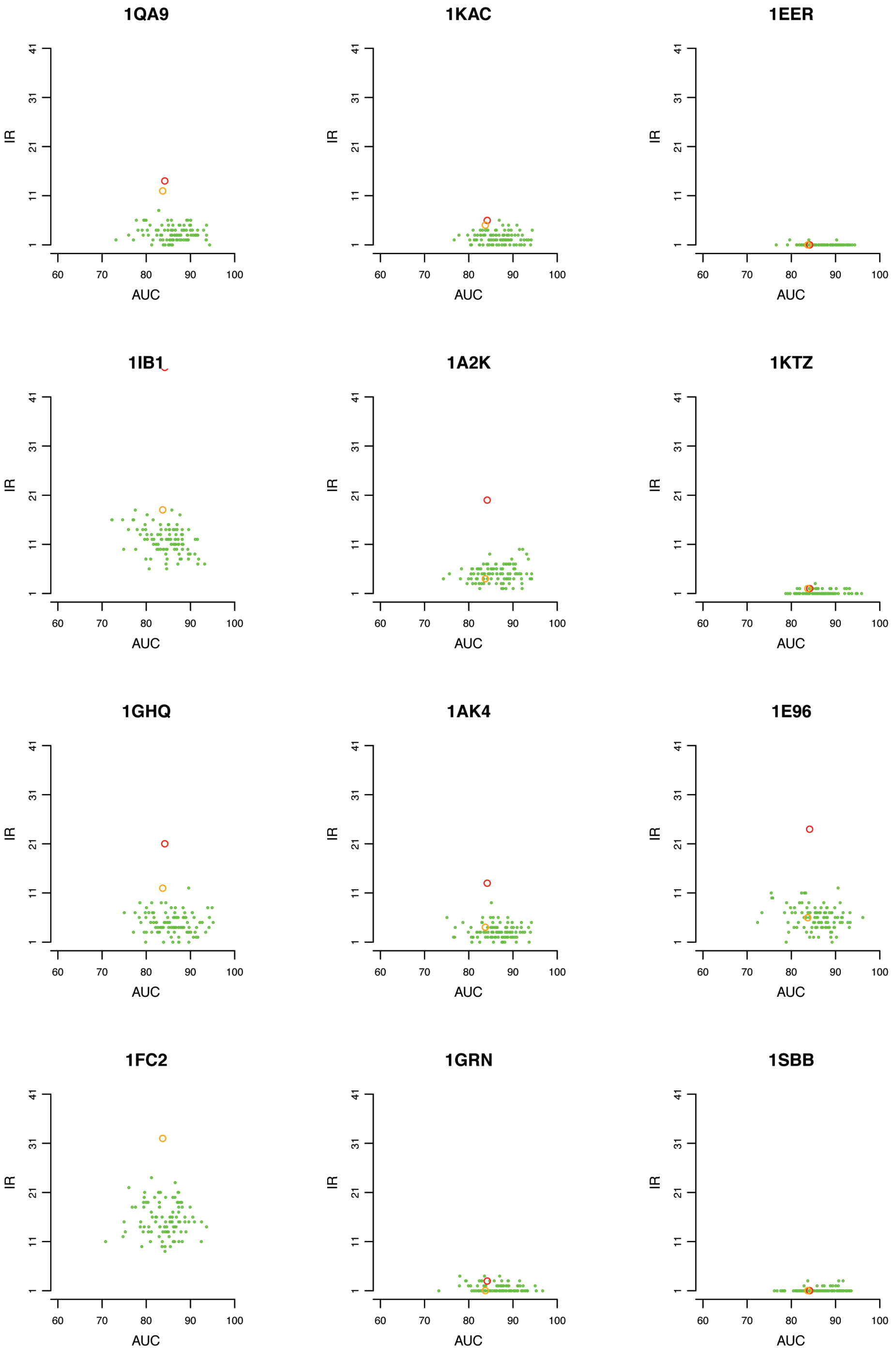

Figure S16: **IR analysis based on experimental interfaces computed over the full conformational space - datasets of 20 complexes randomly chosen in Mintseris Benchmark 2.0**. See legend in Fig. S10.

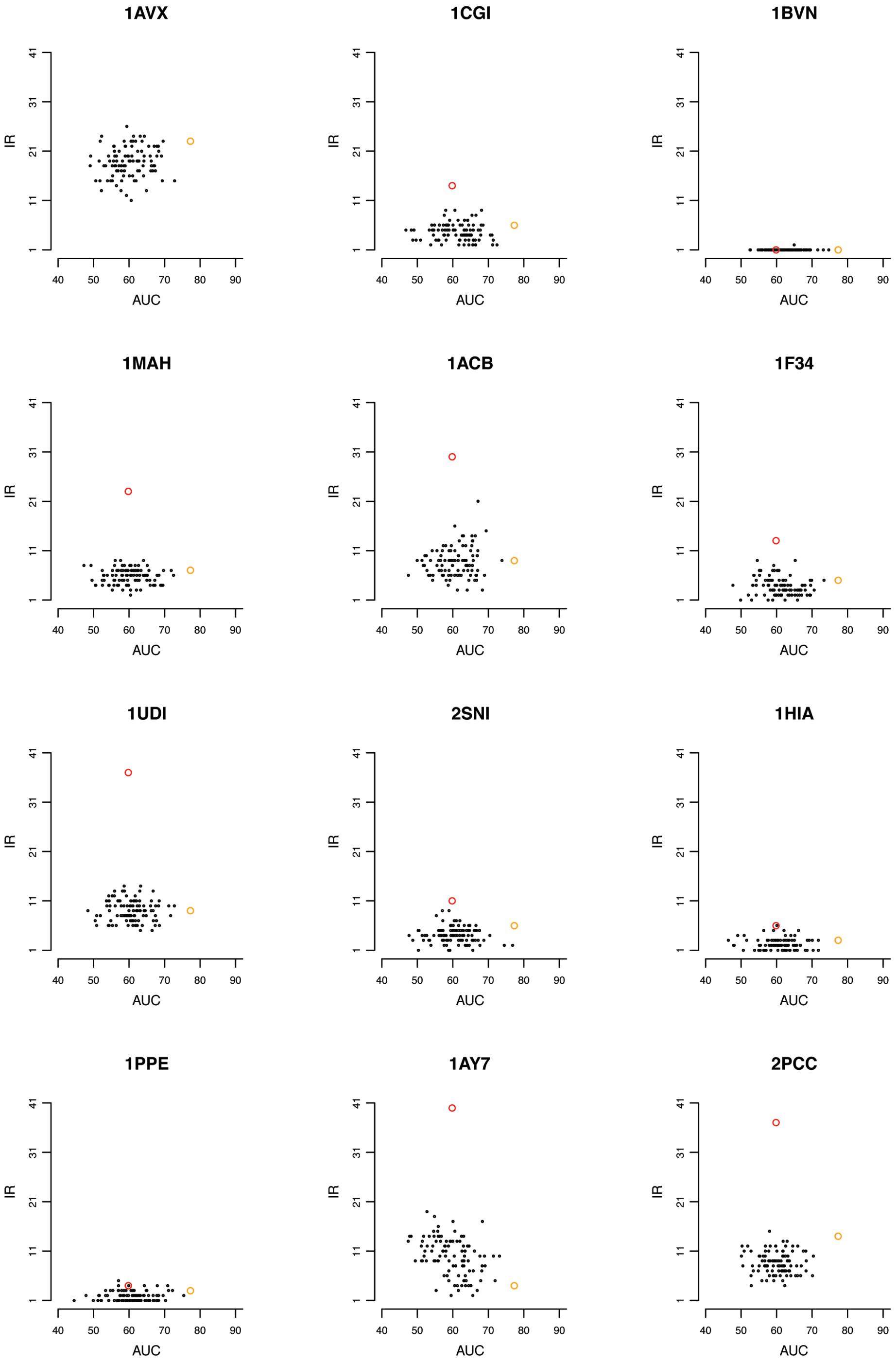

Figure S17: **IR analysis based on predicted interfaces computed over the full conformational space - datasets of 20 complexes randomly chosen in Mintseris Benchmark 2.0**. Each plot corresponds to a complex of the Mintseris Benchmark 2.0. Complexes are organized by functional classes: enzyme-inhibitors (EI, black), antibody-antigenes (AA, dark blue), antibody-bounded antigenes (AbA, light blue), others (0, green). Each colored dot corresponds to the reference complex analyzed with respect to a different random dataset of 40 proteins. Each plot contains 100 black points. The red dot in each plot corresponds to the rank of the complex when evaluated against the 168 proteins of the whole Benchmark database, and the orange point corresponds to the rank of the complex when evaluated against the set of proteins of the same functional class (EI, AA, AbA, O).

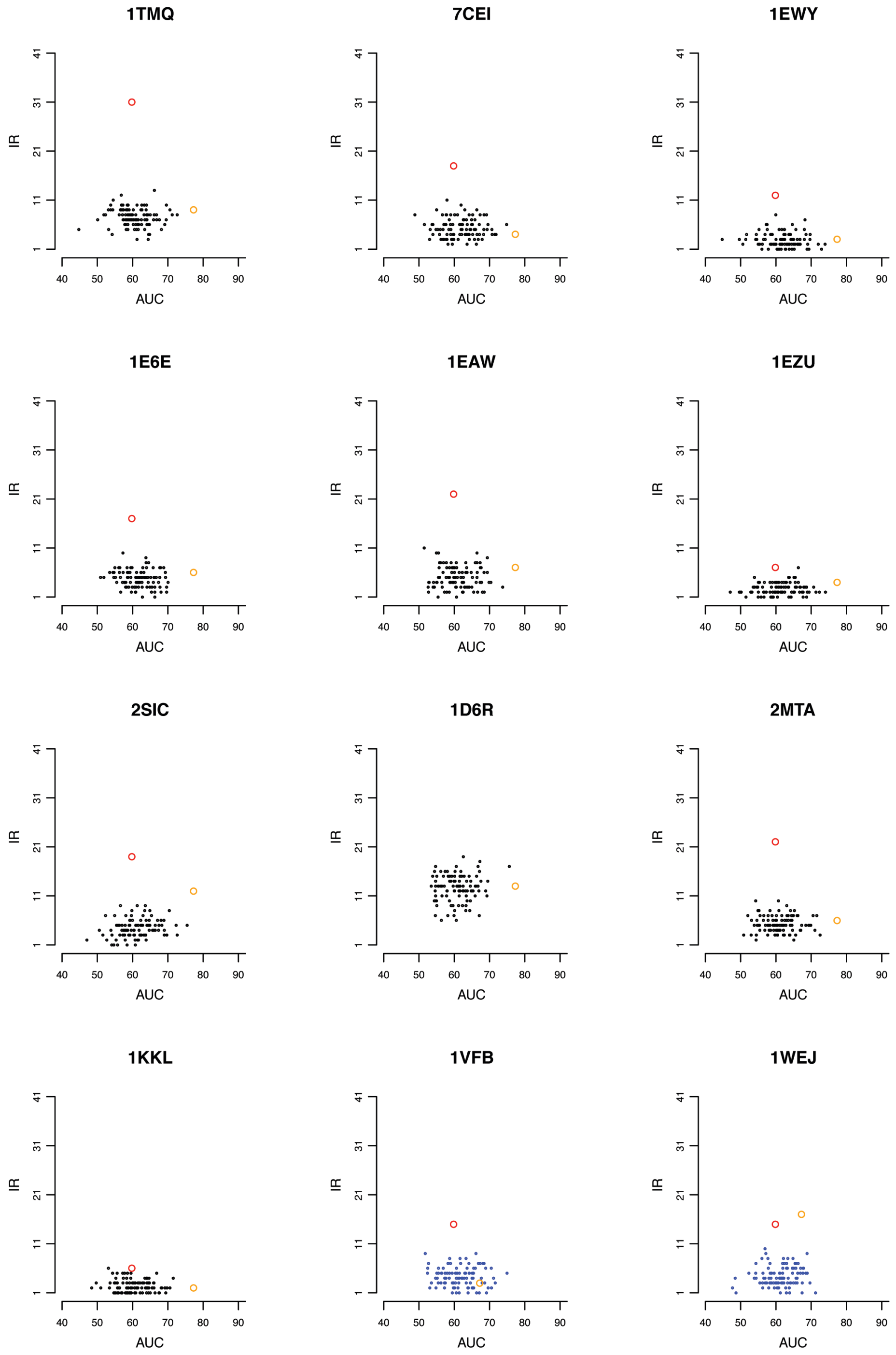

Figure S18: **IR analysis based on predicted interfaces computed over the full conformational space - datasets of 20 complexes randomly chosen in Mintseris Benchmark 2.0**. See legend in Fig. S17.

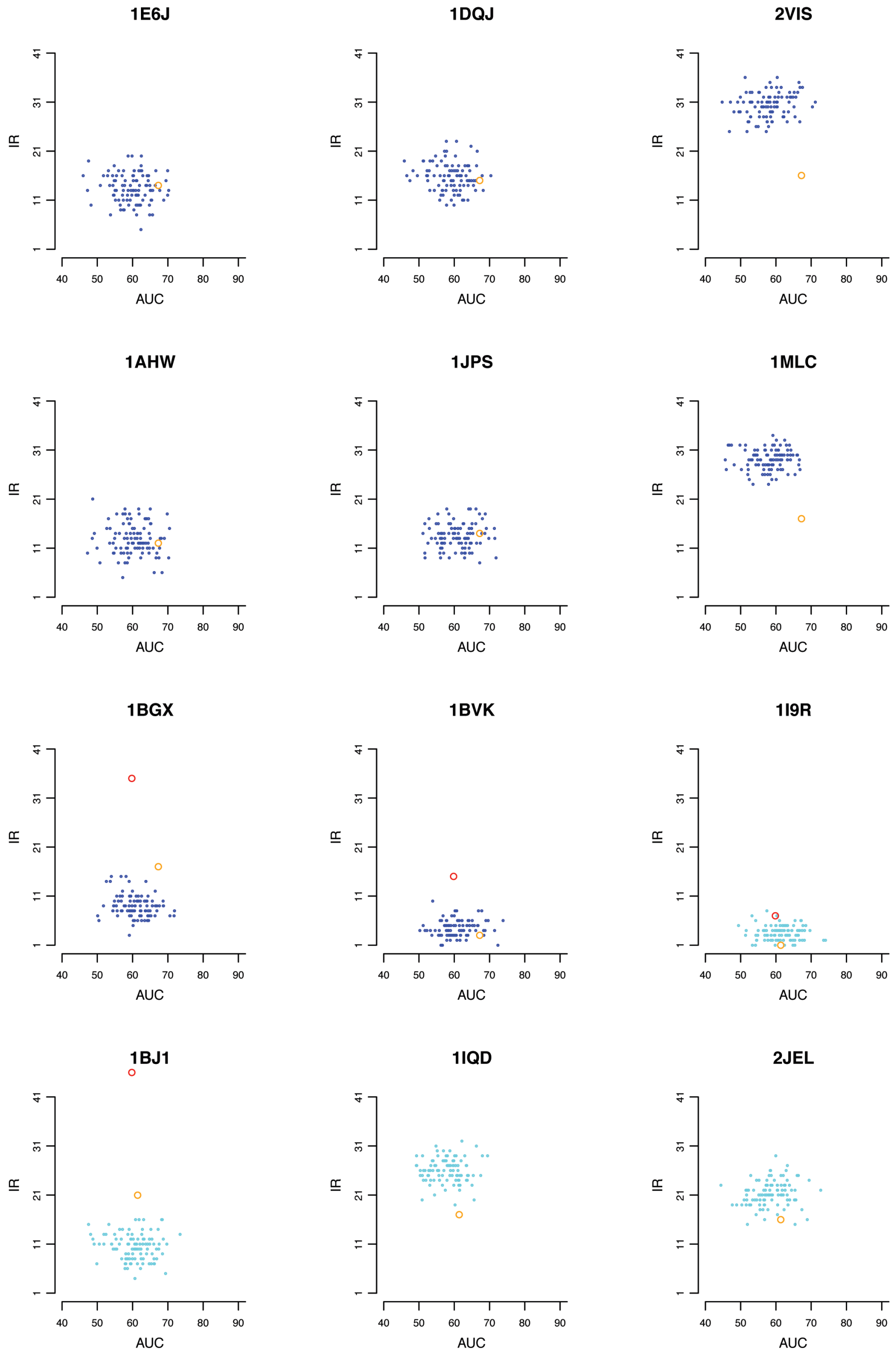

Figure S19: **IR analysis based on predicted interfaces computed over the full conformational space - datasets of 20 complexes randomly chosen in Mintseris Benchmark 2.0**. See legend in Fig. S17.

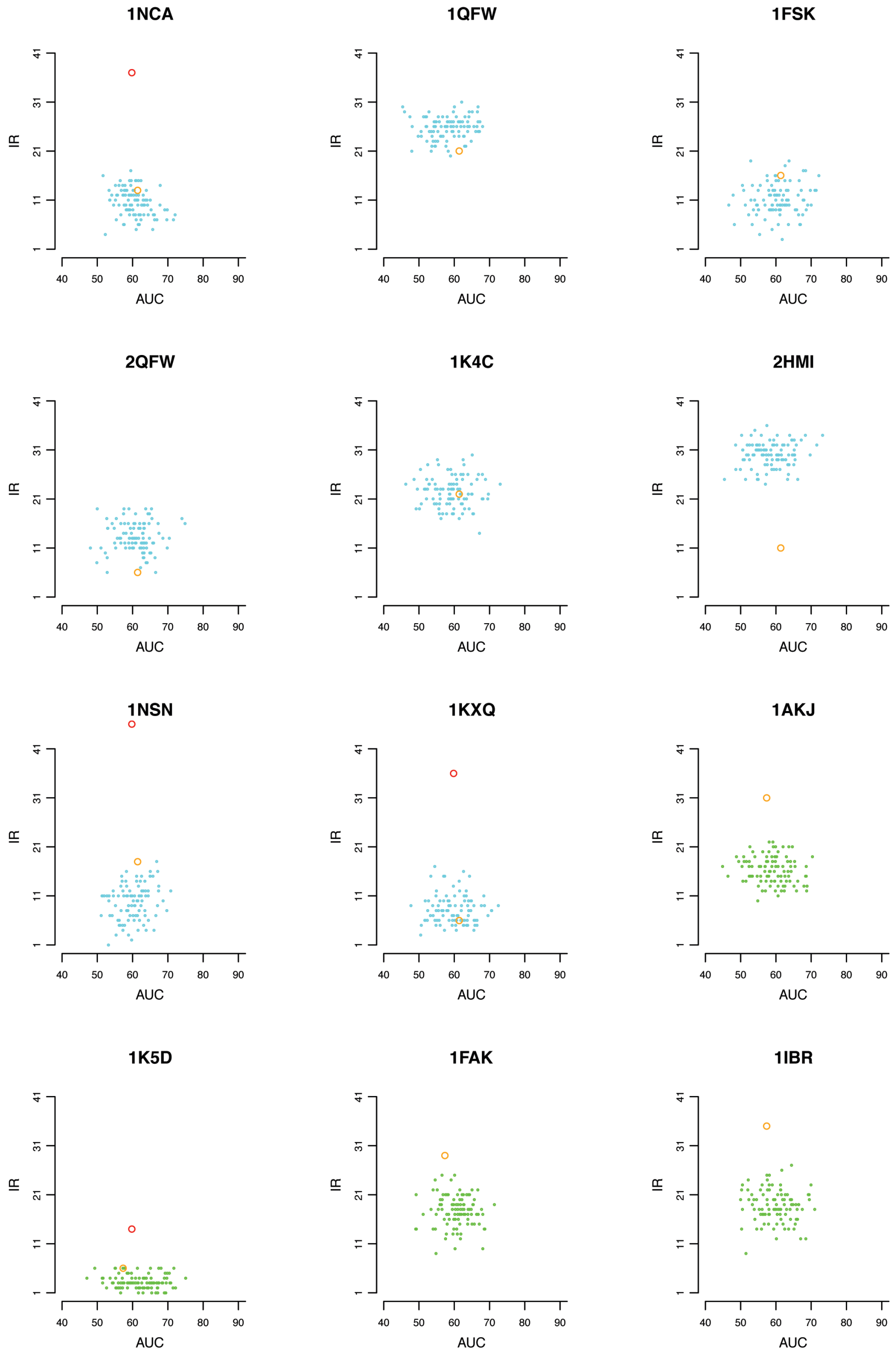

Figure S20: **IR analysis based on predicted interfaces computed over the full conformational space - datasets of 20 complexes randomly chosen in Mintseris Benchmark 2.0**. See legend in Fig. S17.

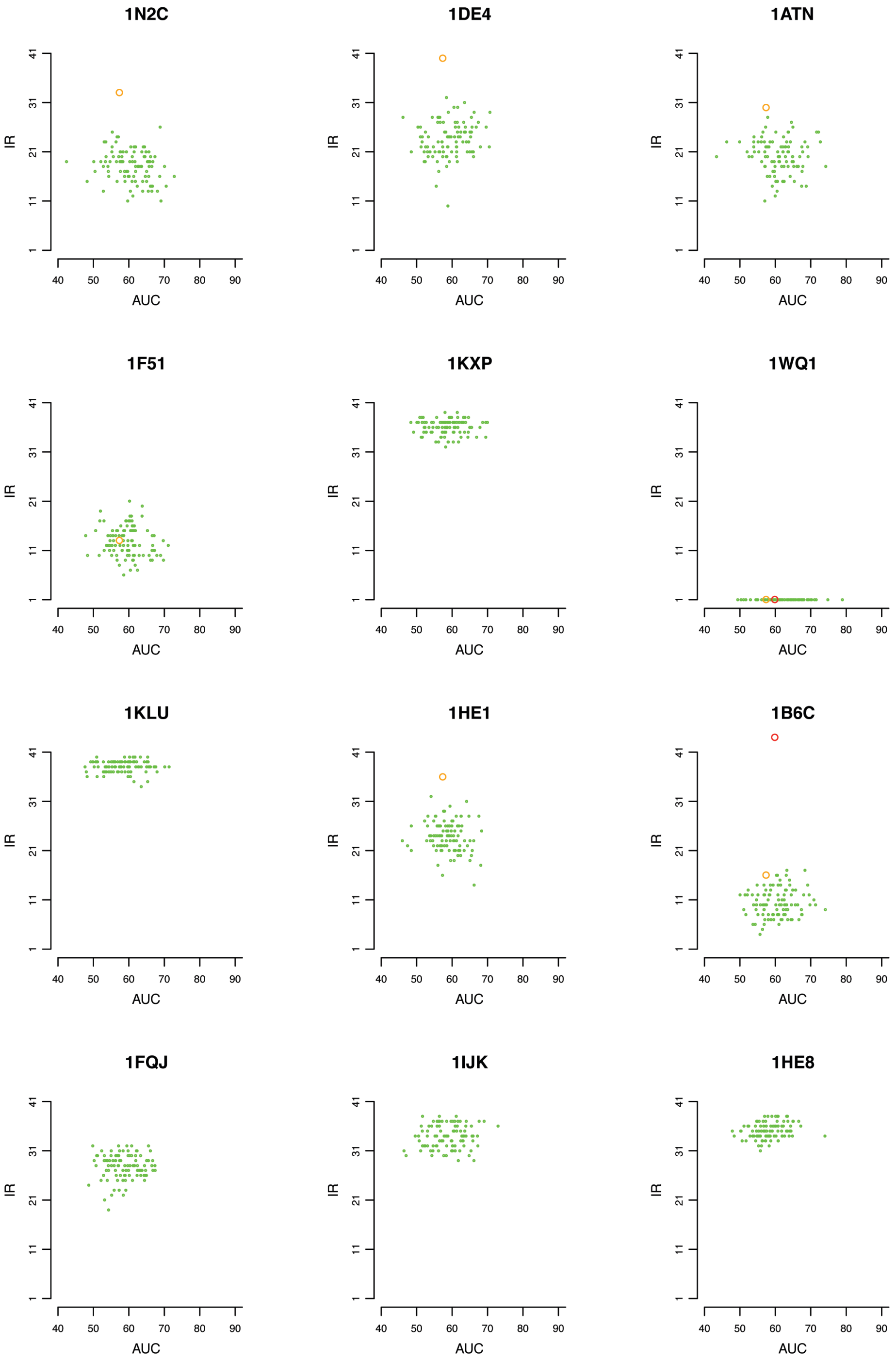

Figure S21: **IR analysis based on predicted interfaces computed over the full conformational space - datasets of 20 complexes randomly chosen in Mintseris Benchmark 2.0**. See legend in Fig. S17.

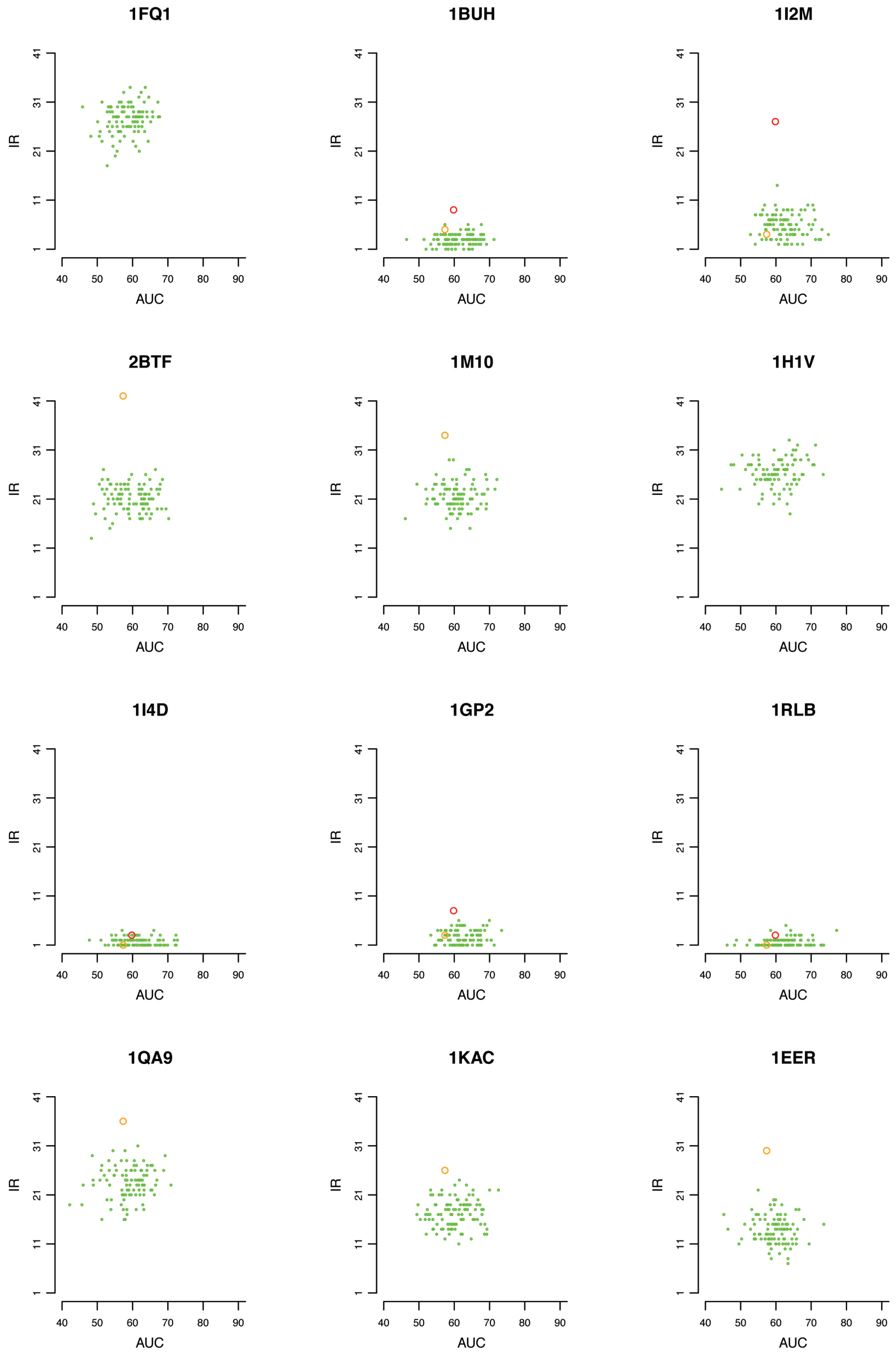

Figure S22: **IR analysis based on predicted interfaces computed over the full conformational space - datasets of 20 complexes randomly chosen in Mintseris Benchmark 2.0**. See legend in Fig. S17.

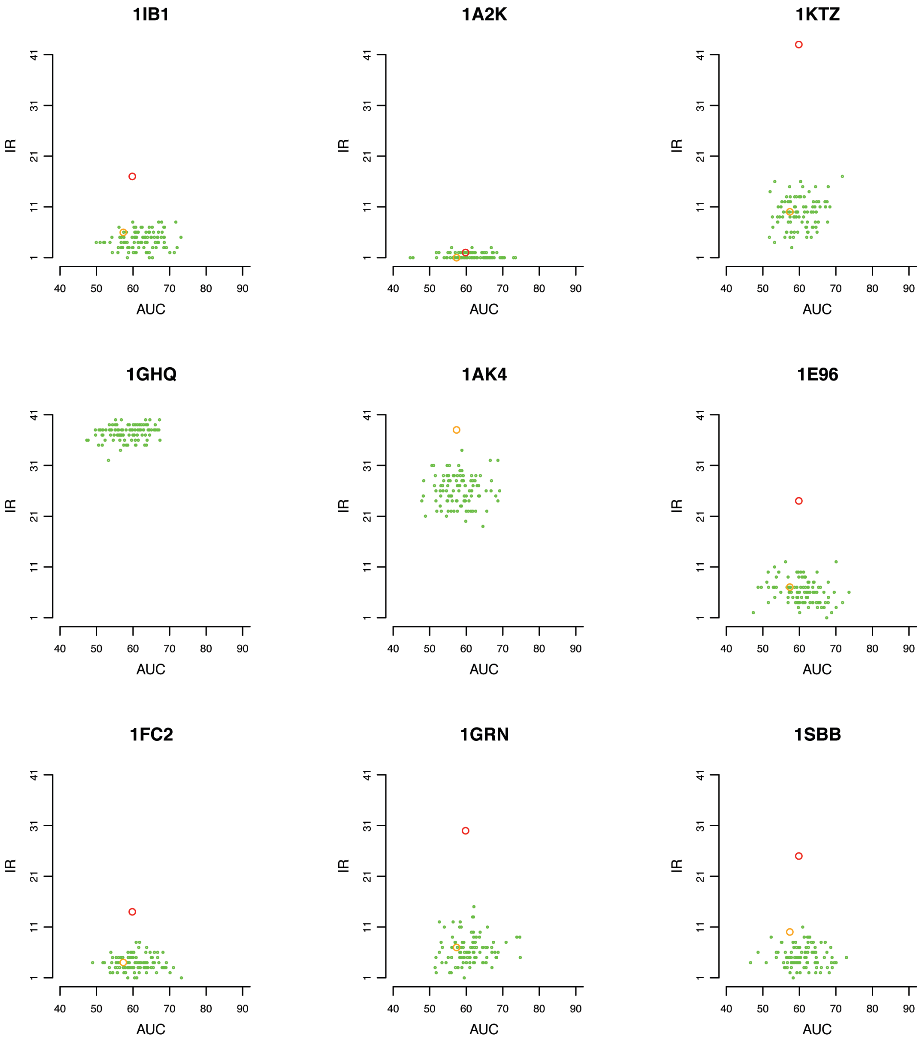

Figure S23: **IR analysis based on predicted interfaces computed over the full conformational space - datasets of 20 complexes randomly chosen in Mintseris Benchmark 2.0**. See legend in Fig. S17.

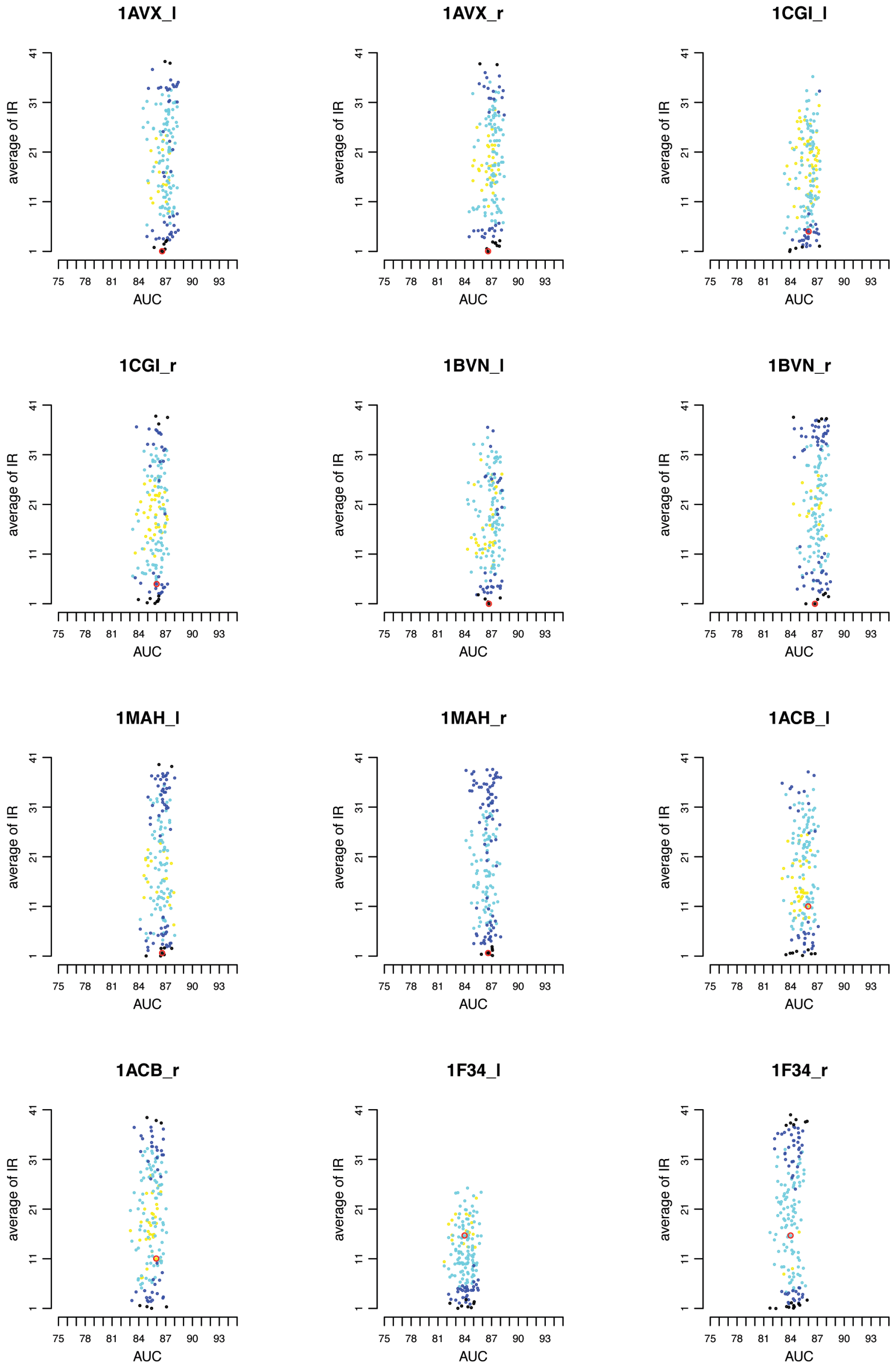

Figure S24: **IR analysis of false complexes against the true one, based on experimental interfaces and the full exploration of the conformational space**. Each monomer of the Mintseris Benchmark 2.0 is coupled with one of the 168 proteins (including the monomer itself) of the dataset forming either a false (167 cases) or the true complex. For each complex, we computed the corresponding average IR and average AUC over 100 random sets of 40 proteins. These values are reported as a point in a plot. Each plot contains 168 points. The red circle in each plot corresponds to the values of the true complex. Dots are colored in a scale from black, blue, cyan, yellow. A color corresponds to the value of the standard deviation σ of the distribution of 100 IRs computed for a complex: black if $\sigma \leq 1$, blue if $\sigma \leq 2$, cyan if $\sigma \leq 3$ and yellow otherwise (i.e. $\sigma > 3$).

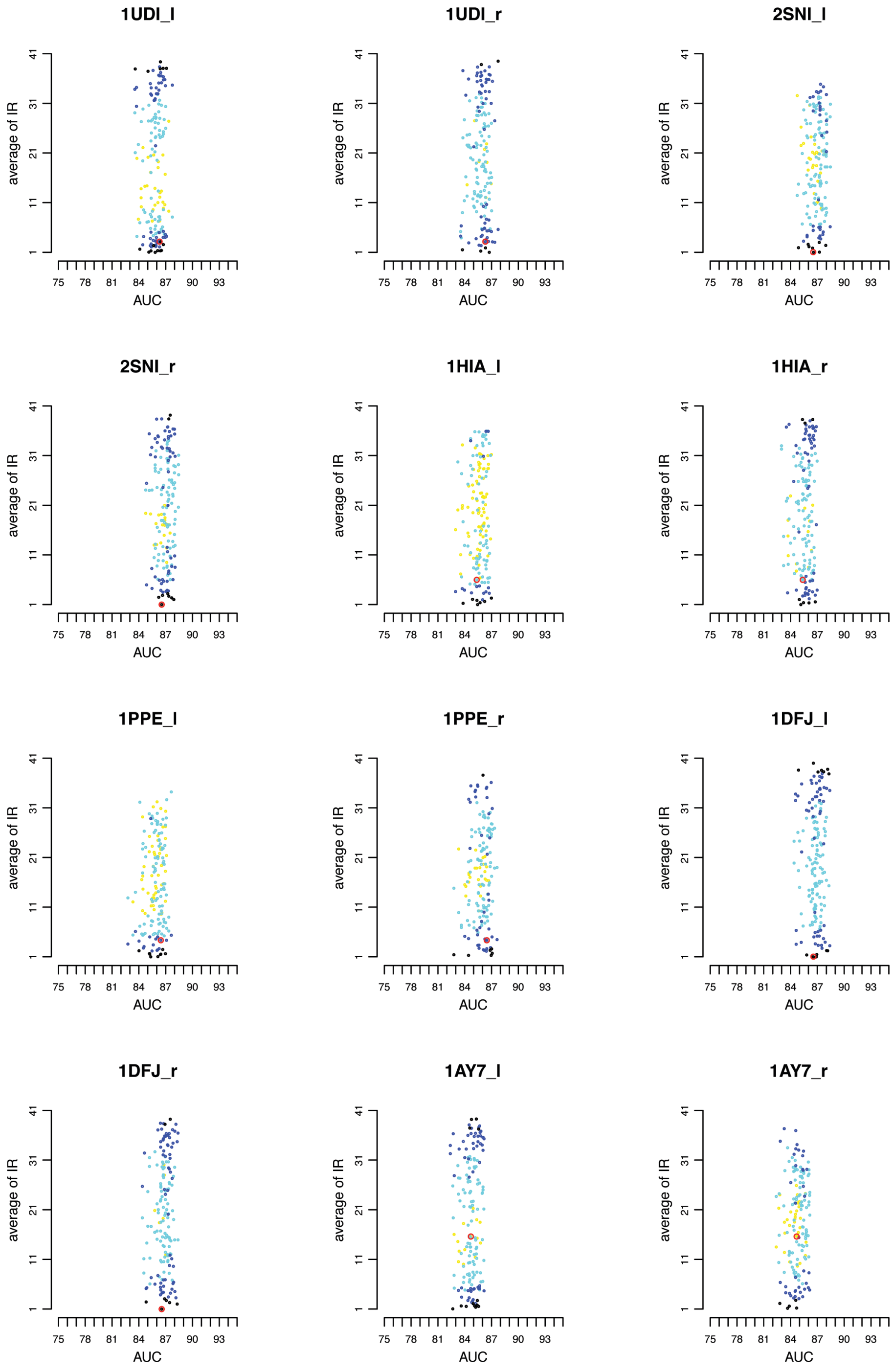

Figure S25: **IR analysis of false complexes against the true one, based on experimental interfaces computed over the full conformational space**. See legend in Fig. S24.

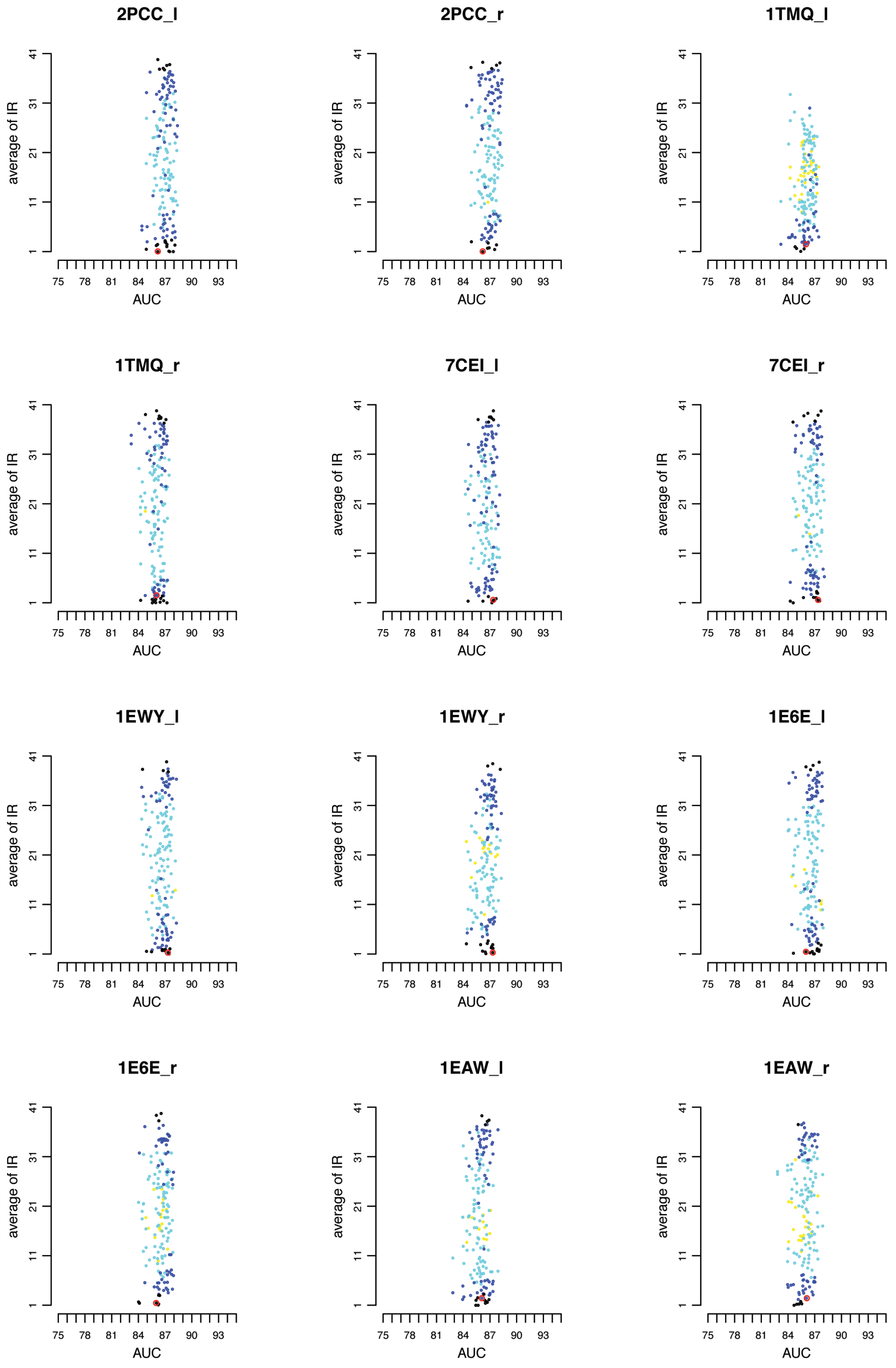

Figure S26: **IR analysis of false complexes against the true one, based on experimental interfaces computed over the full conformational space**. See legend in Fig. S24.

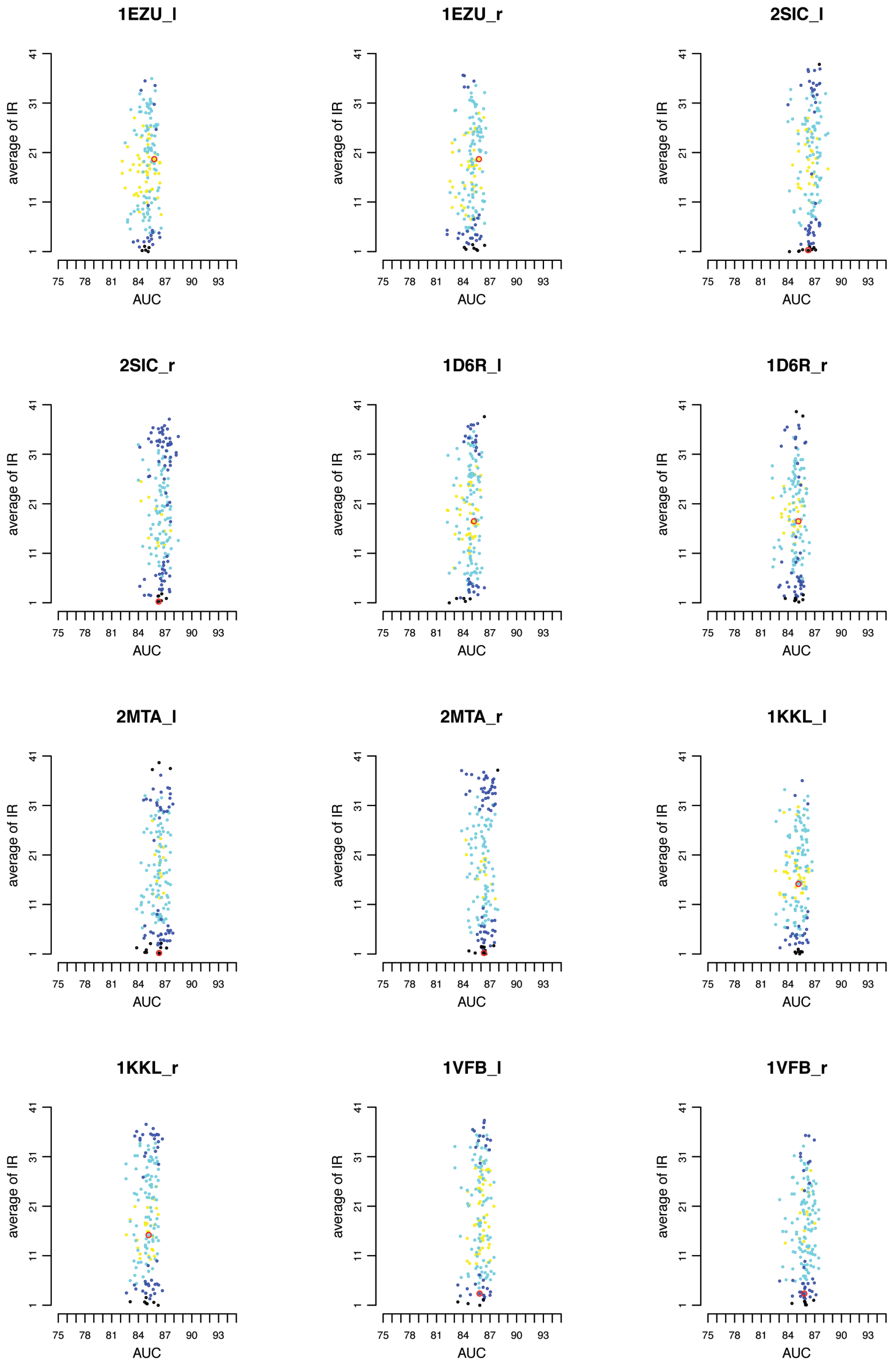

Figure S27: **IR analysis of false complexes against the true one, based on experimental interfaces computed over the full conformational space**. See legend in Fig. S24.

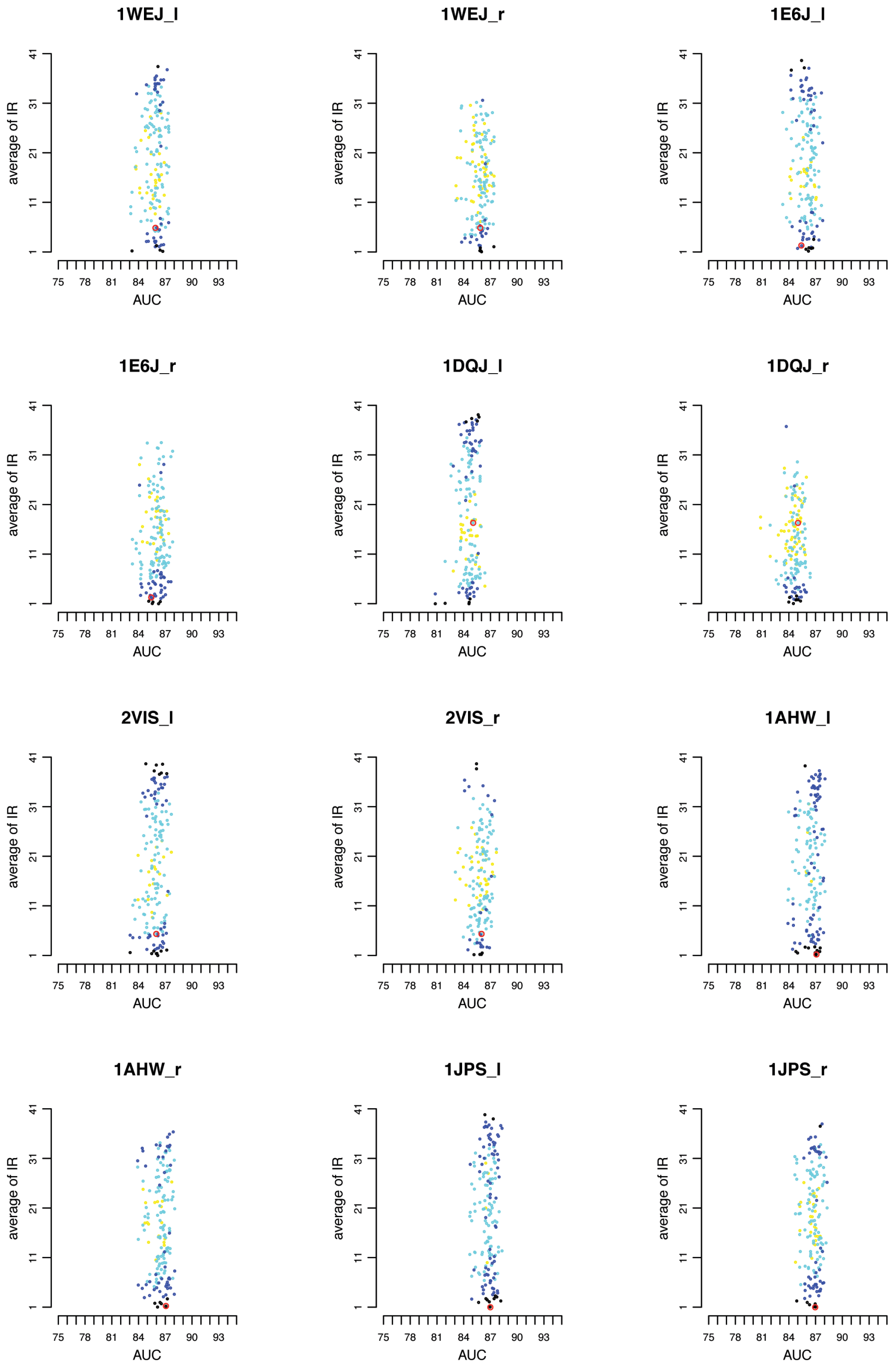

Figure S28: **IR analysis of false complexes against the true one, based on experimental interfaces computed over the full conformational space**. See legend in Fig. S24.

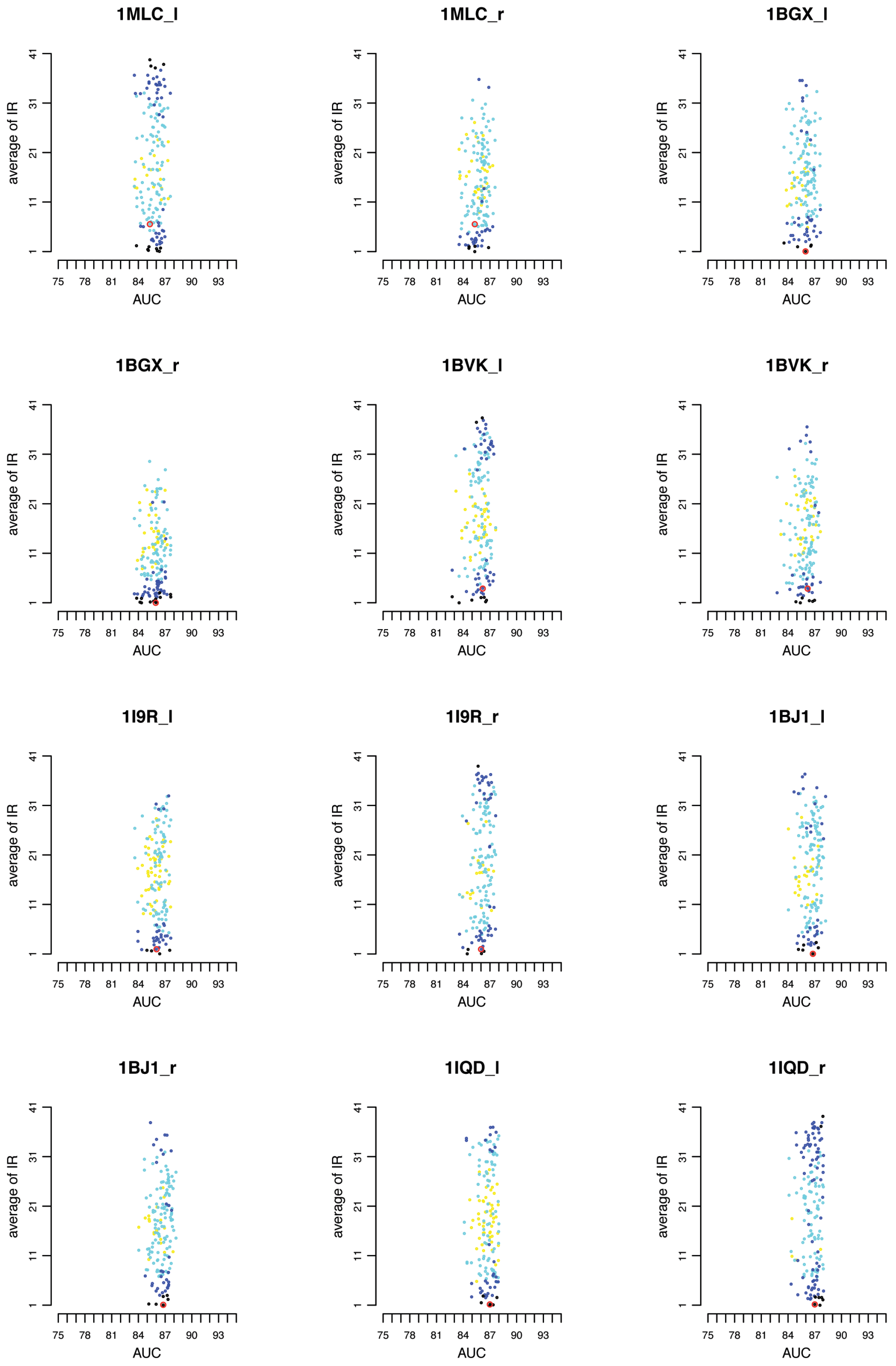

Figure S29: **IR analysis of false complexes against the true one, based on experimental interfaces computed over the full conformational space**. See legend in Fig. S24.

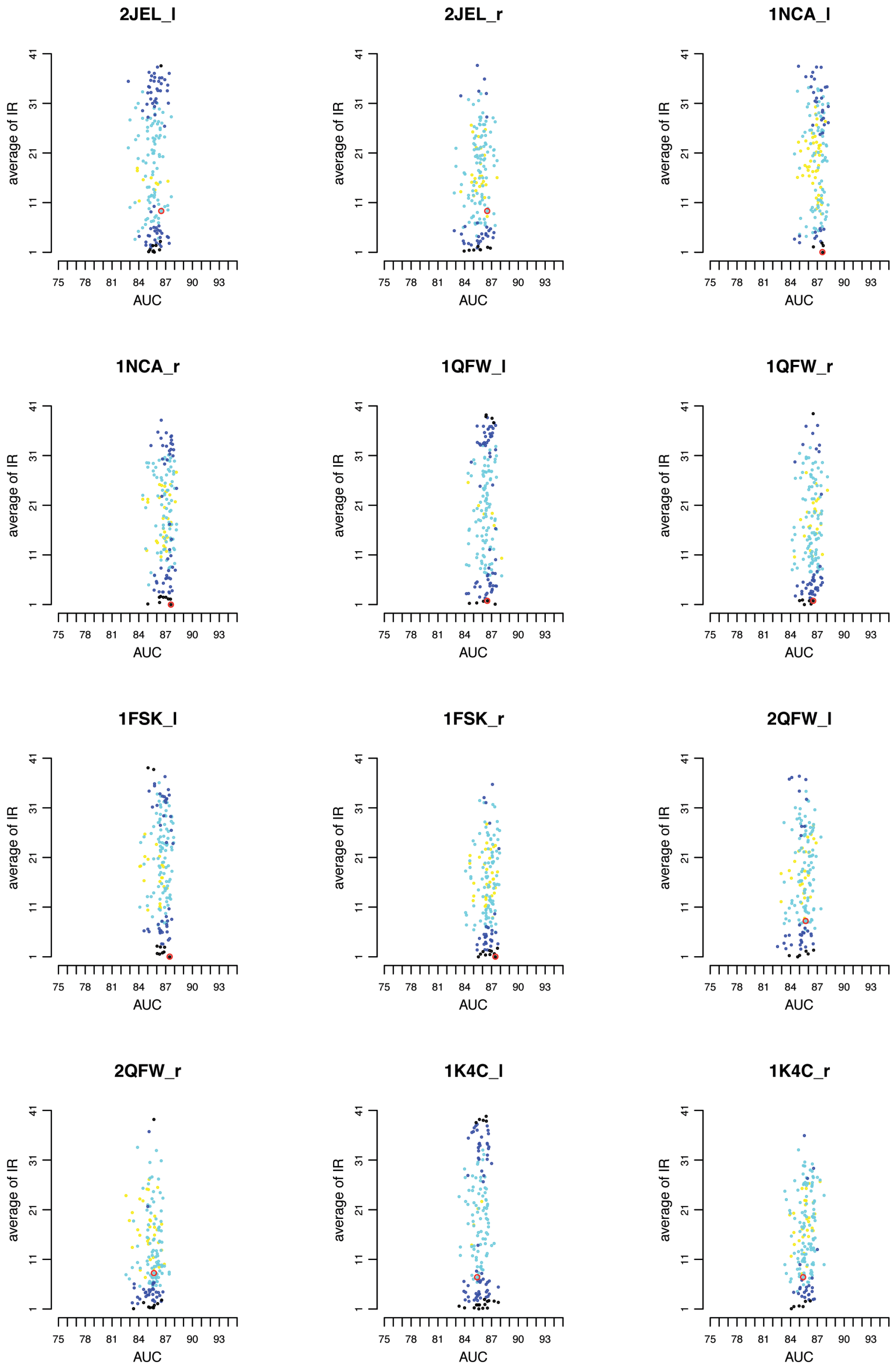

Figure S30: **IR analysis of false complexes against the true one, based on experimental interfaces computed over the full conformational space**. See legend in Fig. S24.

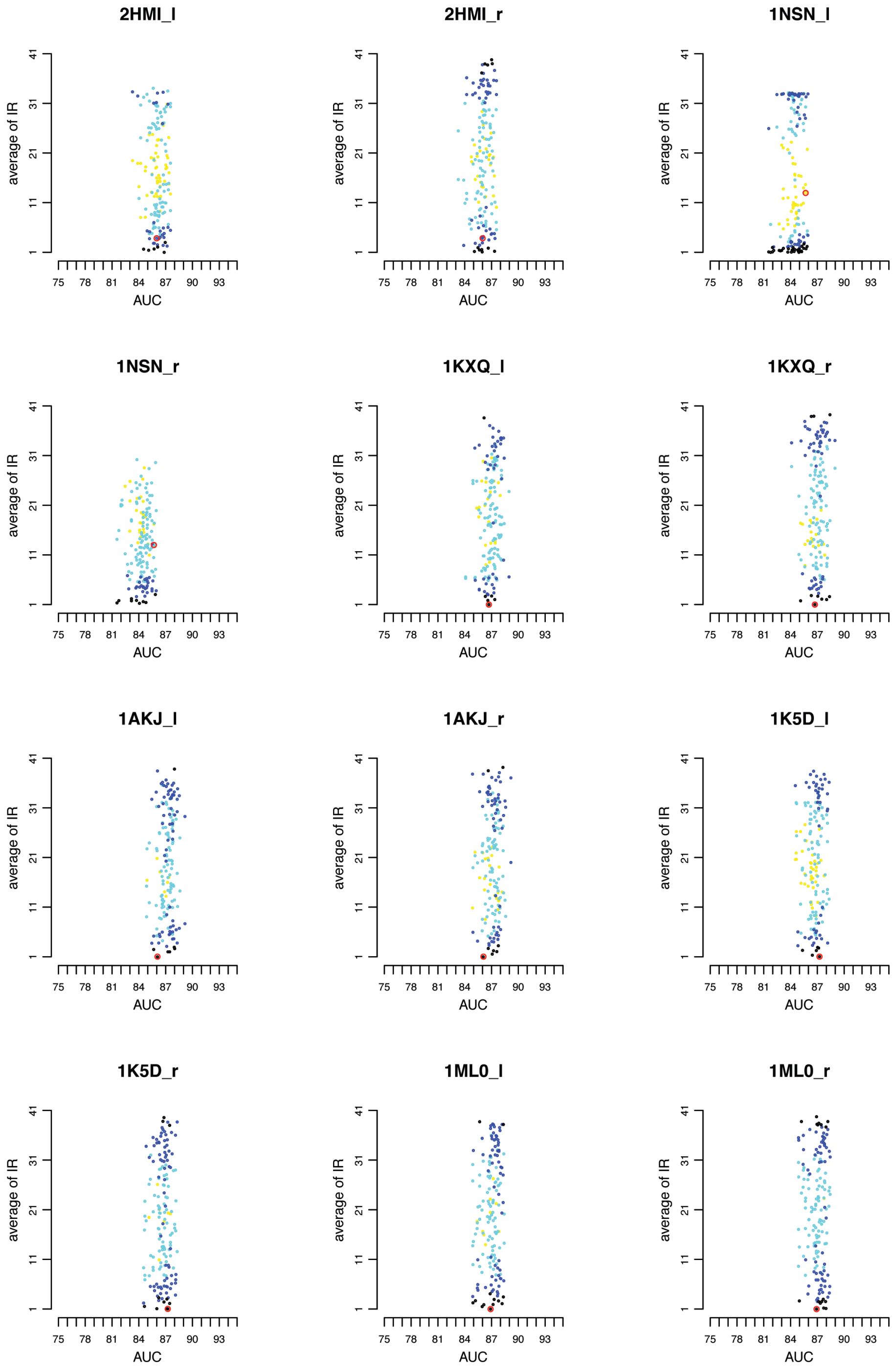

Figure S31: **IR analysis of false complexes against the true one, based on experimental interfaces computed over the full conformational space**. See legend in Fig. S24.

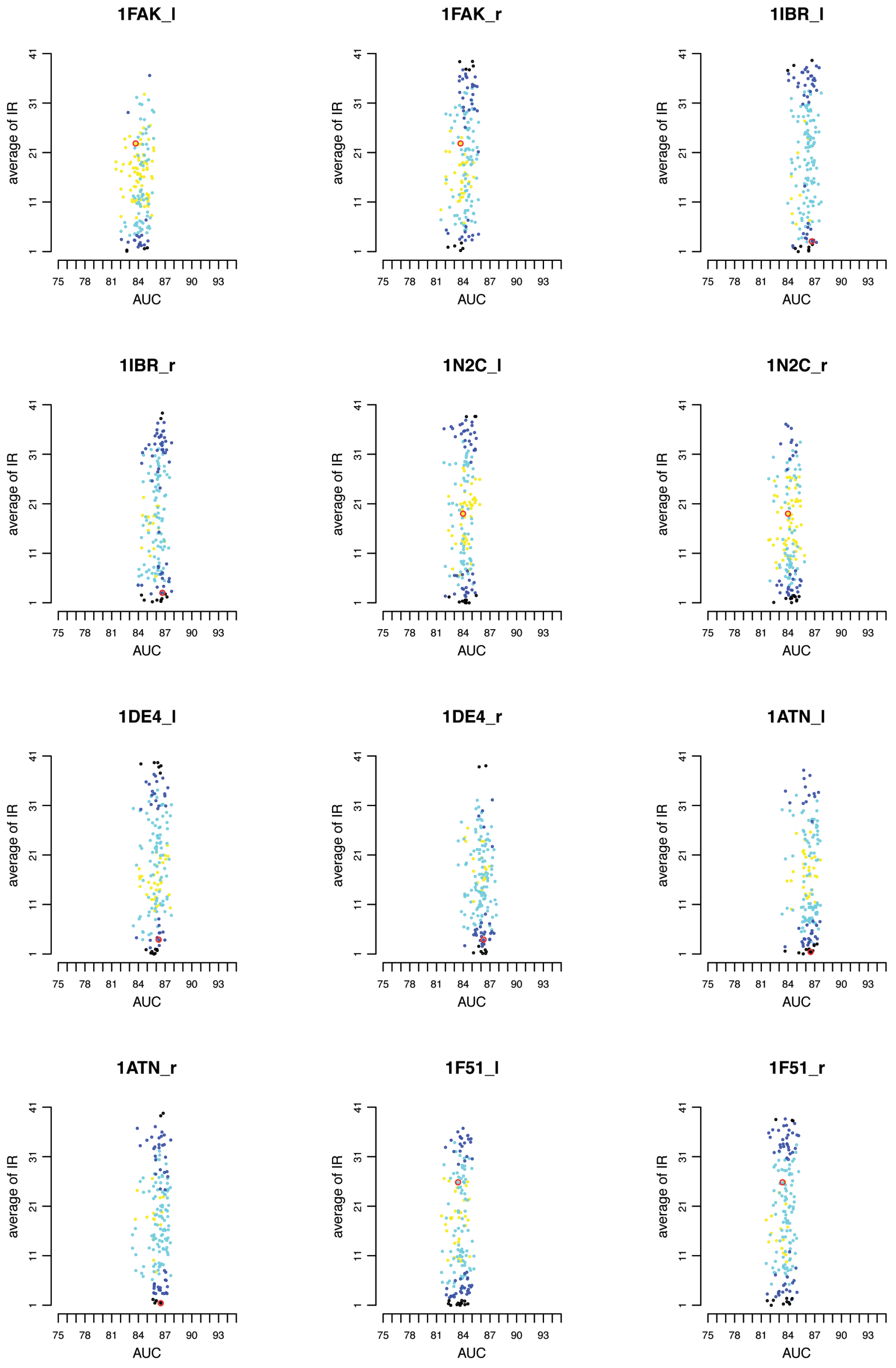

Figure S32: **IR analysis of false complexes against the true one, based on experimental interfaces computed over the full conformational space**. See legend in Fig. S24.

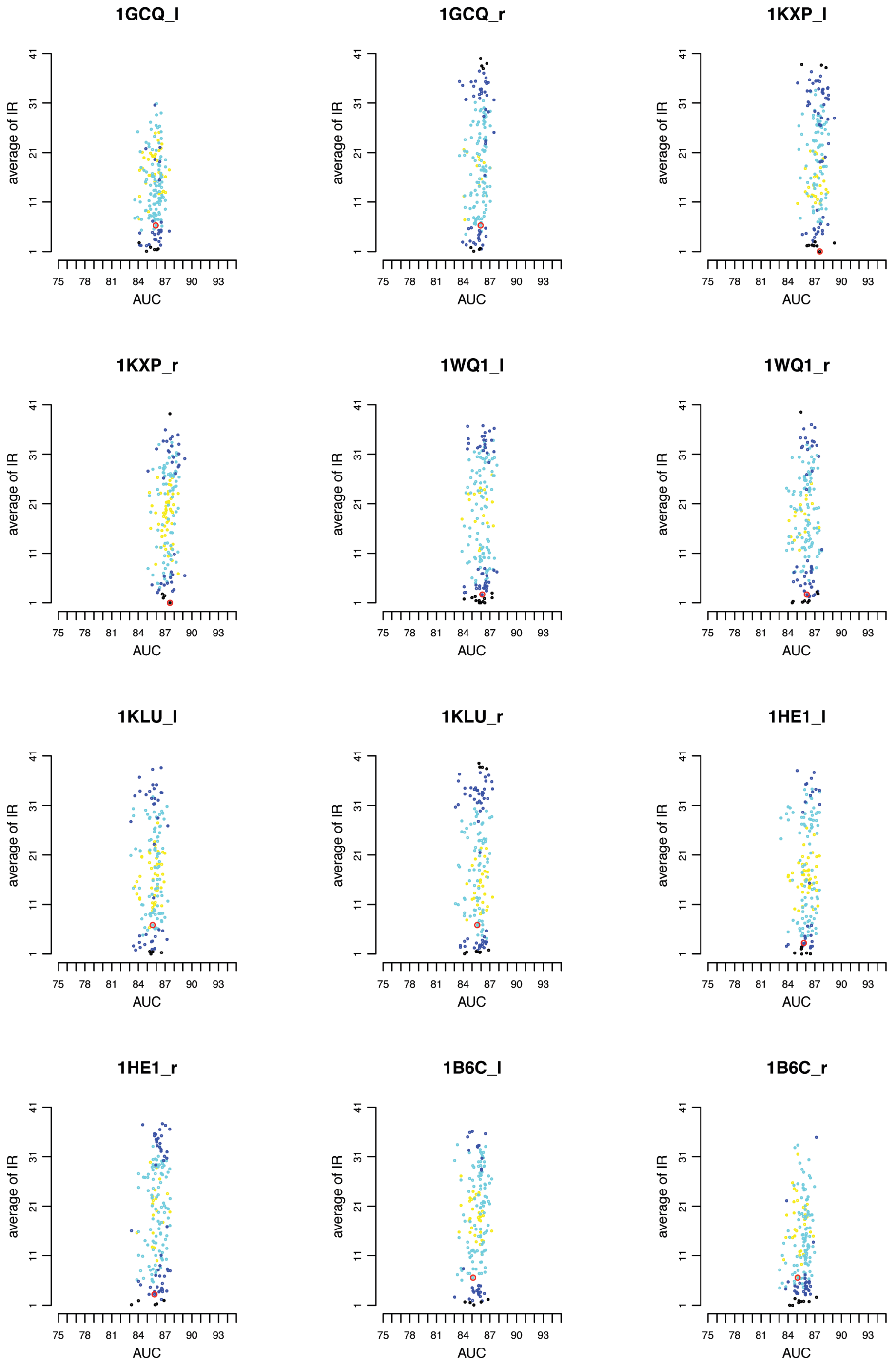

Figure S33: **IR analysis of false complexes against the true one, based on experimental interfaces computed over the full conformational space**. See legend in Fig. S24.

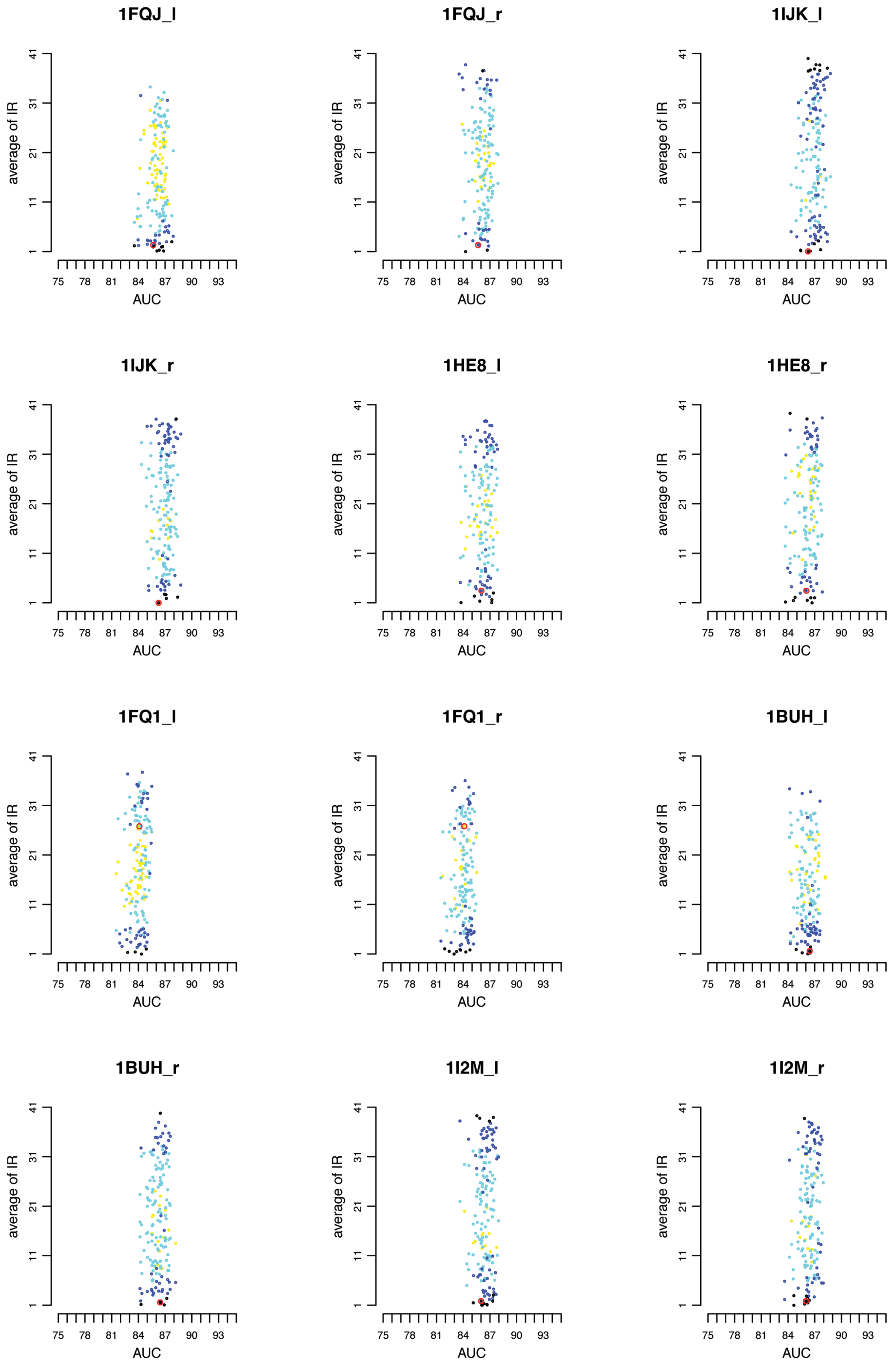

Figure S34: **IR analysis of false complexes against the true one, based on experimental interfaces computed over the full conformational space**. See legend in Fig. S24.

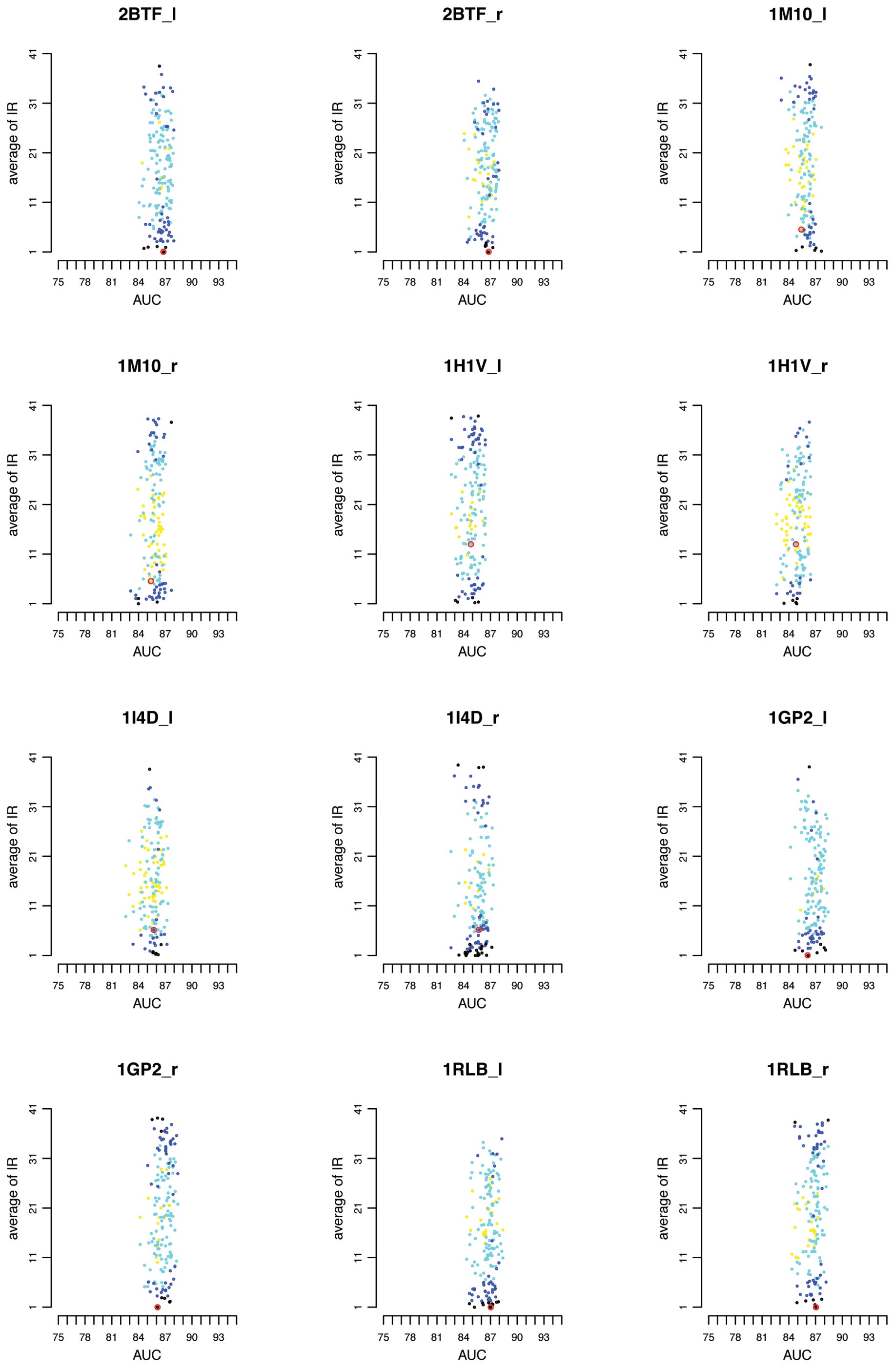

Figure S35: **IR analysis of false complexes against the true one, based on experimental interfaces computed over the full conformational space**. See legend in Fig. S24.

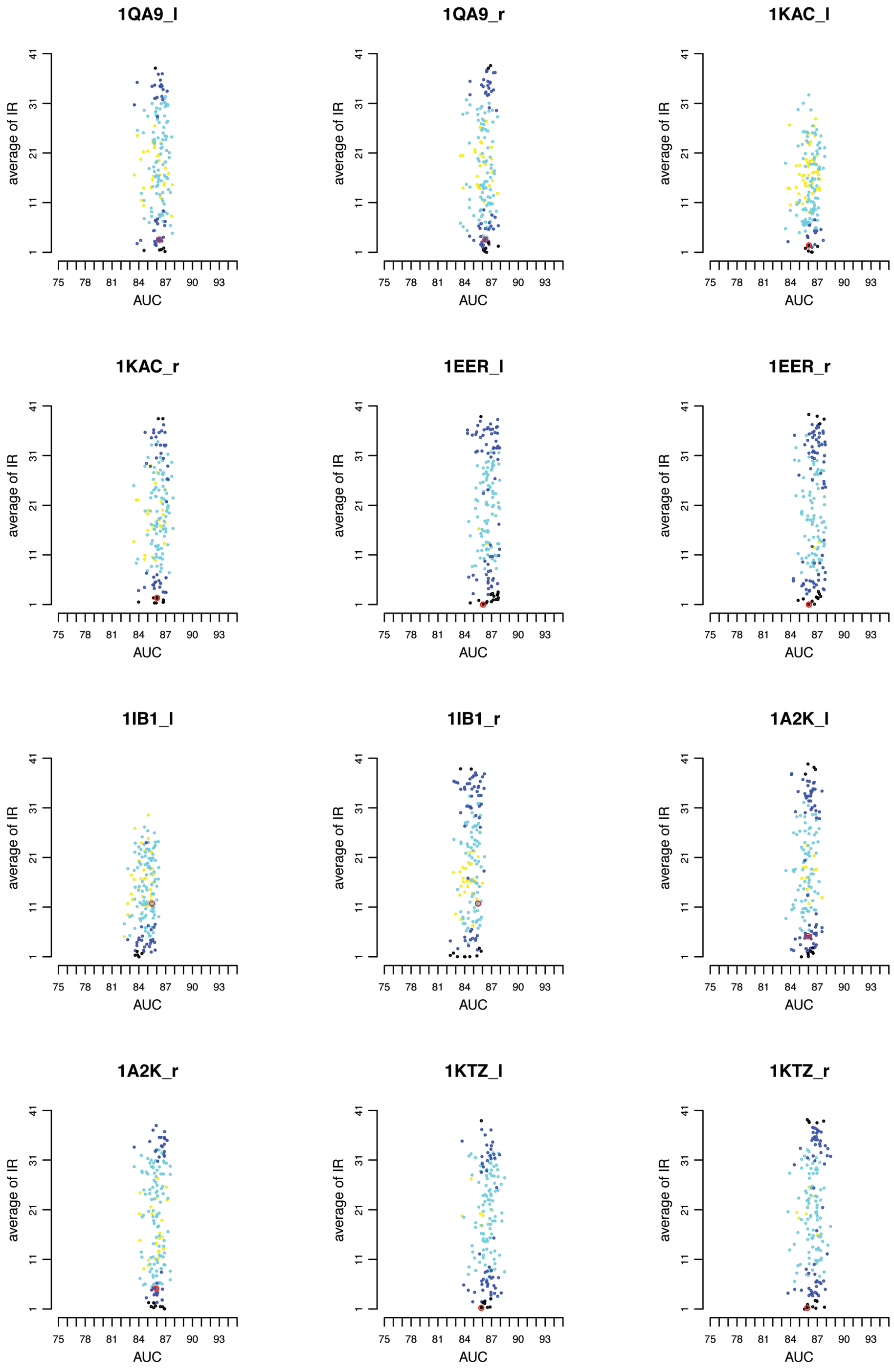

Figure S36: **IR analysis of false complexes against the true one, based on experimental interfaces computed over the full conformational space**. See legend in Fig. S24.

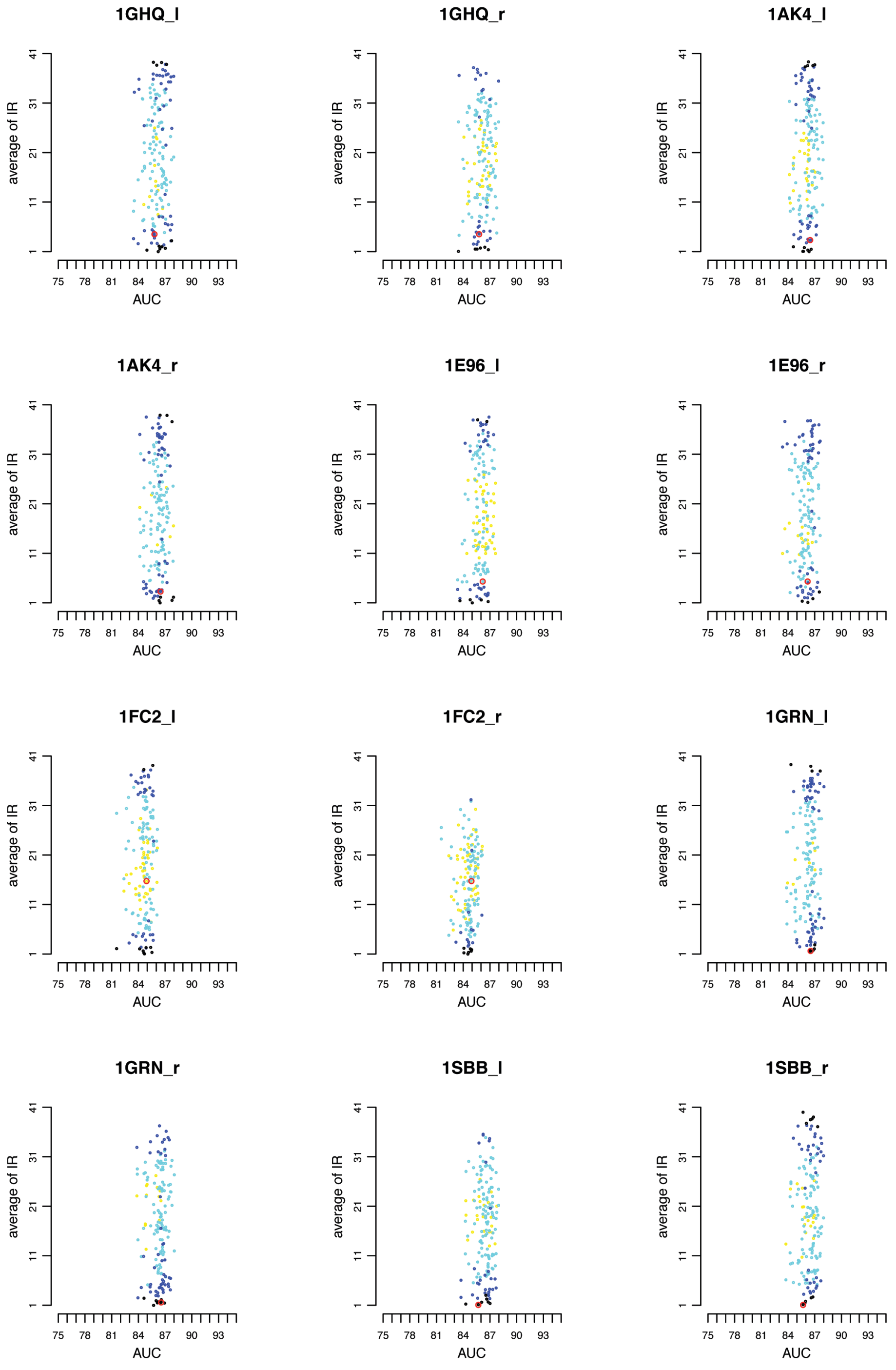

Figure S37: **IR analysis of false complexes against the true one, based on experimental interfaces computed over the full conformational space**. See legend in Fig. S24.

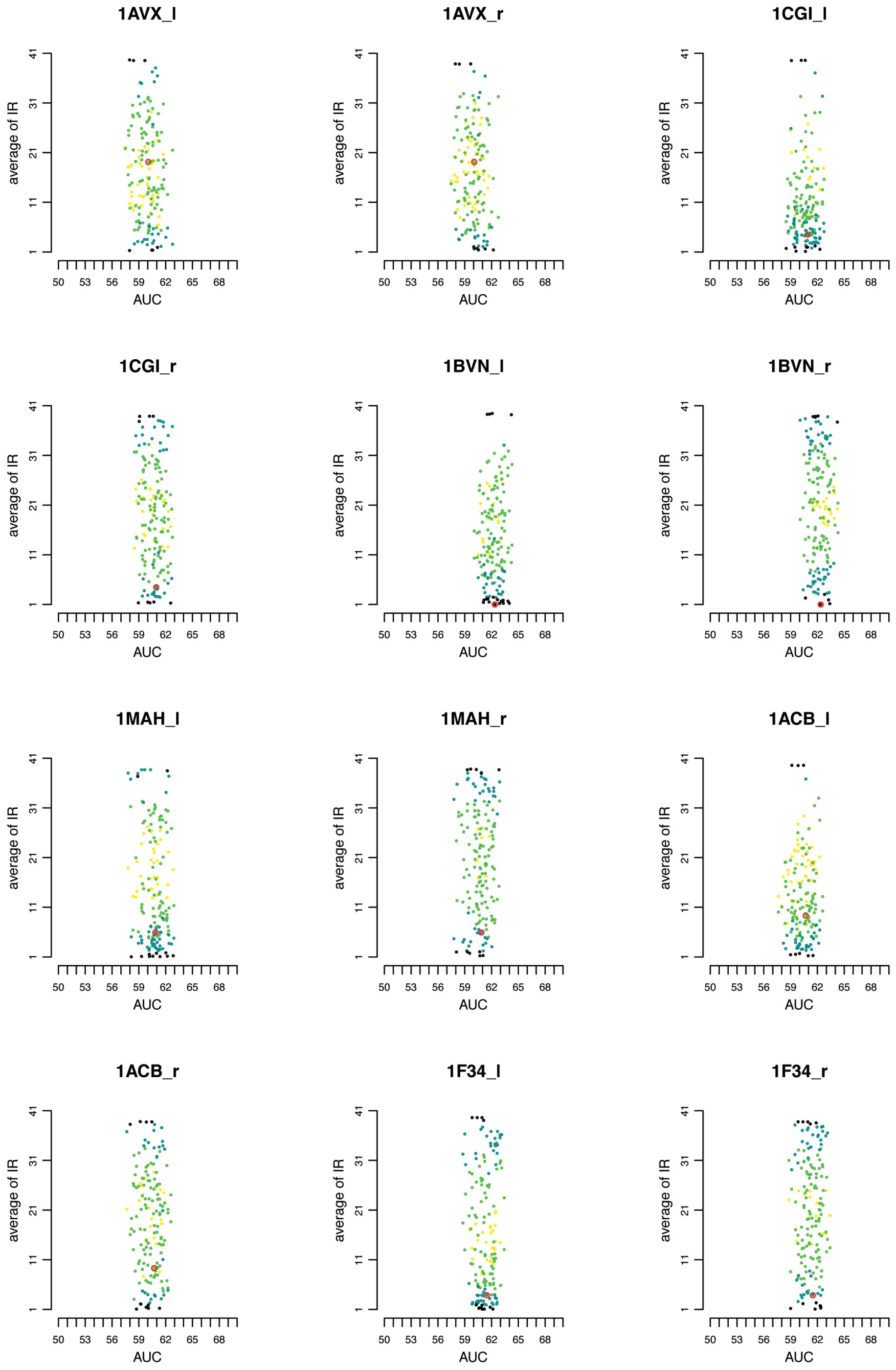

Figure S38: **IR analysis of false complexes against the true one, based on predicted interfaces and the full exploration of the conformational space**. Each monomer of the Mintseris Benchmark 2.0 is coupled with one of the 162 proteins (including the monomer itself) of the dataset forming either a false (161 cases) or the true complex (here we have not considered three complexes - 1DFJ, 1GCQ, 1ML0 - of the Mintseris Benchmark because JET predicted a very small interface that turned out to provide no FIR value). For each complex, we computed the corresponding average IR and average AUC over 100 random sets of 40 proteins. These values are reported as a point in a plot. Each plot contains 162 points. The red circle in each plot corresponds to the values of the true complex. Dots are colored in a scale from black, cyan, green, yellow. A color corresponds to the value of the standard deviation σ of the distribution of 100 IRs computed for a complex: black if $\sigma \leq 1$, cyan if $\sigma \leq 2$, green if $\sigma \leq 3$ and yellow otherwise (i.e. $\sigma > 3$).

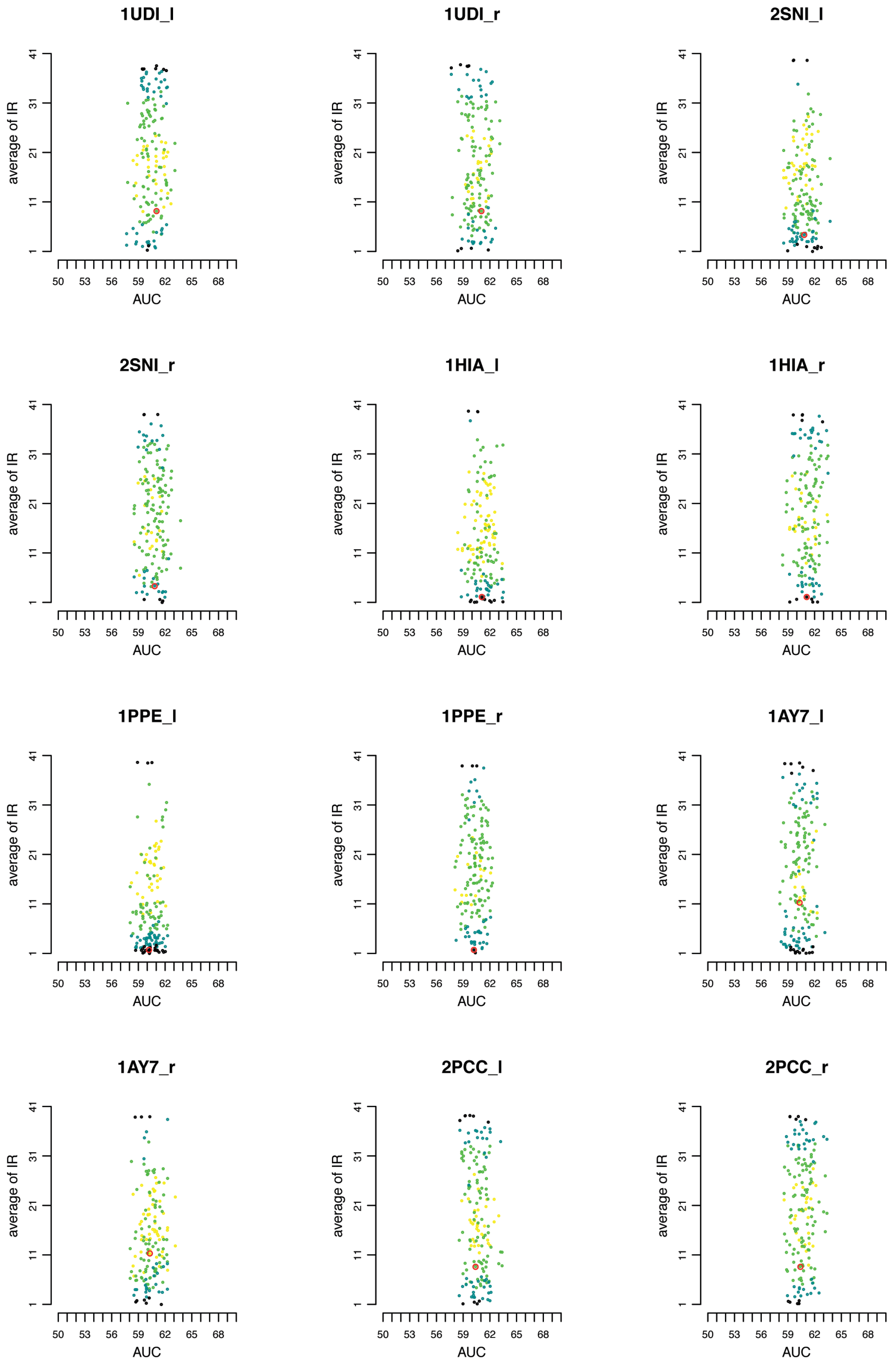

Figure S39: **IR analysis of false complexes against the true one, based on predicted interfaces computed over the full conformational space**. See legend in Fig. S38.

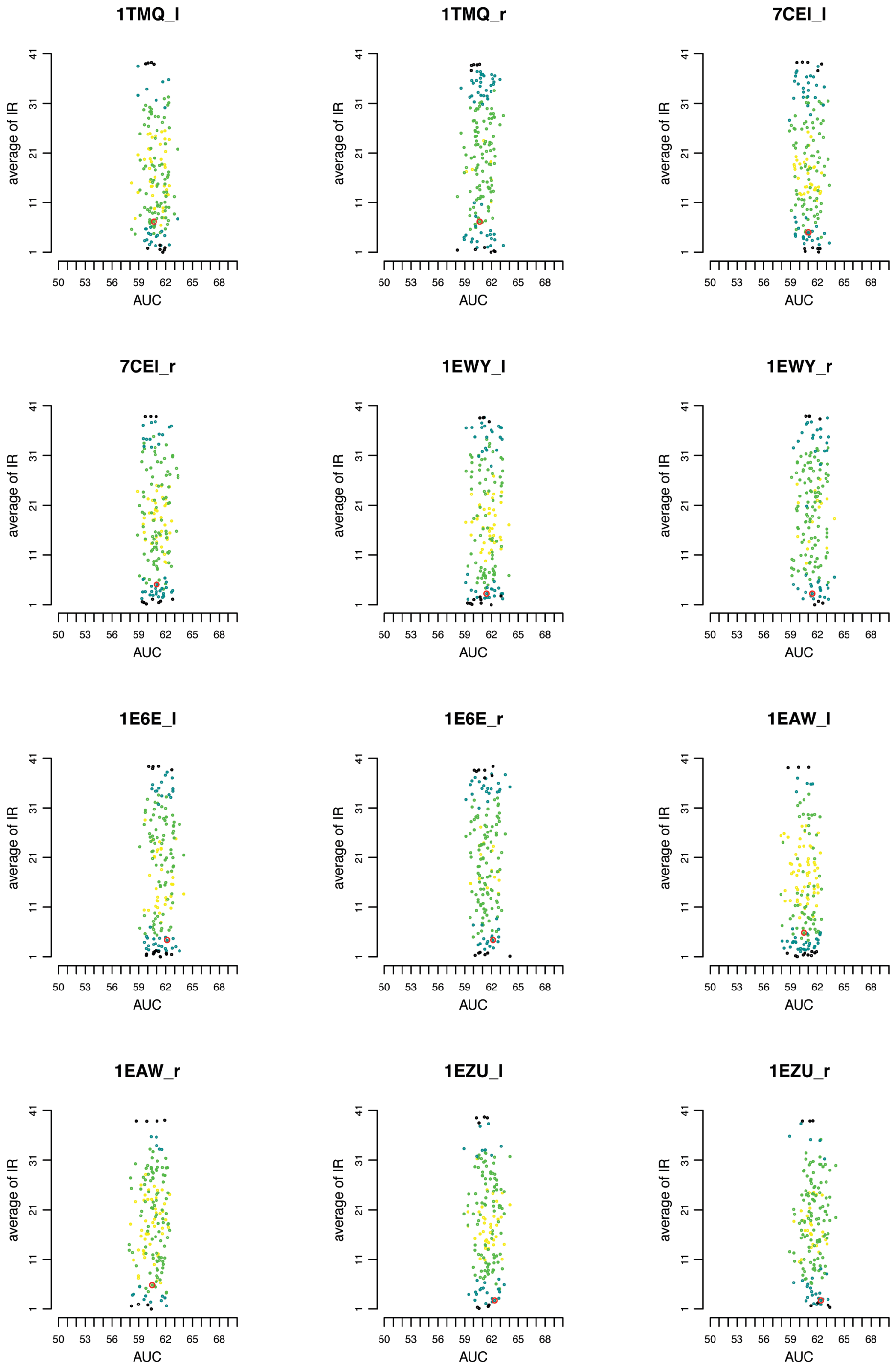

Figure S40: **IR analysis of false complexes against the true one, based on predicted interfaces computed over the full conformational space**. See legend in Fig. S38.

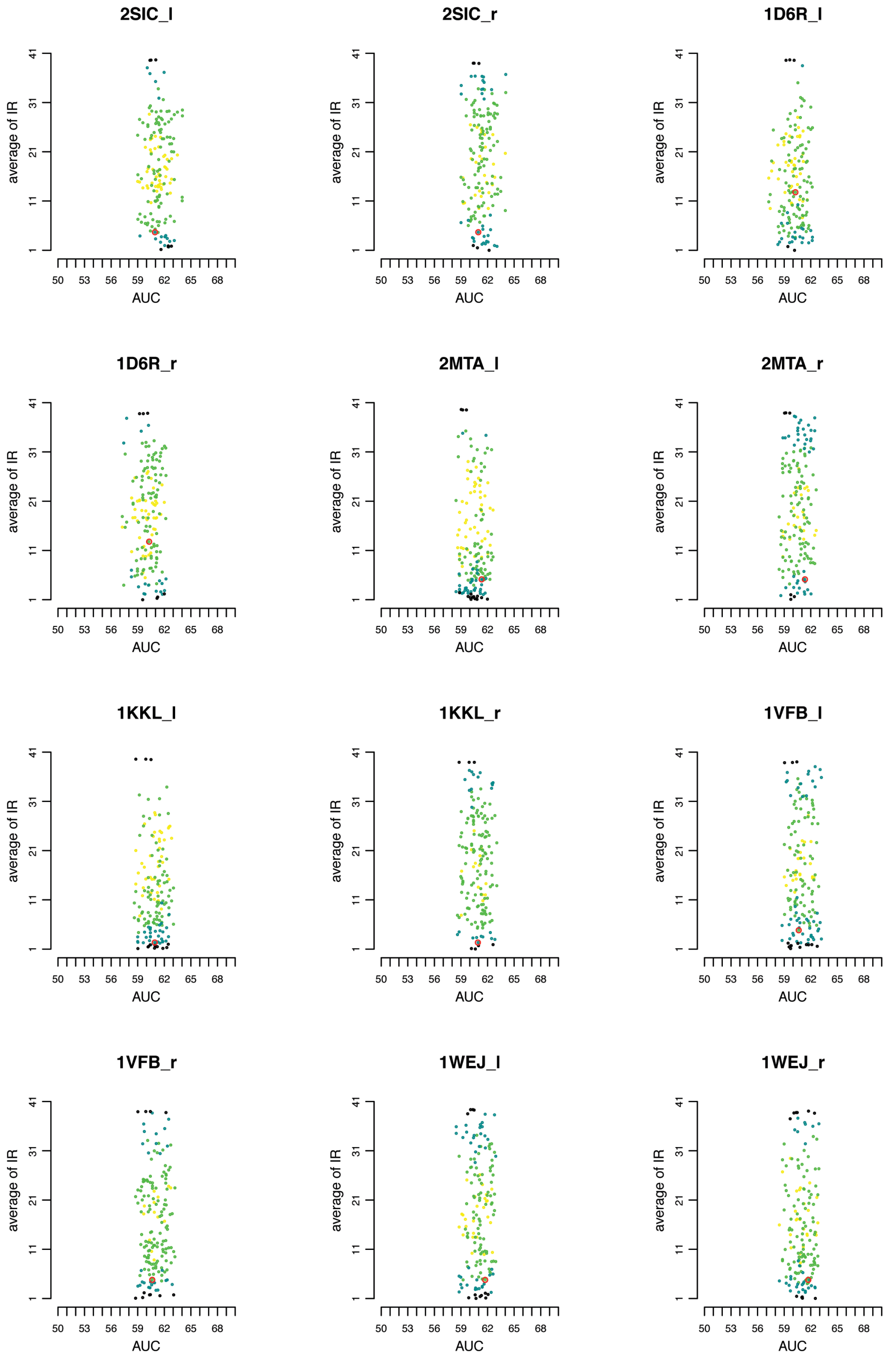

Figure S41: **IR analysis of false complexes against the true one, based on predicted interfaces computed over the full conformational space**. See legend in Fig. S38.

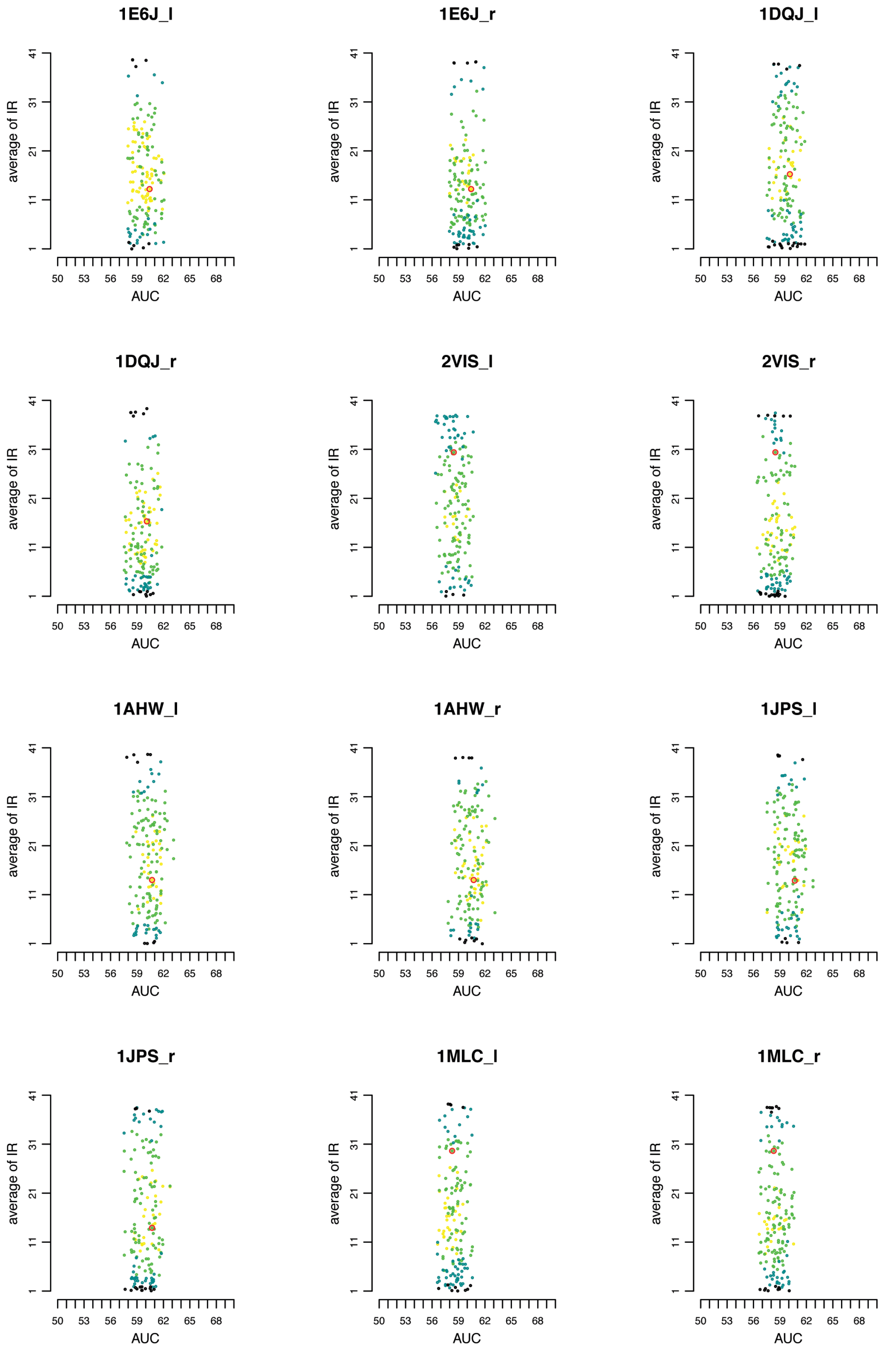

Figure S42: **IR analysis of false complexes against the true one, based on predicted interfaces computed over the full conformational space**. See legend in Fig. S38.

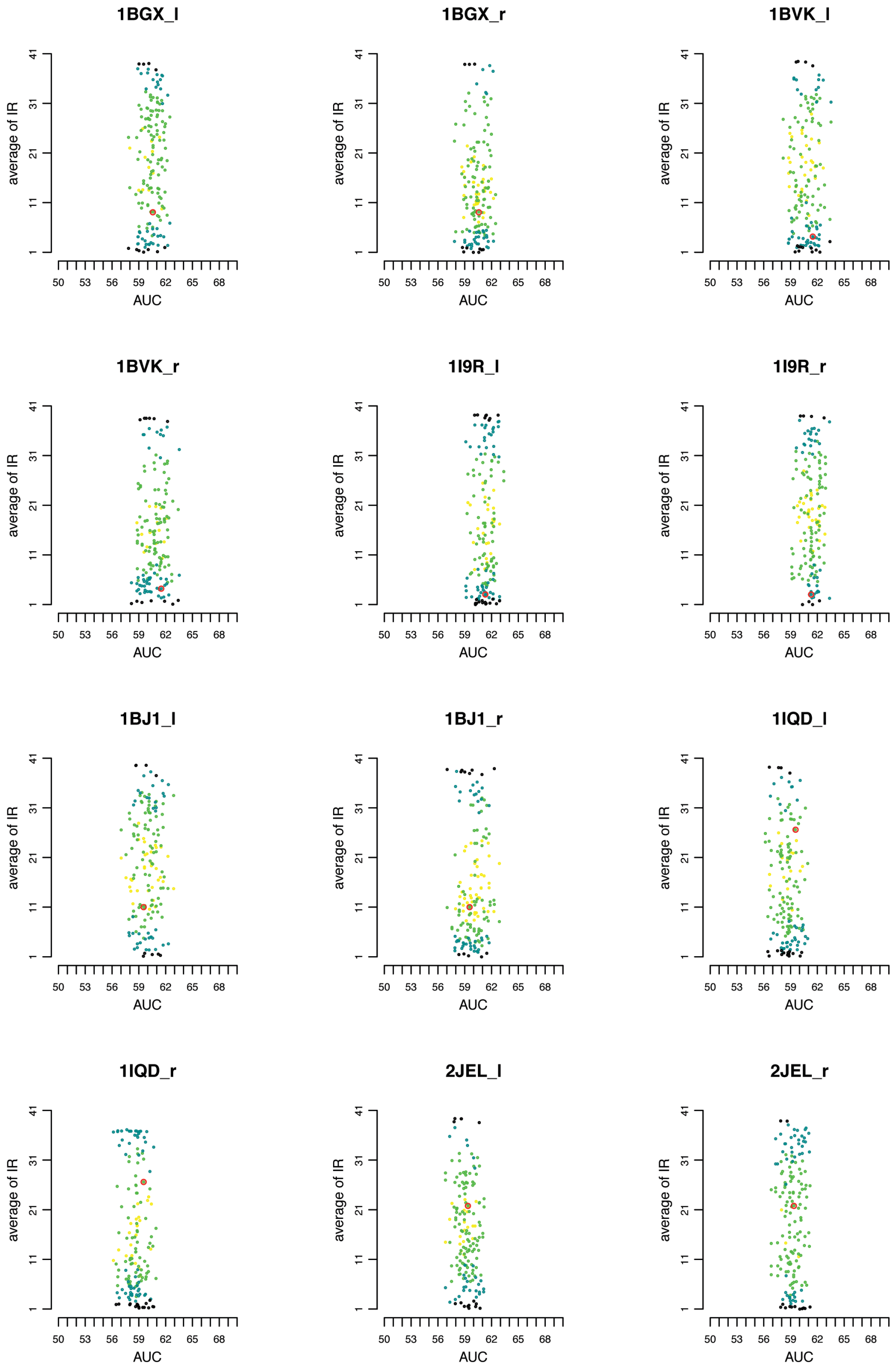

Figure S43: **IR analysis of false complexes against the true one, based on predicted interfaces computed over the full conformational space**. See legend in Fig. S38.

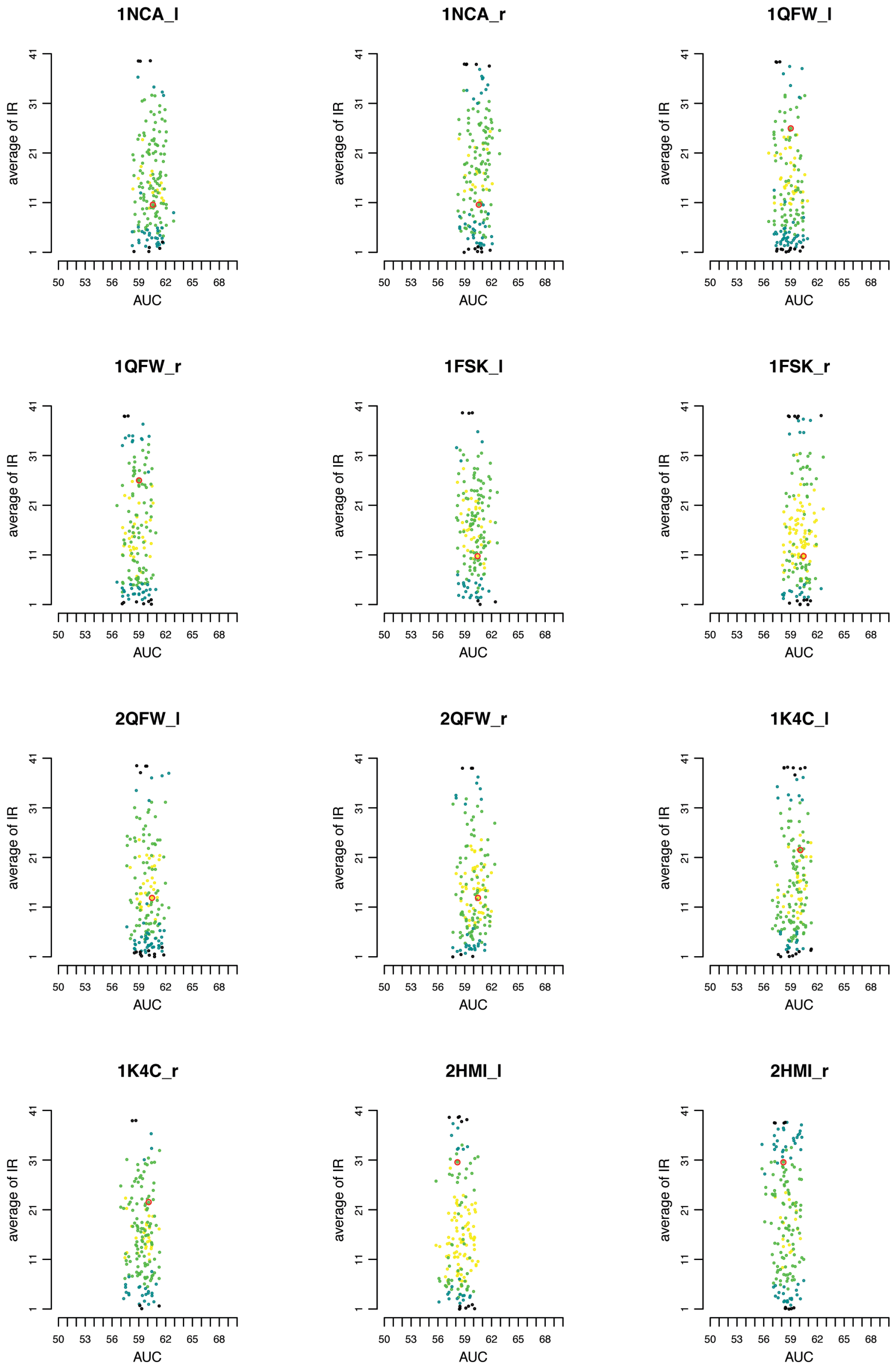

Figure S44: **IR analysis of false complexes against the true one, based on predicted interfaces computed over the full conformational space**. See legend in Fig. S38.

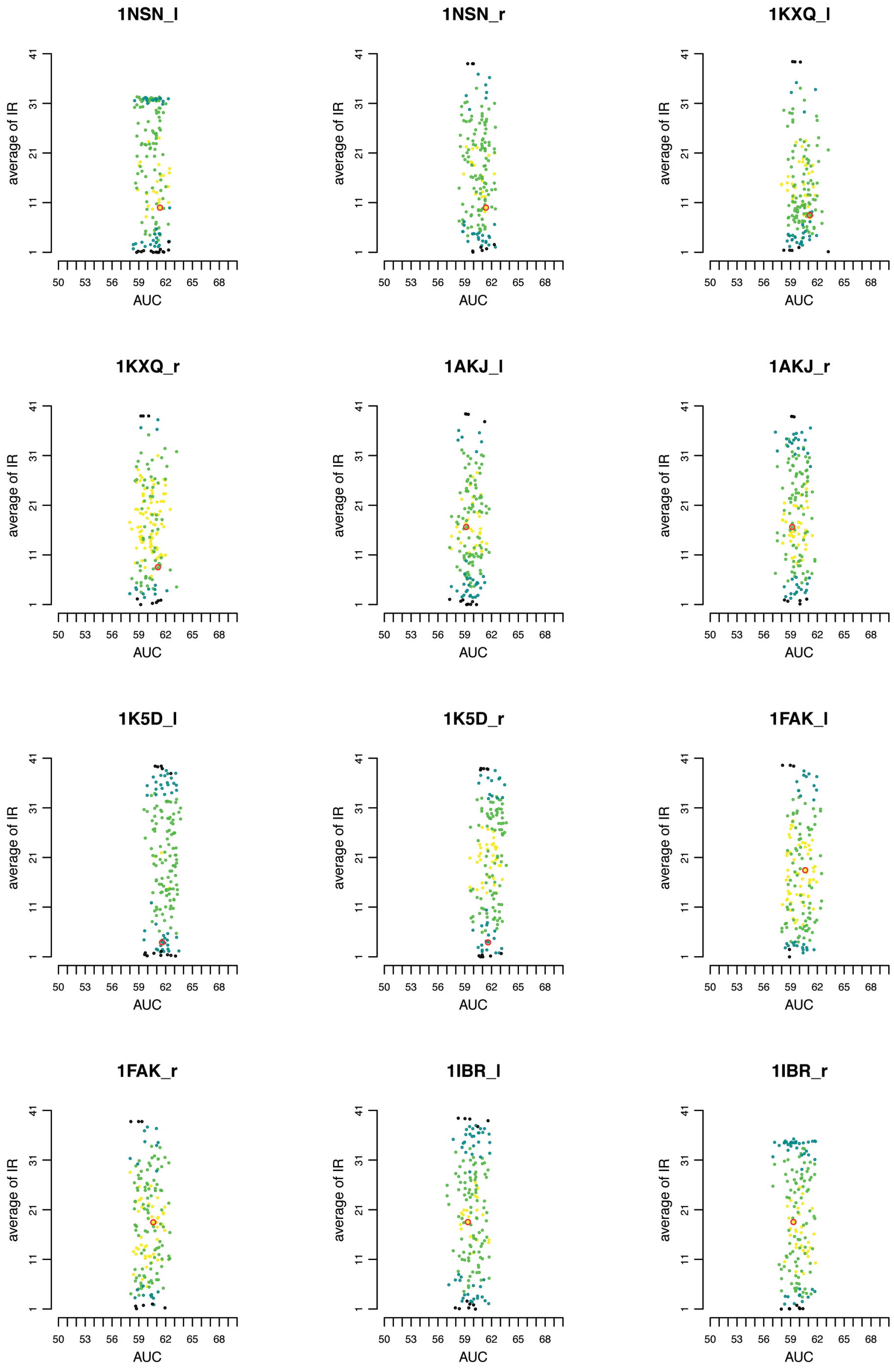

Figure S45: **IR analysis of false complexes against the true one, based on predicted interfaces computed over the full conformational space**. See legend in Fig. S38.

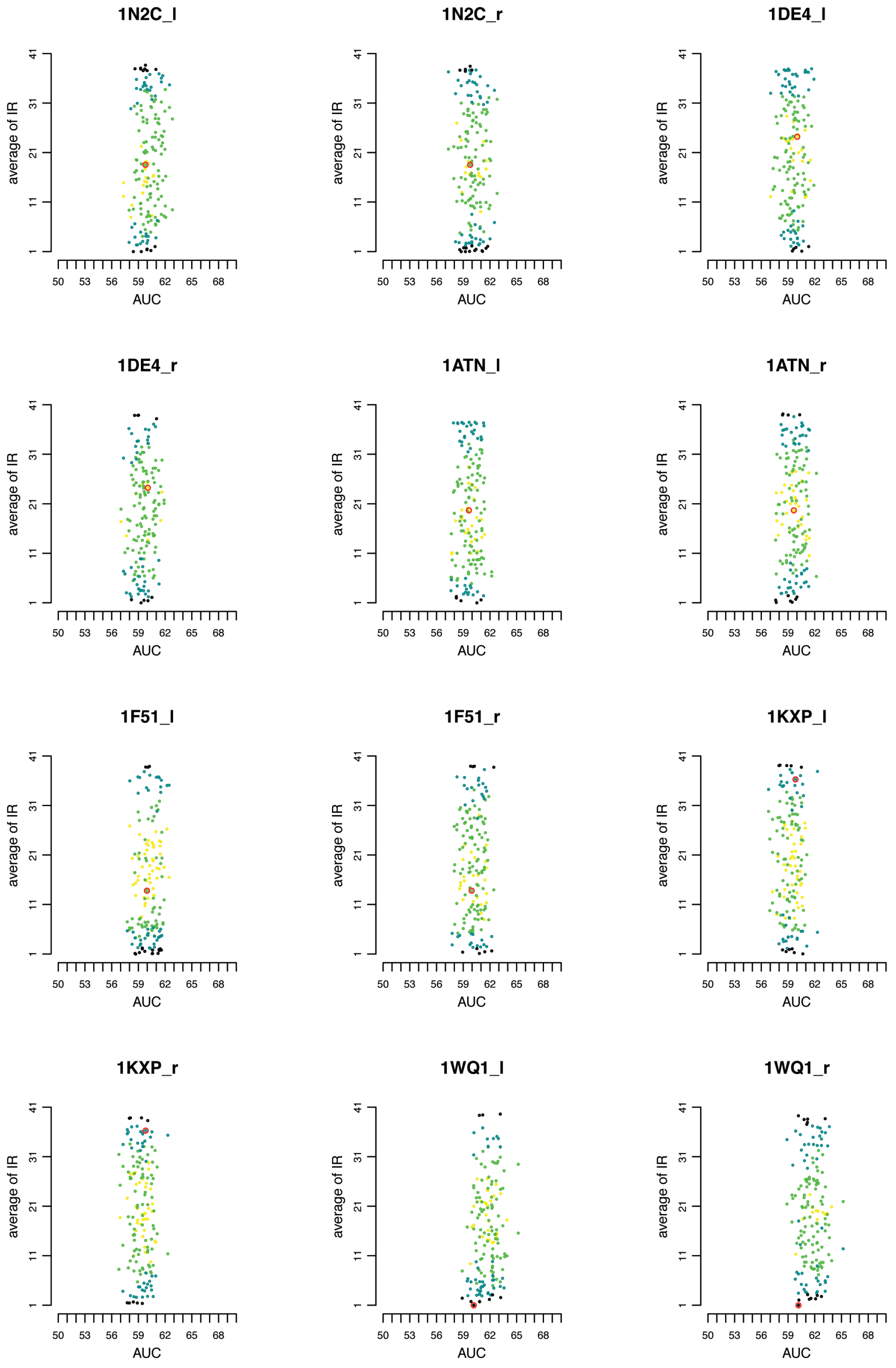

Figure S46: **IR analysis of false complexes against the true one, based on predicted interfaces computed over the full conformational space**. See legend in Fig. S38.

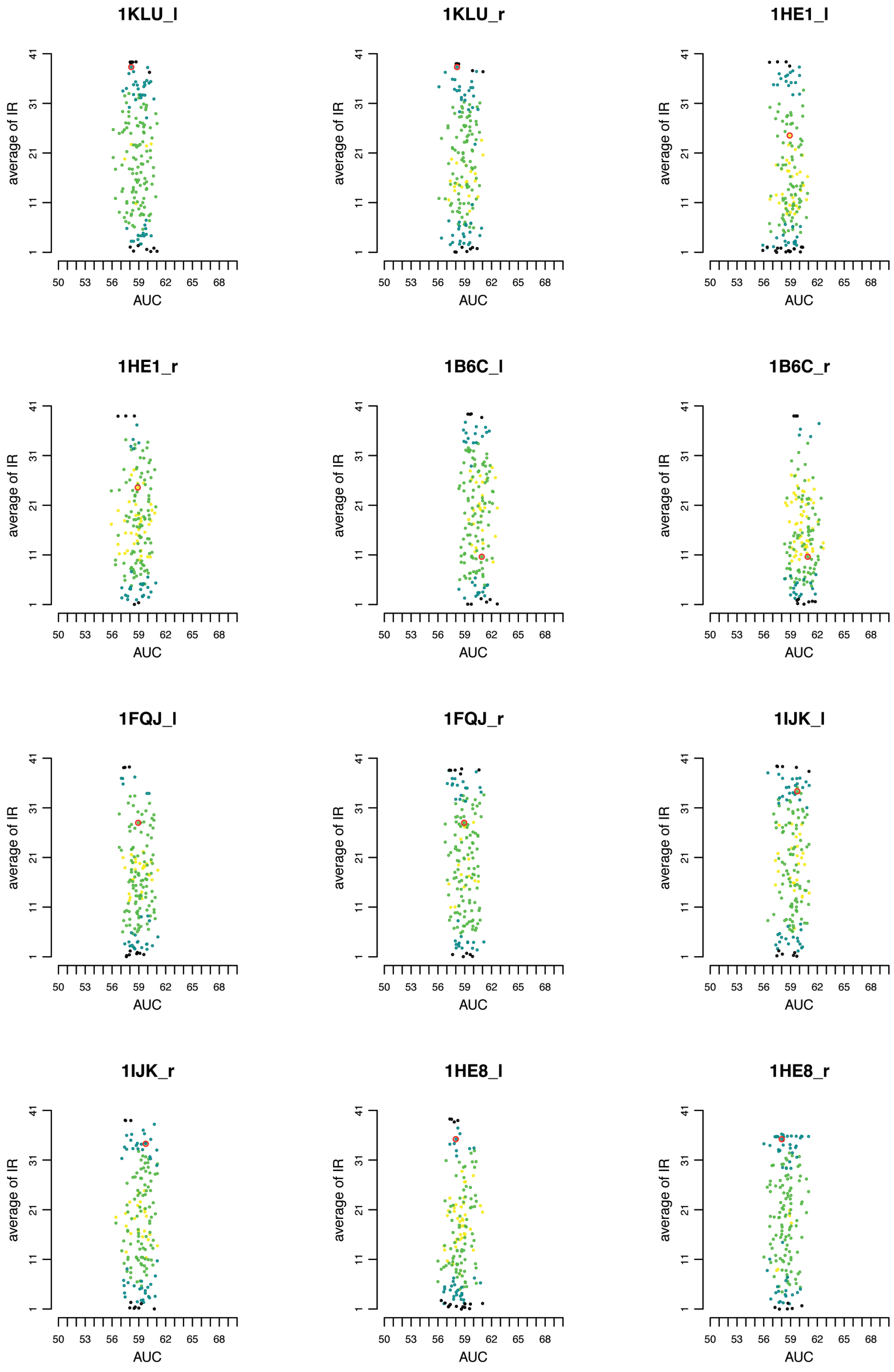

Figure S47: **IR analysis of false complexes against the true one, based on predicted interfaces computed over the full conformational space**. See legend in Fig. S38.

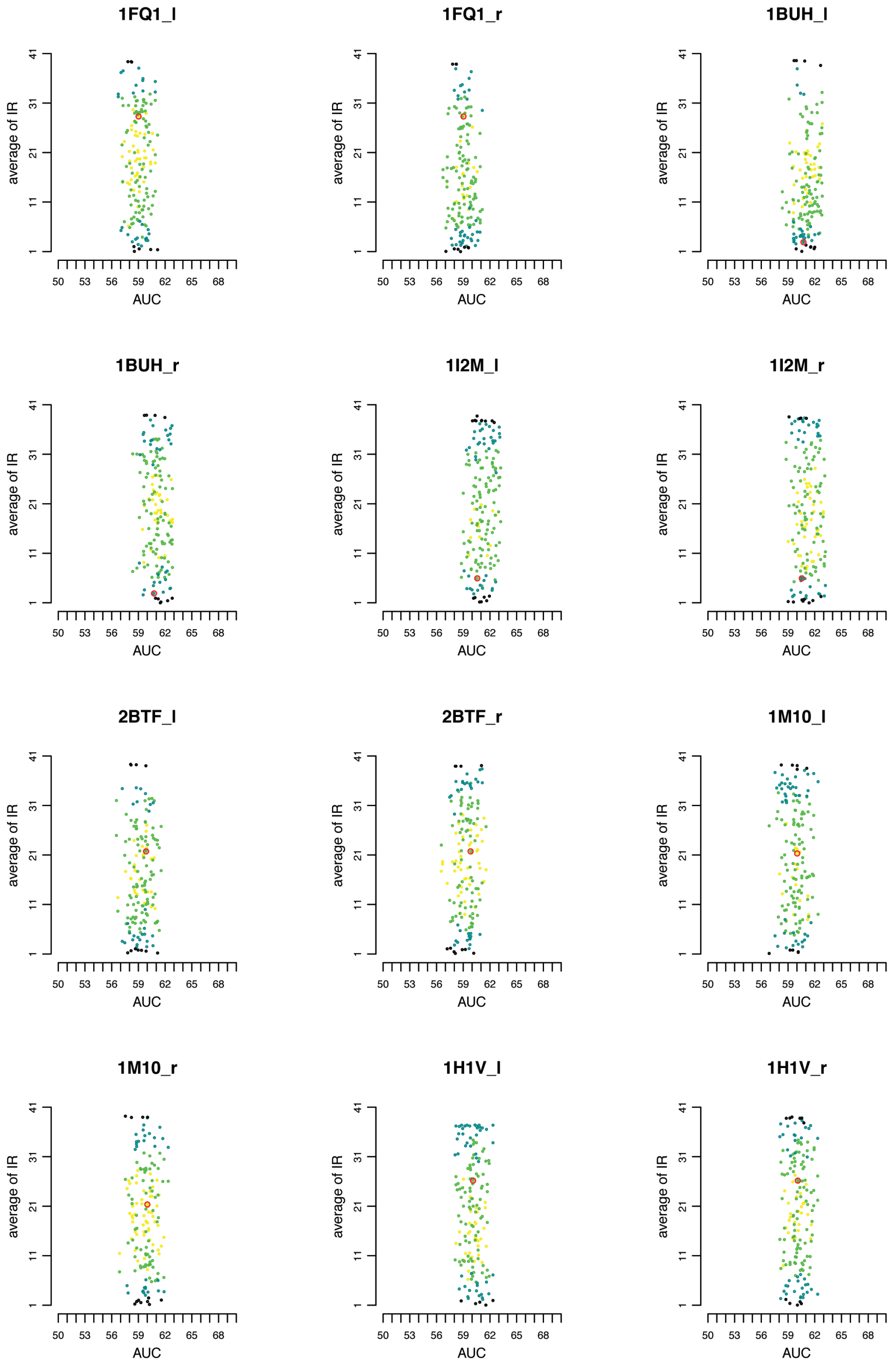

Figure S48: **IR analysis of false complexes against the true one, based on predicted interfaces computed over the full conformational space**. See legend in Fig. S38.

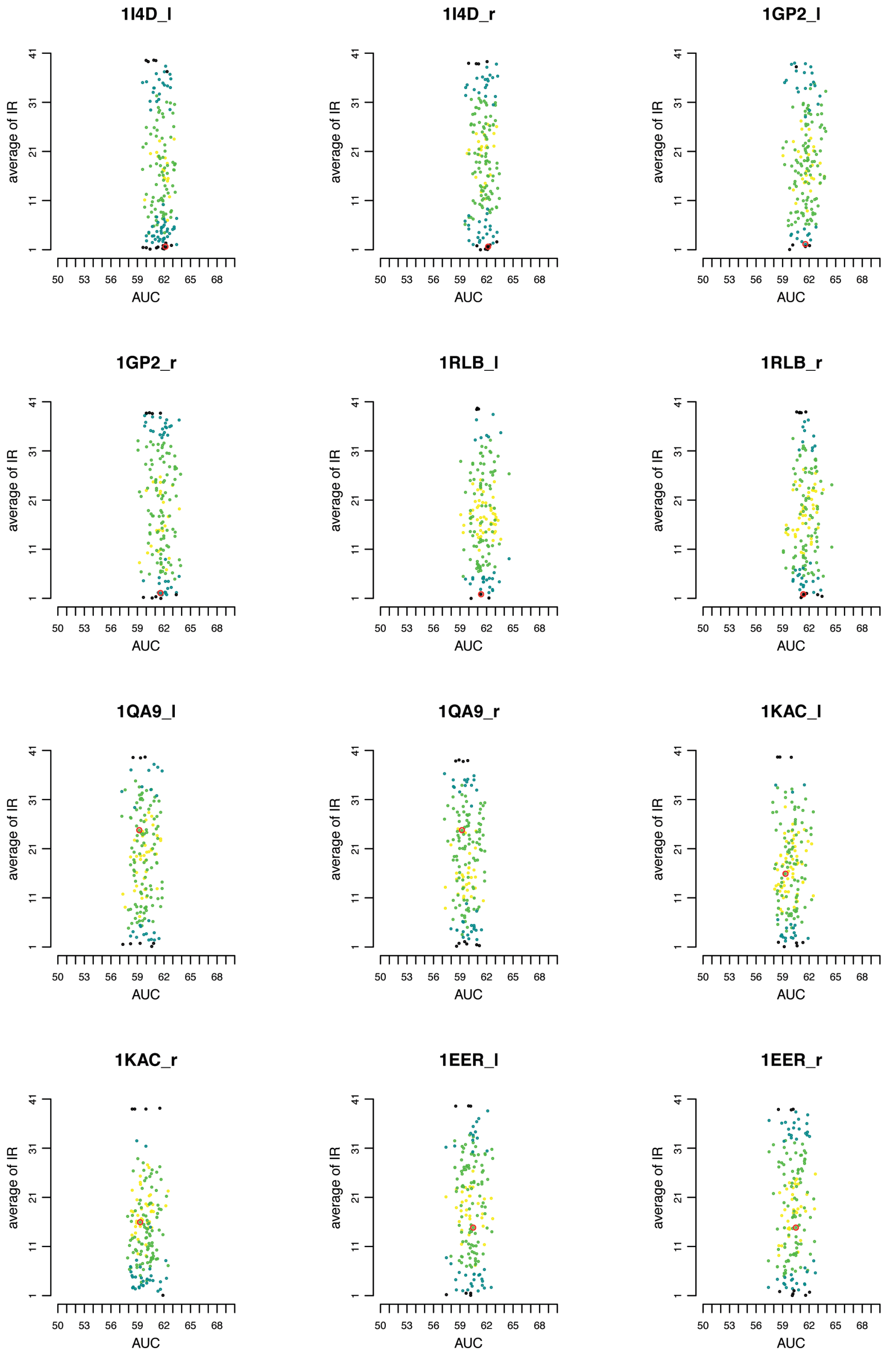

Figure S49: **IR analysis of false complexes against the true one, based on predicted interfaces computed over the full conformational space**. See legend in Fig. S38.

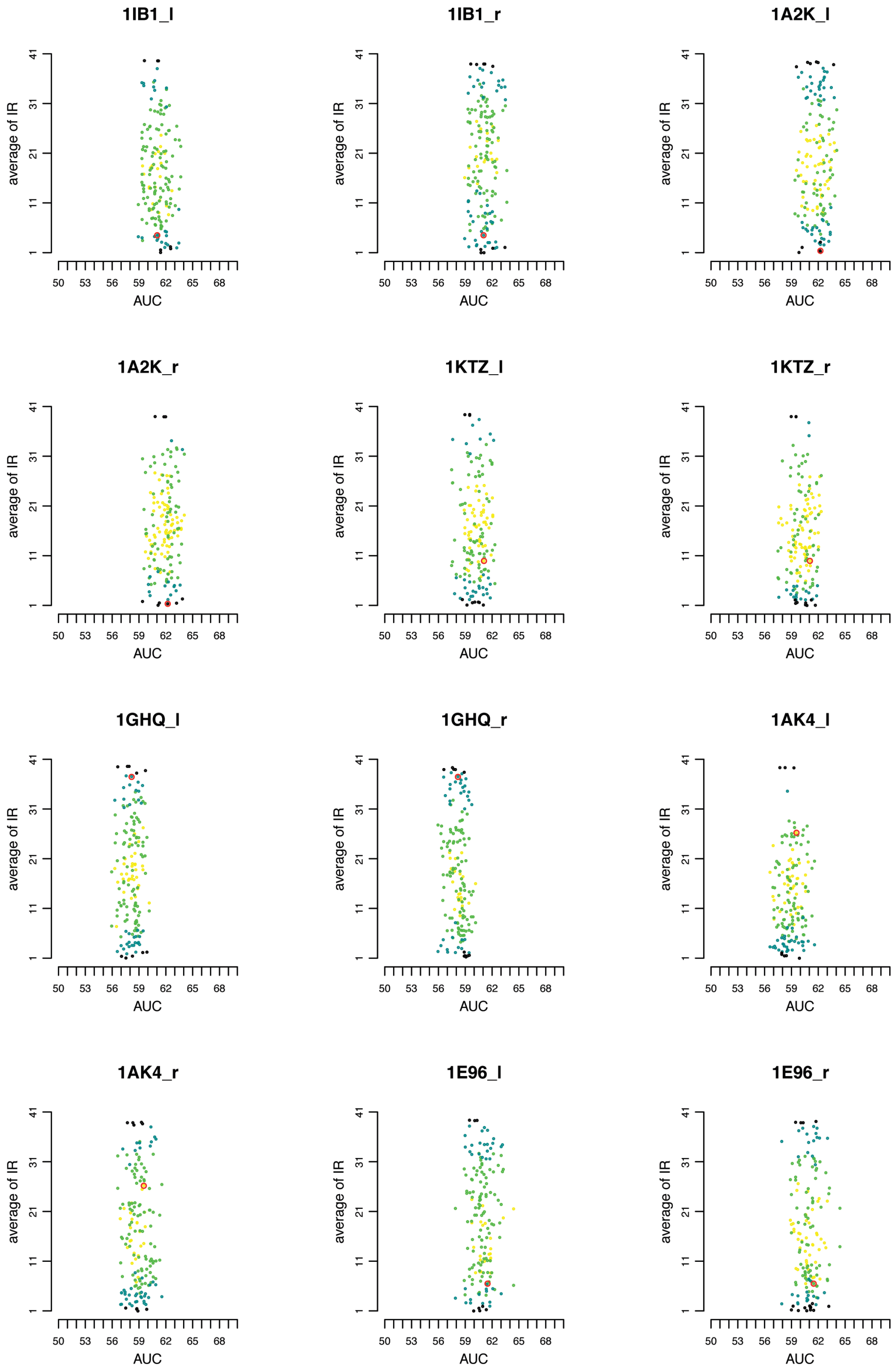

Figure S50: **IR analysis of false complexes against the true one, based on predicted interfaces computed over the full conformational space**. See legend in Fig. S38.

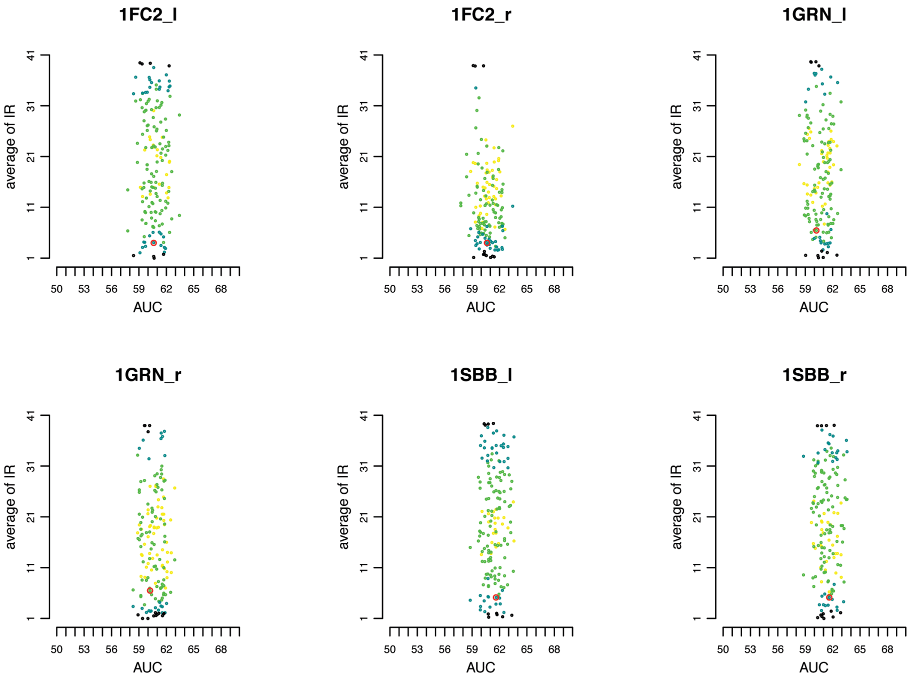

Figure S51: **IR analysis of false complexes against the true one, based on predicted interfaces computed over the full conformational space**. See legend in Fig. S38.

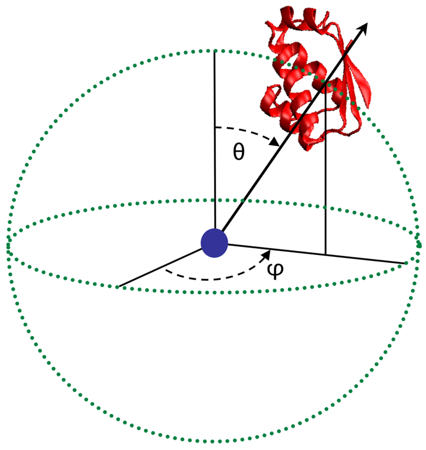

Figure S52: **Schematic view of the docking algorithm.** For each starting position, defined by the Euler angles θ and ϕ, uniformly spaced around the receptor protein (blue point), the orientation and distance of ligand protein (in red) from the receptor is optimized during energy minimization.

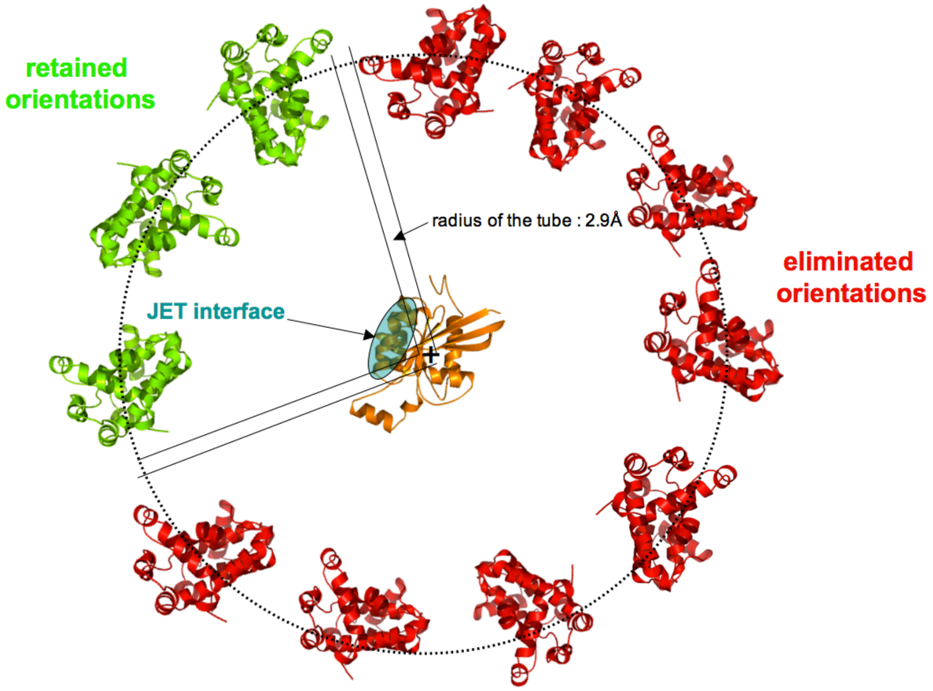

Figure S53: **Cone defined around the predicted interface**. For each receptor, one predicts the location of its interface based on evolutionary information and computes a region around it that approximate the location where the real interface is supposed to lie. Docking is restricted to this region of the receptor.

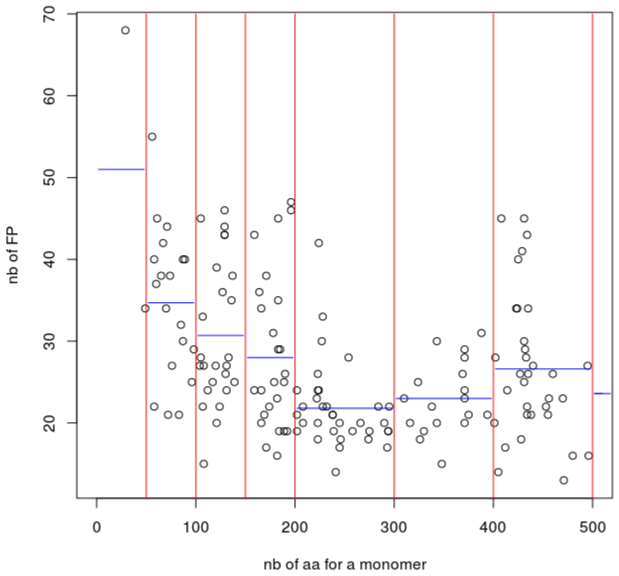

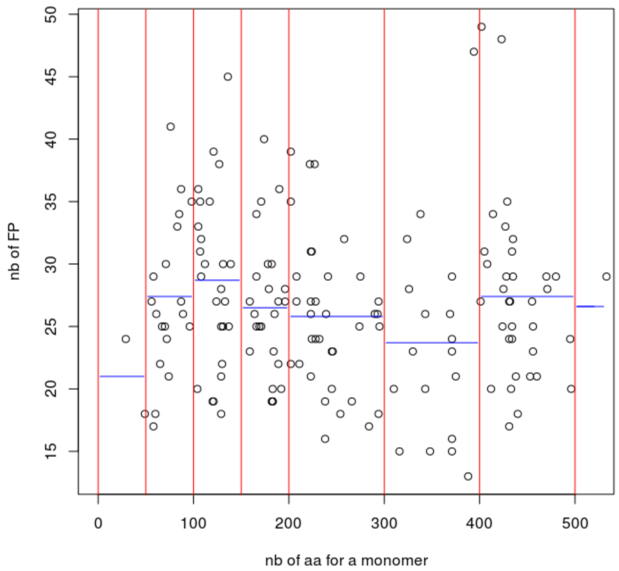

Figure S54: **Number of interaction partners for proteins of different sizes**. For each monomer in the Mintseris dataset, we report the number of protein partners with average IR ≤ 10. This is done on predictions (top) and knowing experimental interfaces (bottom). Red vertical bars indicate size intervals and blue horizontal bars indicate the mean of the distribution of average IRs for the proteins within a given size interval. The blue bars for the interval corresponding to proteins of sizes ≥ 500aa are computed on more proteins than shown in the plot. The protein's small size effect recognizable for predictions, disappears when experimental interfaces are used.

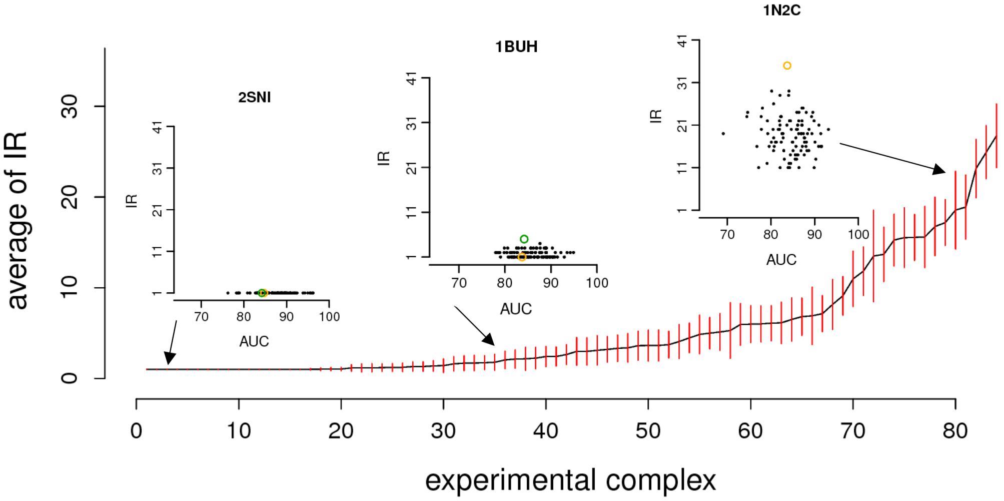

Figure S55: **Robustness of the native complex predictions with respect to the environment composition. Analysis based on knowledge of experimental interfaces**. Average Interaction Rank (IR) of the experimental partners computed over 100 random sets made of 40 proteins each (with error bars in red). The 84 complexes are ordered with respect to their increasing average IR value. For three of the 84 complexes (1BVN, 1BUH, 1N2C), detailed plots show the IR of the complex within each of the 100 random sets and the corresponding AUC value (black dots); green dots correspond to the IR of the complex computed over the Mintseris dataset; orange dots correspond to the IR of the complex computed over complexes in the same functional class. Compare to Figure 4 in the article.

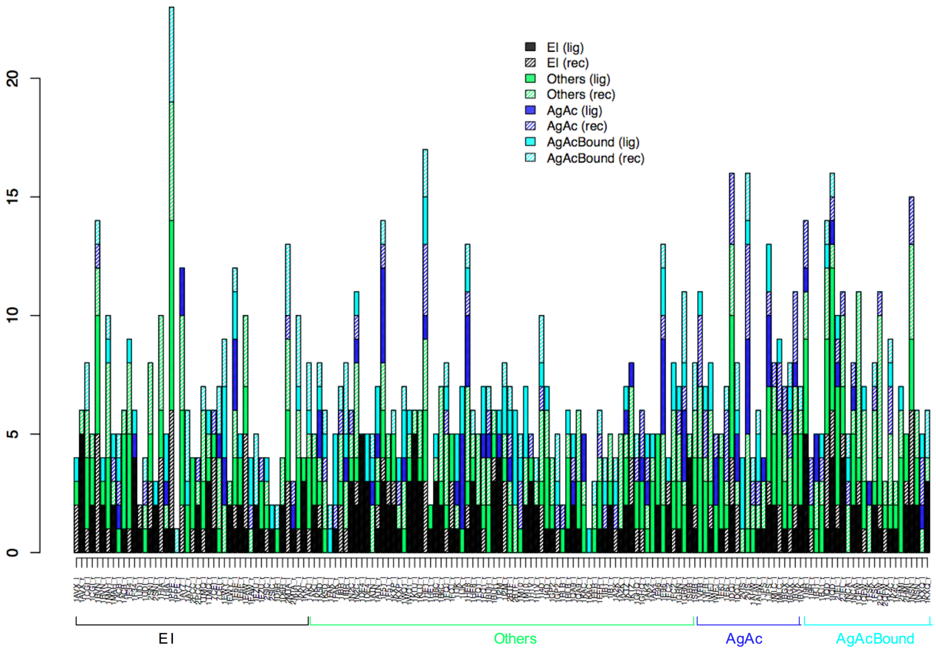

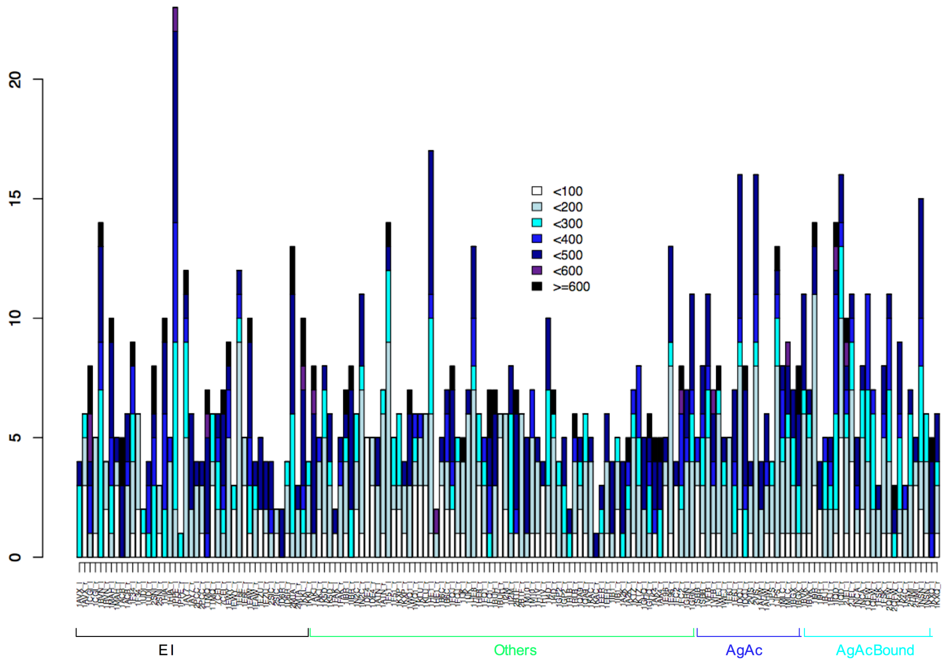

Figure S56: **Analysis of strongly interacting partners for the proteins in the Mintseris dataset**. Two analysis of the partners displaying strong interactions with the proteins in the Mintseris dataset (average IR < 5; see Fig. S38-S51) are reported. They describe the functional classes (top) and the sizes (bottom) of the partners for each monomer. The size of a partner is measured by the number of amino-acid of its sequence.

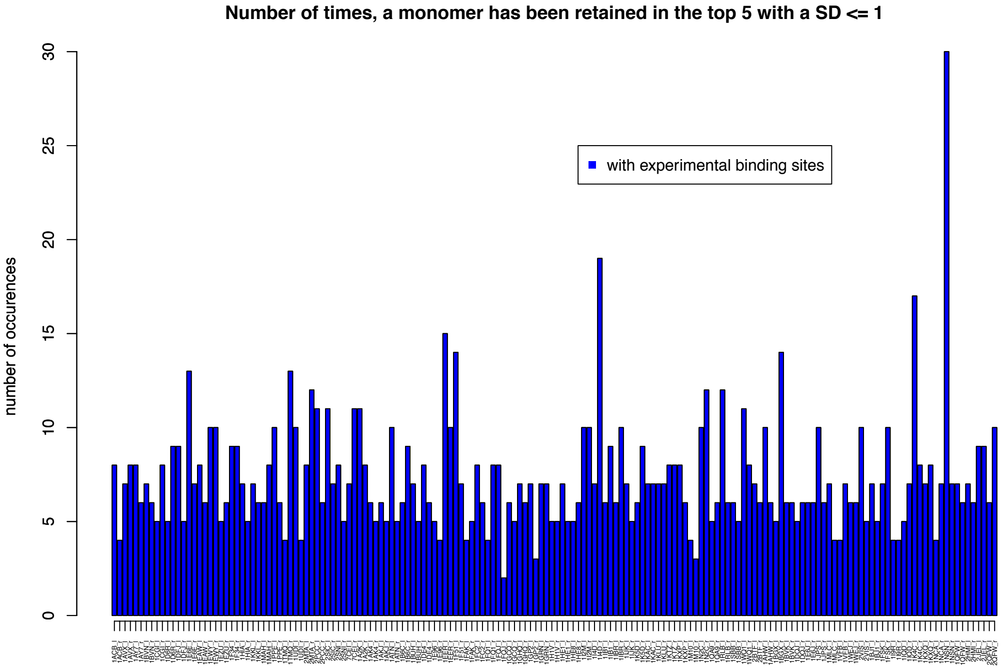

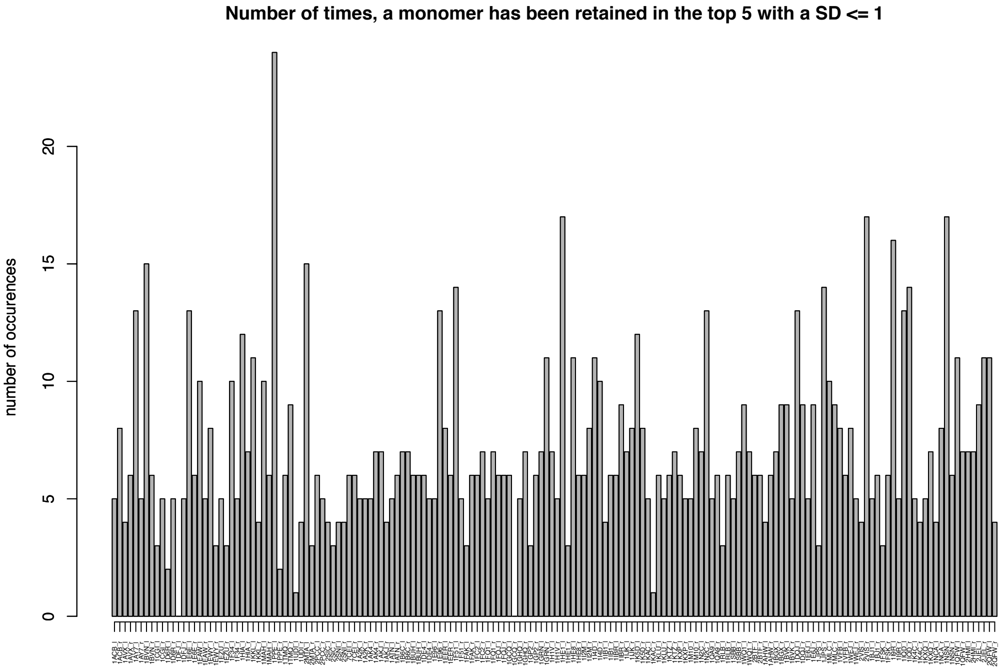

Figure S57: **Analysis of strongly interacting partners for the monomers in the Mintseris dataset**. Two analysis of the partners displaying strong interactions with the monomers in the Mintseris dataset (average IR < 5; see Fig. S38-S51) are reported for analyses based on the knowledge of experimental interfaces (top) and based on binding sites predictions (bottom).

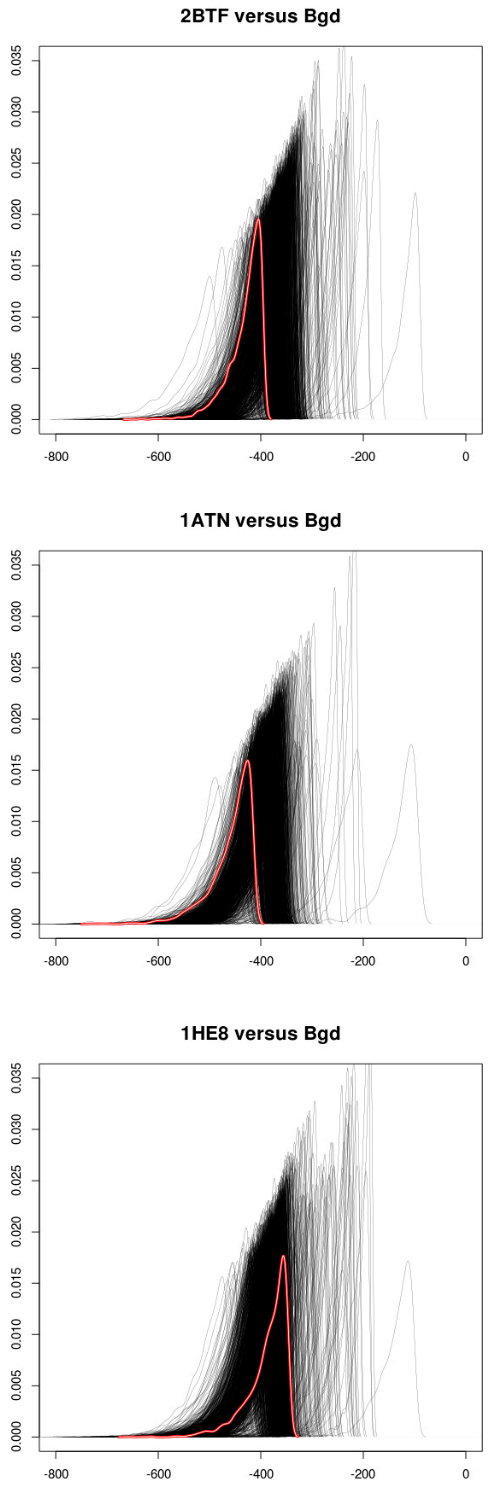

Figure S58: **HEX v6.3 analysis of proteins 2BTF_l, 1ATN_l, 1HE8_l**. HEX v6.3 was run on the three ligands of the complexes 2BTF, 1ATN, 1HE8 against a background of 922 proteins as done in (Wass M.N. et al. *Mol. Sys. Biol.* 2011). Direct comparison of these figures with Figures S5 and S10A in (Wass M.N. et al. *Mol. Sys. Biol.* 2011) shows that docking results are not reproducible.

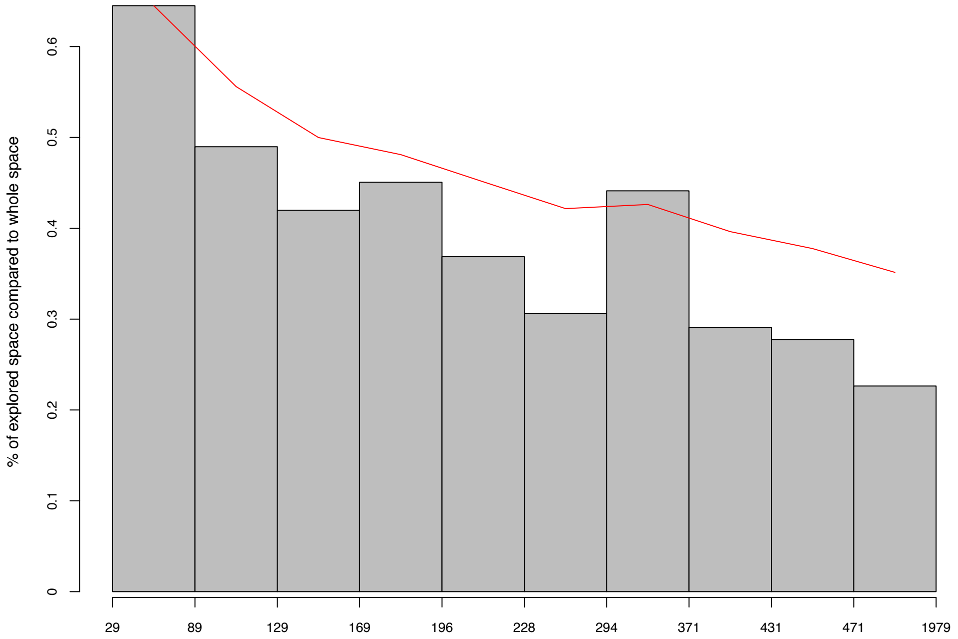

Figure S59: **Exploration of the restricted configuration space**. Diagram representing the proportion of explored configuration space compared to the whole space after the restriction based on JET. The set of proteins in the Mintseris dataset is split in 10 quantiles (bins) of 17 proteins each, selected by ordering the proteins with respect to their increasing sizes (x-axis). For each bin we computed the number of configurations for the proteins on their restricted configuration space and divide it by the total number of configurations for them. Then, each bin is associated to the percentage of the restricted configuration space (y-axis) computed as an average for all proteins in the bin. The cumulative average (from smaller to larger proteins) is given by the red curve (from left to right): the full set of 168 proteins corresponds to an average of 35% of the full space, small proteins exploration amounts to 64.5%, and large proteins exploration to 22.6%.

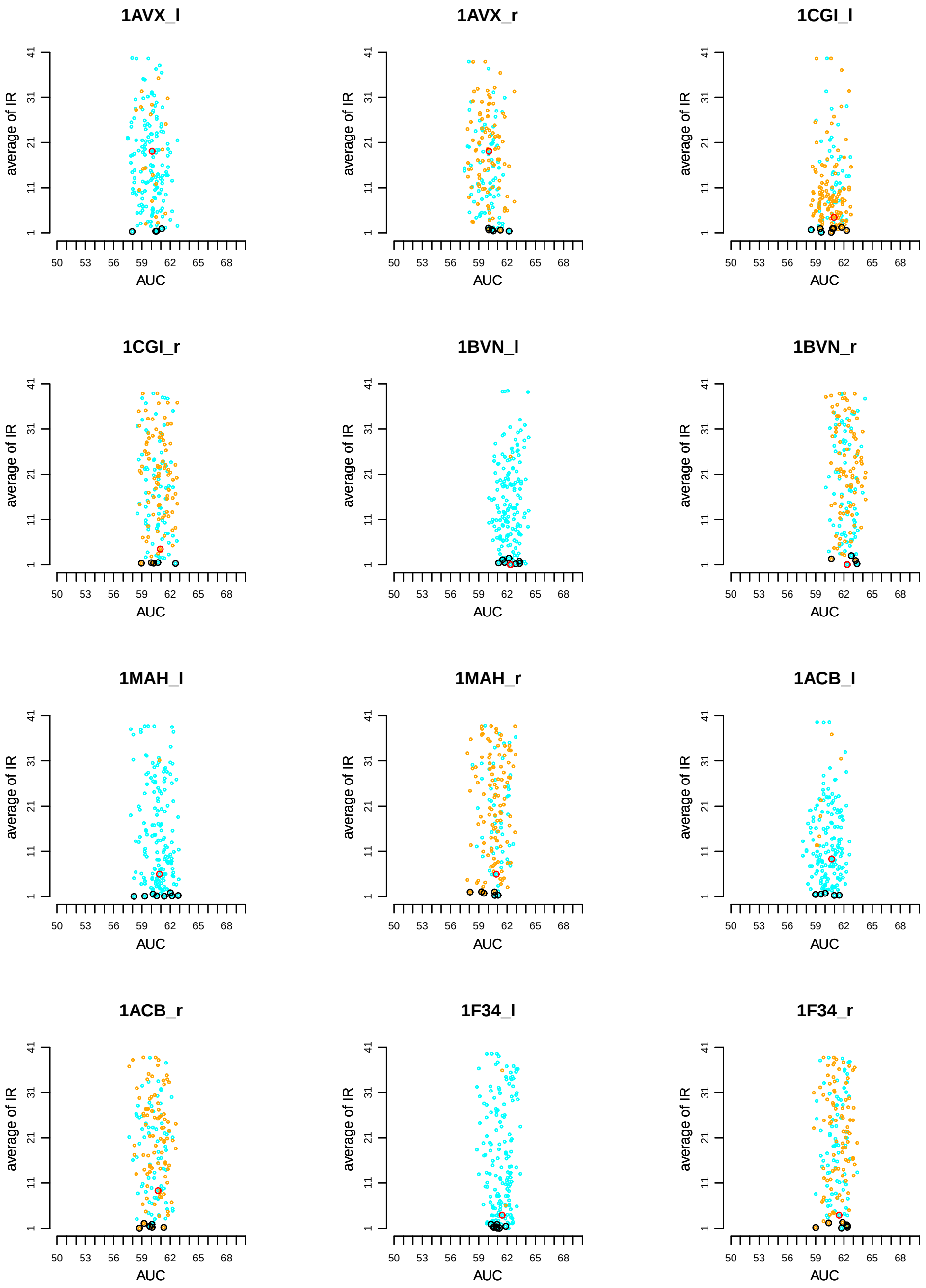

Figure S60: **Species information associated to the IR analysis of false complexes against the true one reported in Fig. S38-S51**. Each plot is associated to a protein structure P in the Mintseris dataset (see legend of Fig. S38 for plot's description - the IR analysis is based on predicted interfaces and the full exploration of the conformational space). Orange dots identify partners that have an homolog of the same species as P, and cyan dots identify the absence of a known homolog in that species. Homology has been computed based on 60% sequence identity (see Methods). A black contour line identifies bottom black dots in the plots of Fig. S38-S51. The red contour identifies the true interacting partner.

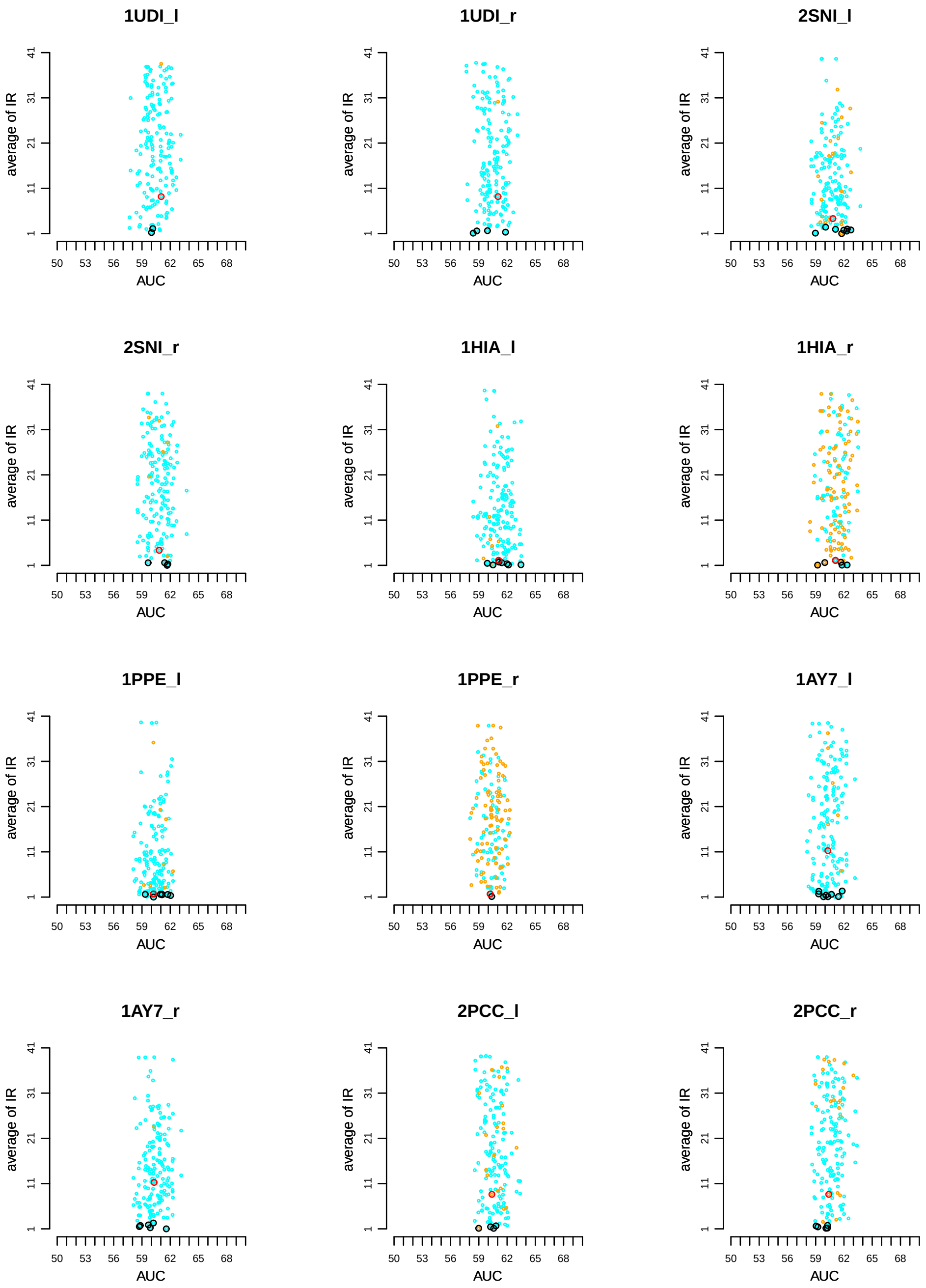

Figure S61: **Species information associated to the IR analysis of false complexes against the true one reported in Fig. S38-S51**. See legend in Fig. S60.

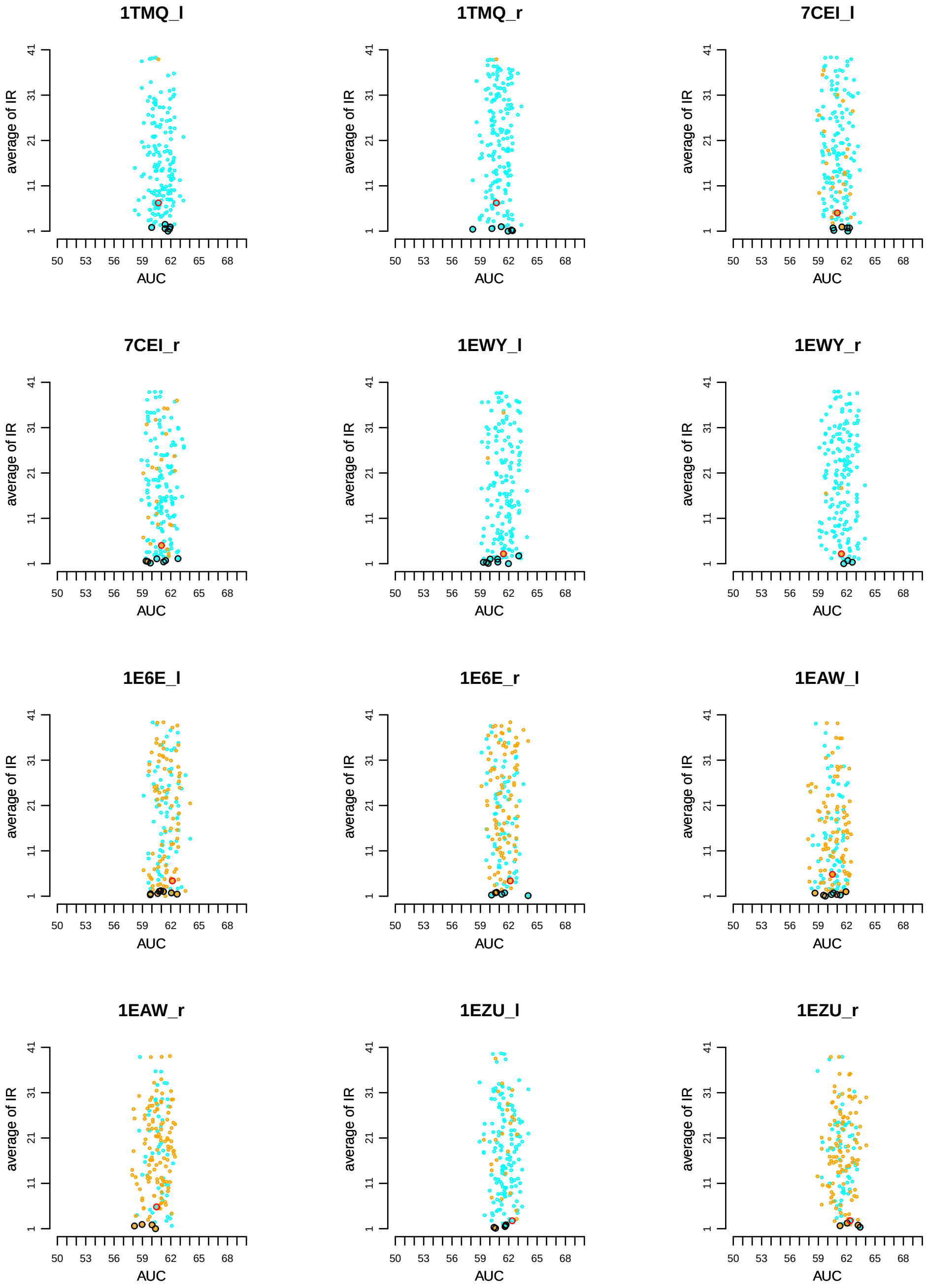

Figure S62: **Species information associated to the IR analysis of false complexes against the true one reported in Fig. S38-S51**. See legend in Fig. S60.

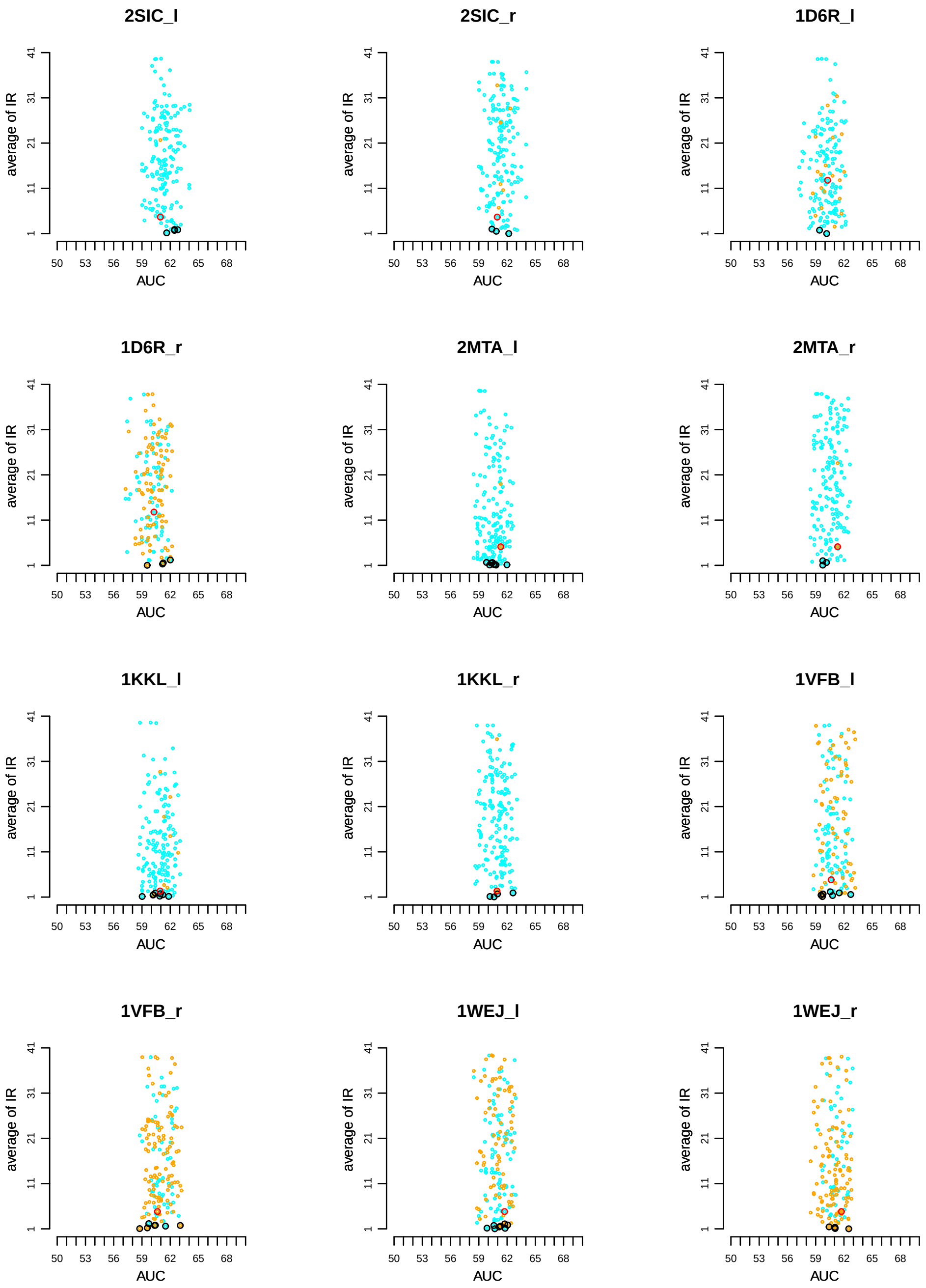

Figure S63: **Species information associated to the IR analysis of false complexes against the true one reported in Fig. S38-S51**. See legend in Fig. S60.

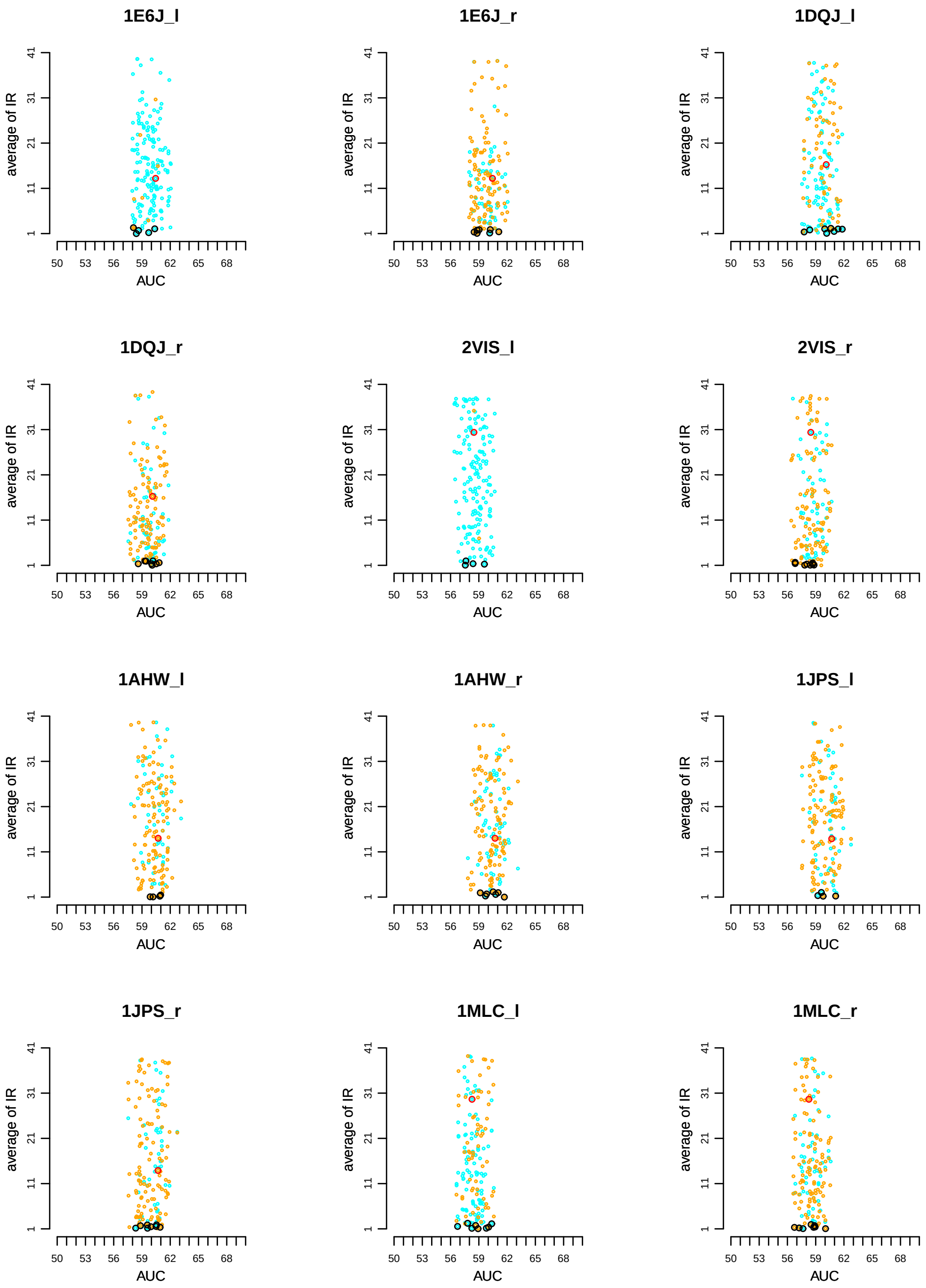

Figure S64: **Species information associated to the IR analysis of false complexes against the true one reported in Fig. S38-S51**. See legend in Fig. S60.

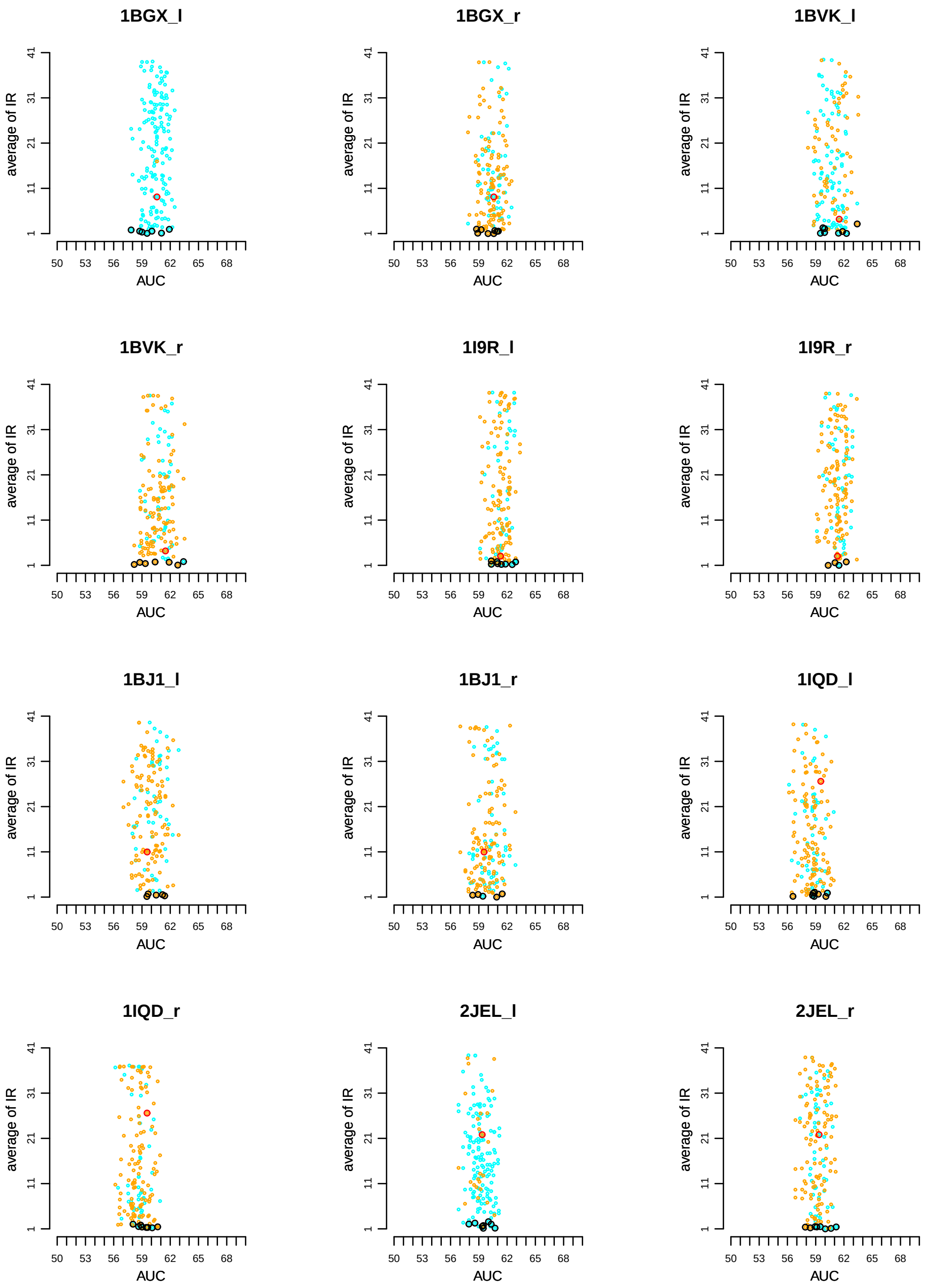

Figure S65: **Species information associated to the IR analysis of false complexes against the true one reported in Fig. S38-S51**. See legend in Fig. S60.

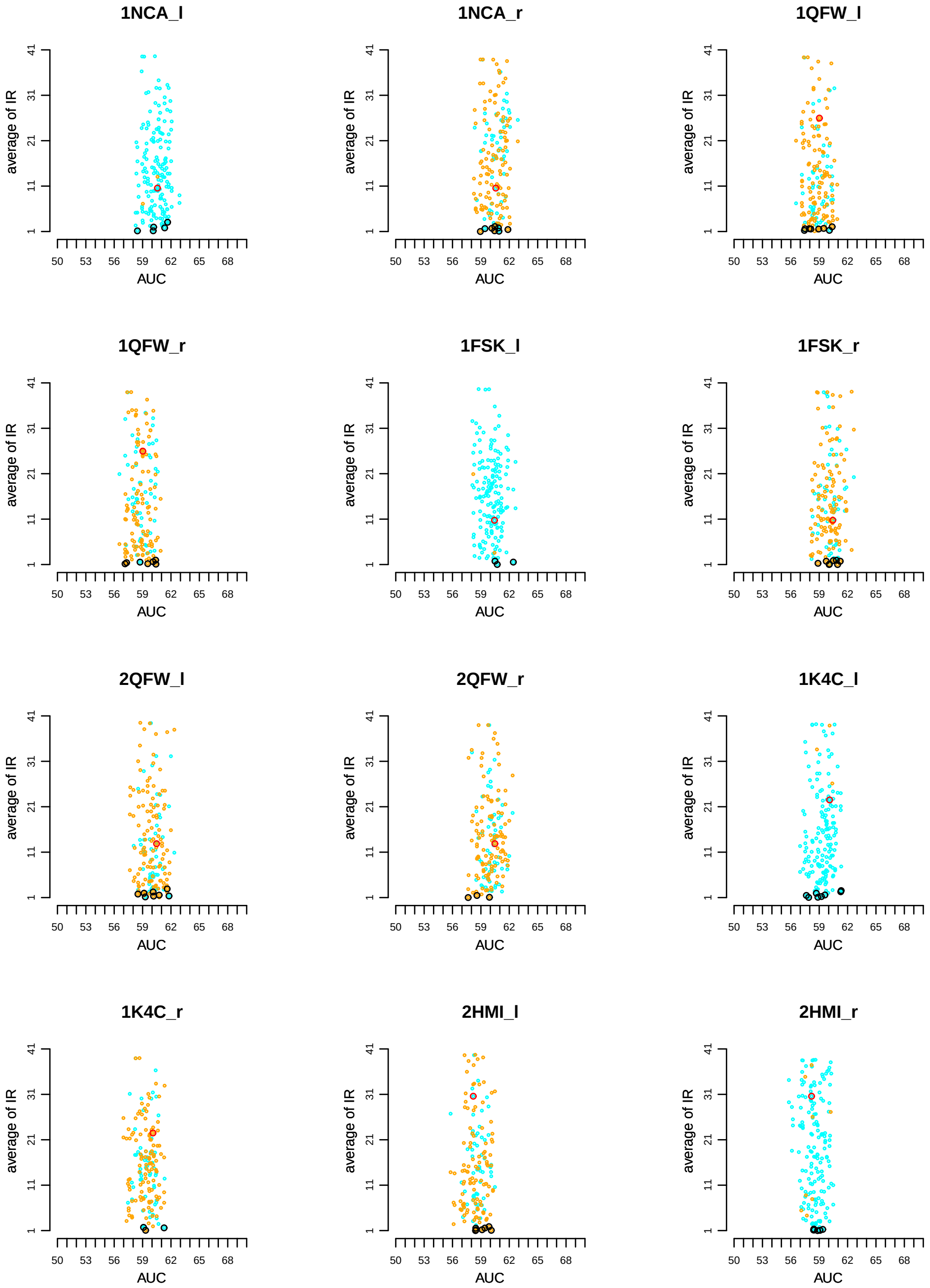

Figure S66: **Species information associated to the IR analysis of false complexes against the true one reported in Fig. S38-S51**. See legend in Fig. S60.

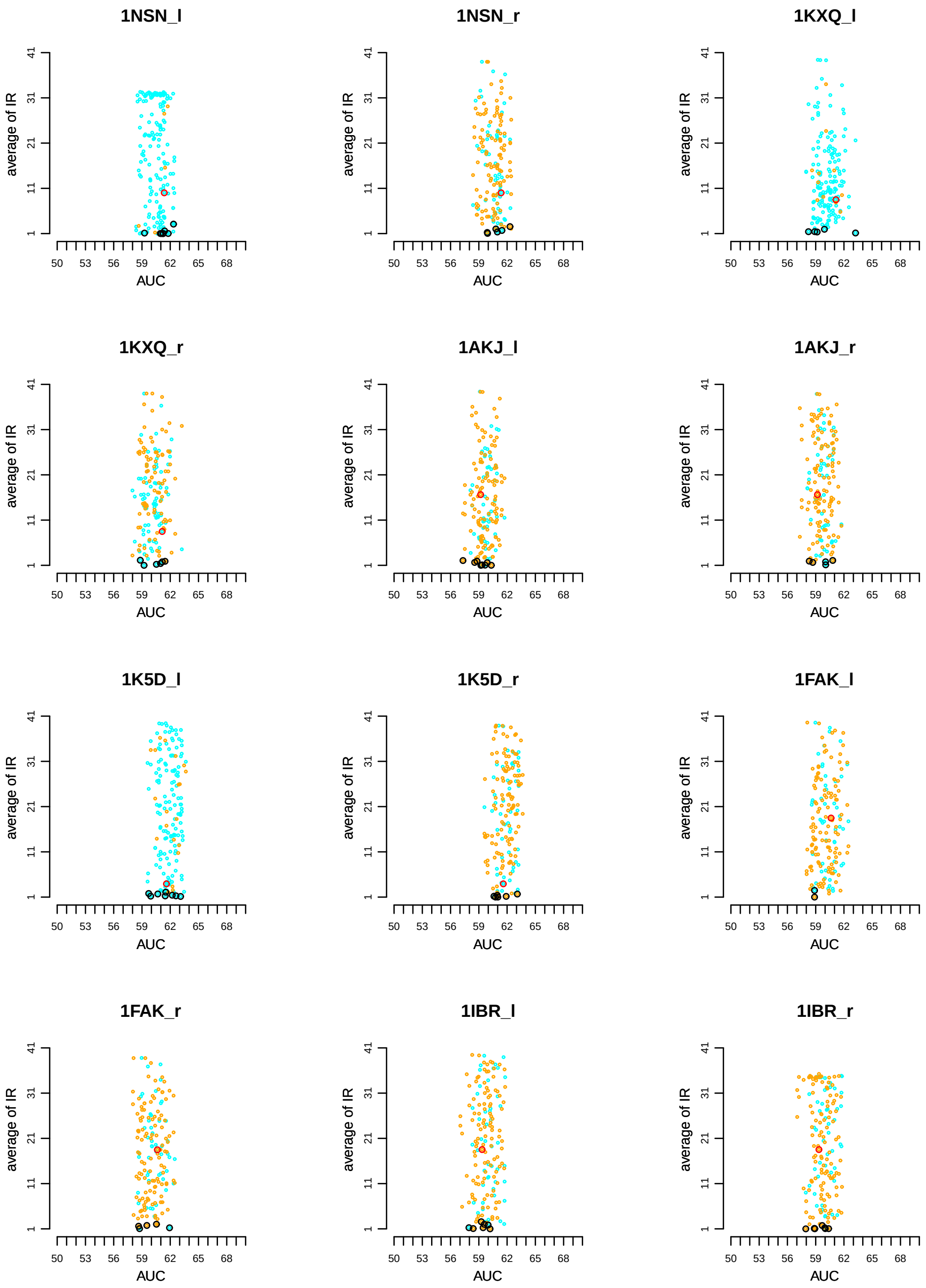

Figure S67: **Species information associated to the IR analysis of false complexes against the true one reported in Fig. S38-S51**. See legend in Fig. S60.

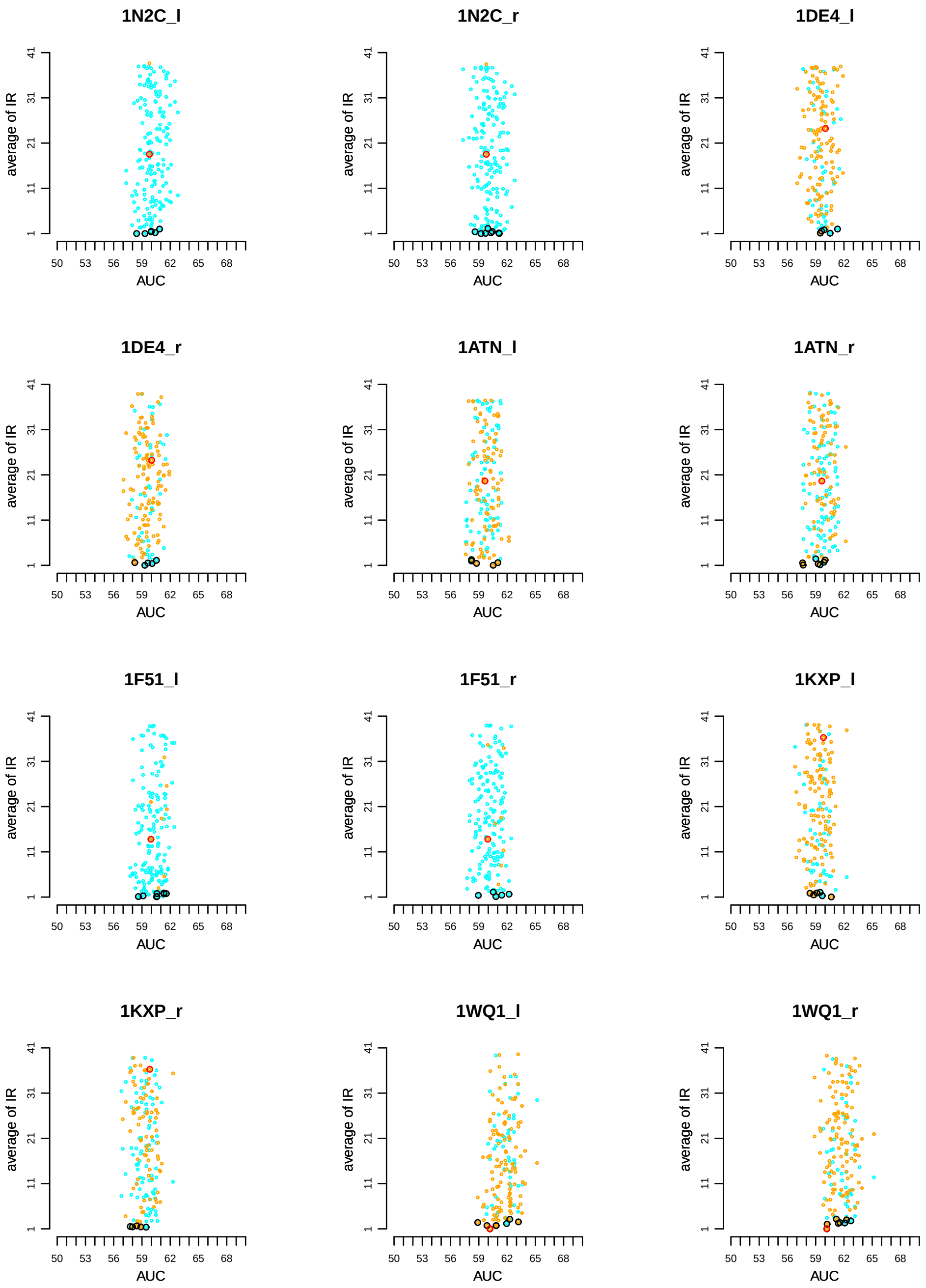

Figure S68: **Species information associated to the IR analysis of false complexes against the true one reported in Fig. S38-S51**. See legend in Fig. S60.

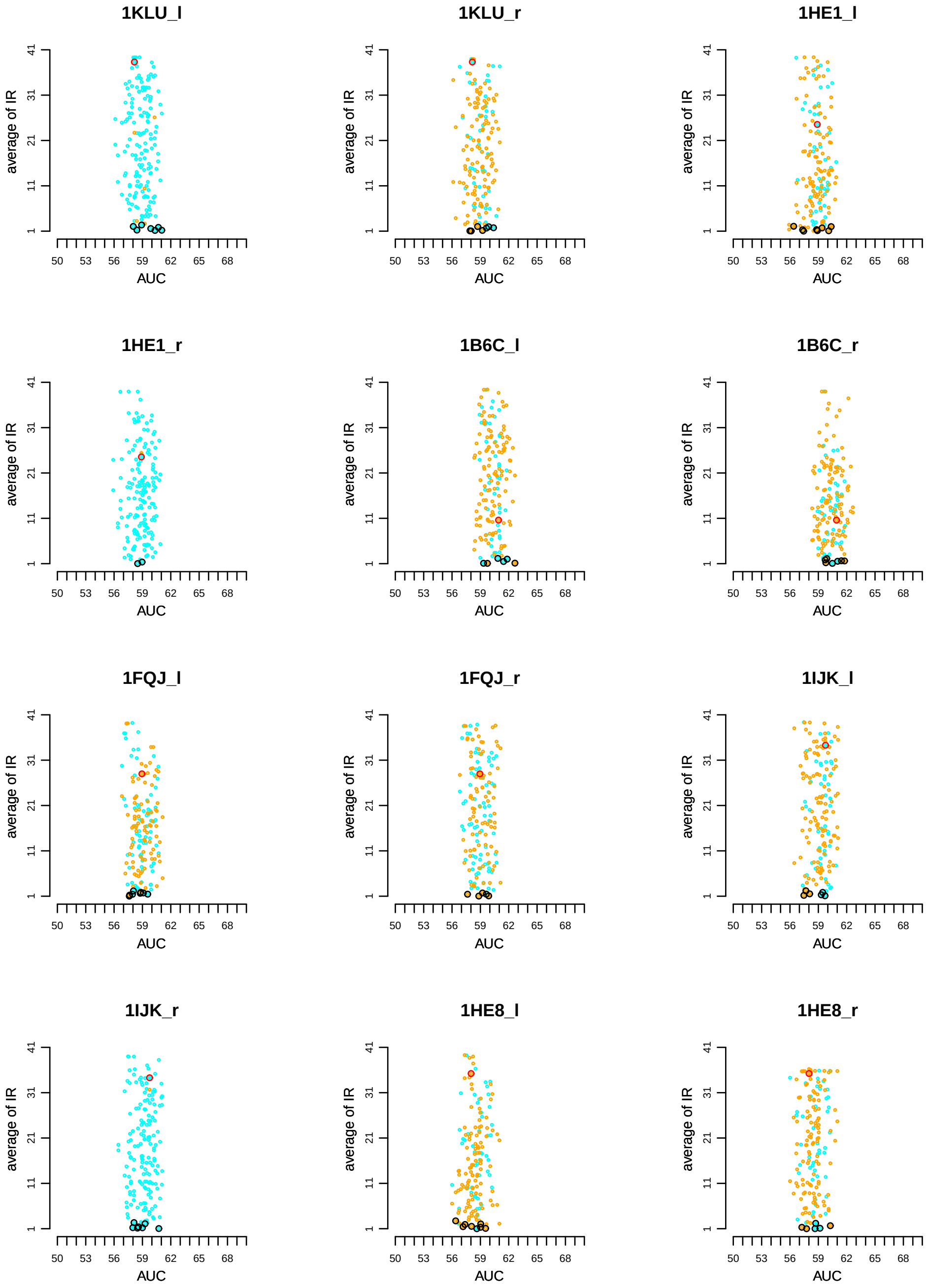

Figure S69: **Species information associated to the IR analysis of false complexes against the true one reported in Fig. S38-S51**. See legend in Fig. S60.

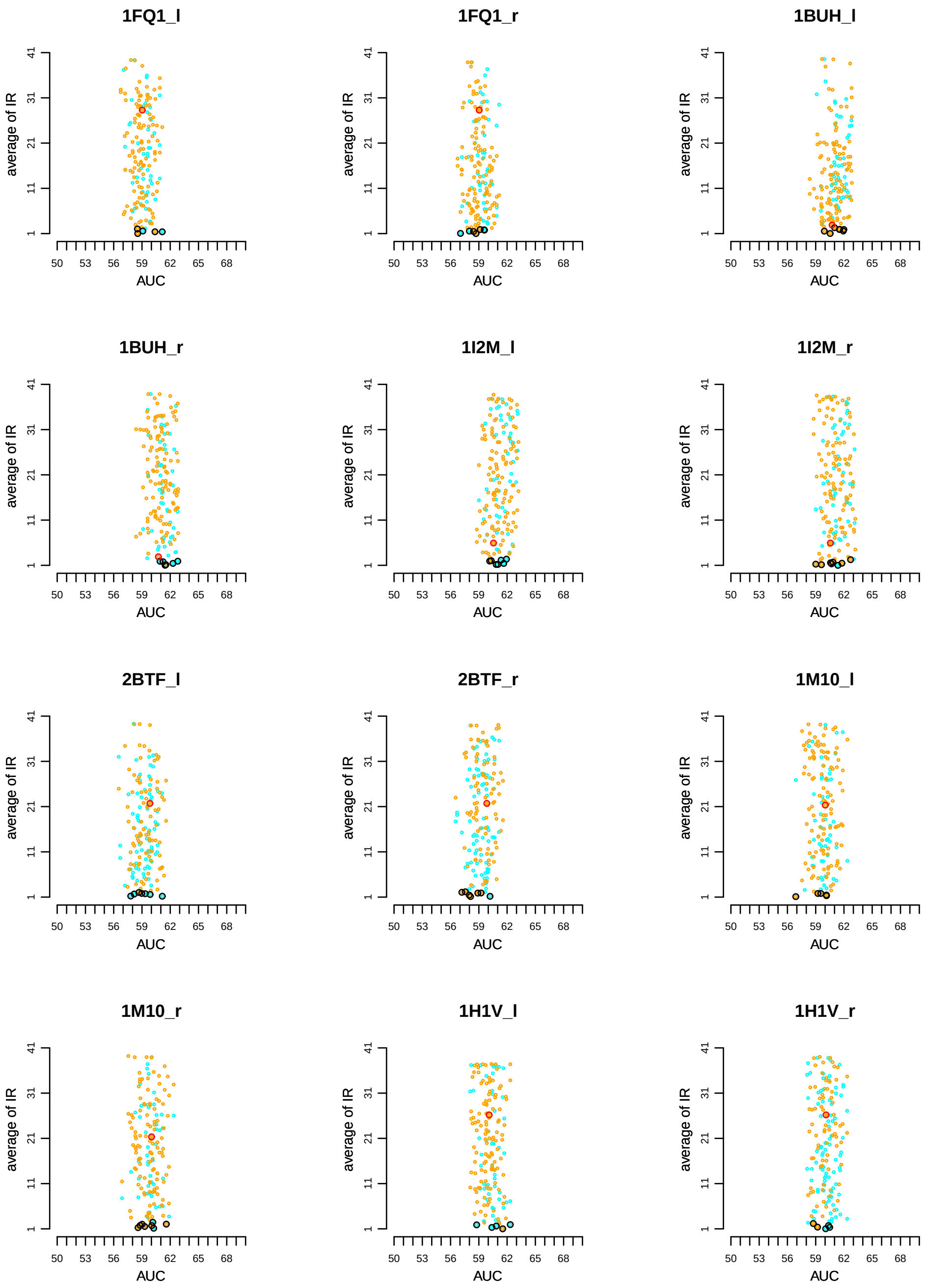

Figure S70: **Species information associated to the IR analysis of false complexes against the true one reported in Fig. S38-S51**. See legend in Fig. S60.

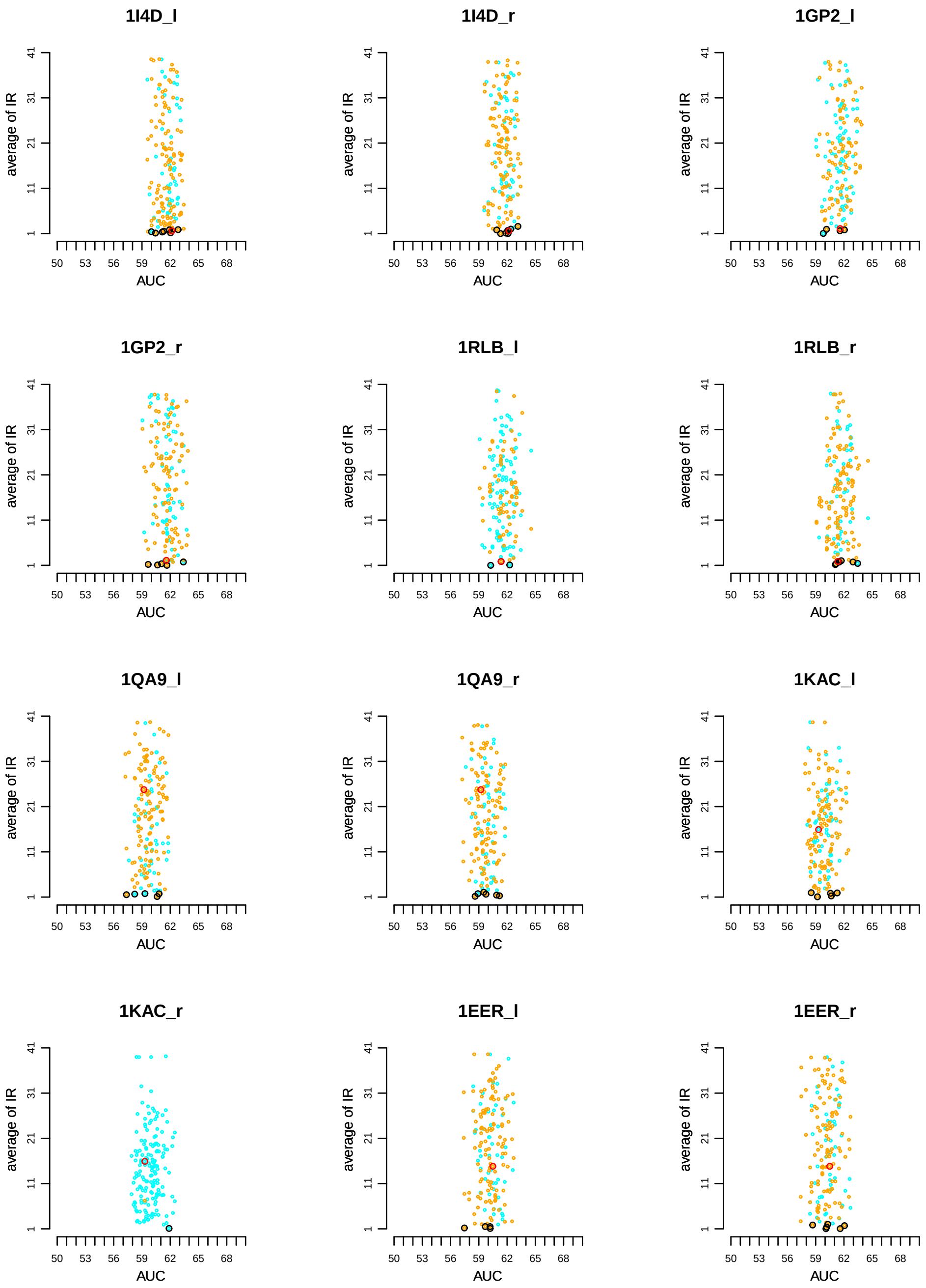

Figure S71: **Species information associated to the IR analysis of false complexes against the true one reported in Fig. S38-S51**. See legend in Fig. S60.

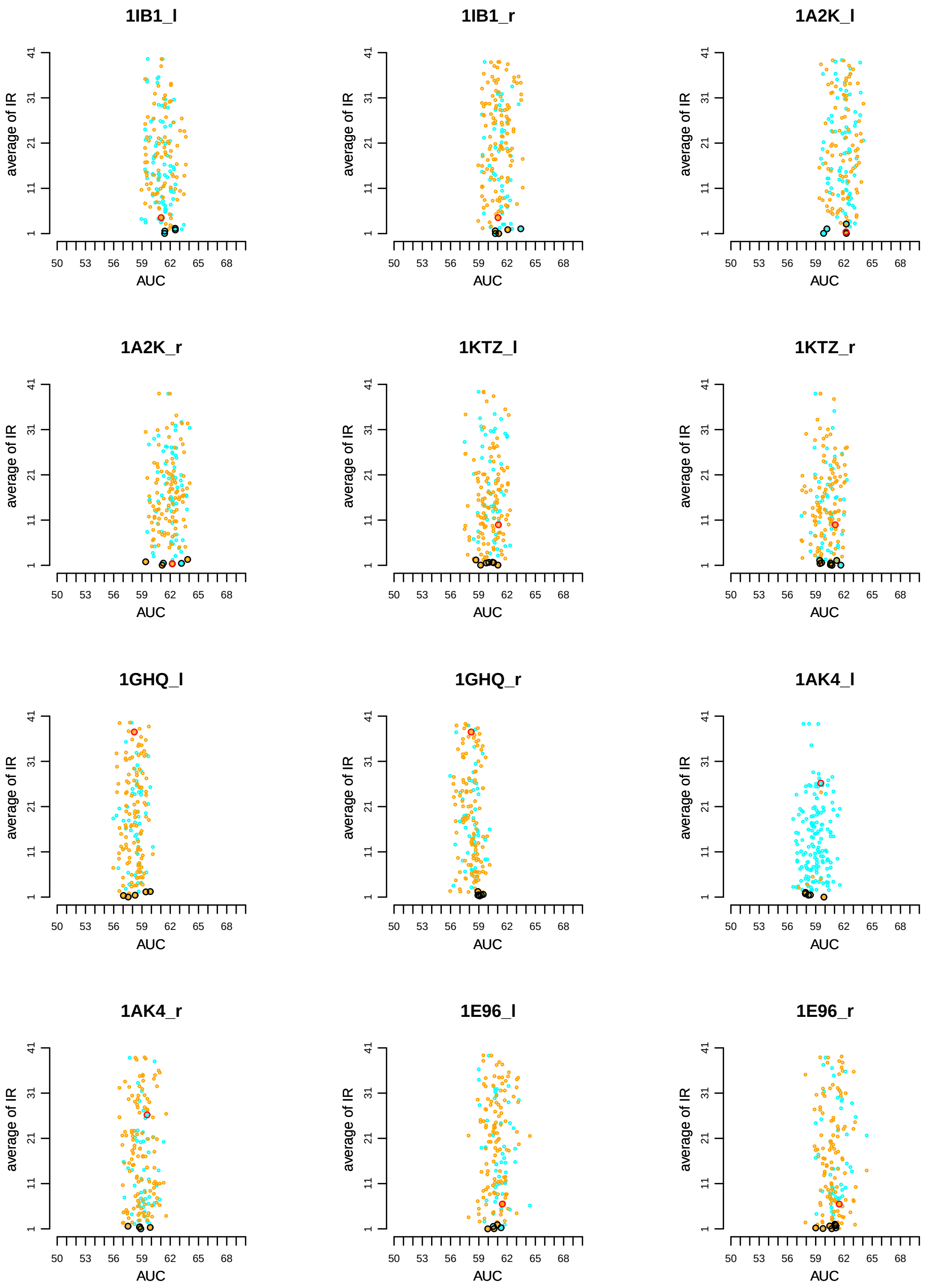

Figure S72: **Species information associated to the IR analysis of false complexes against the true one reported in Fig. S38-S51**. See legend in Fig. S60.

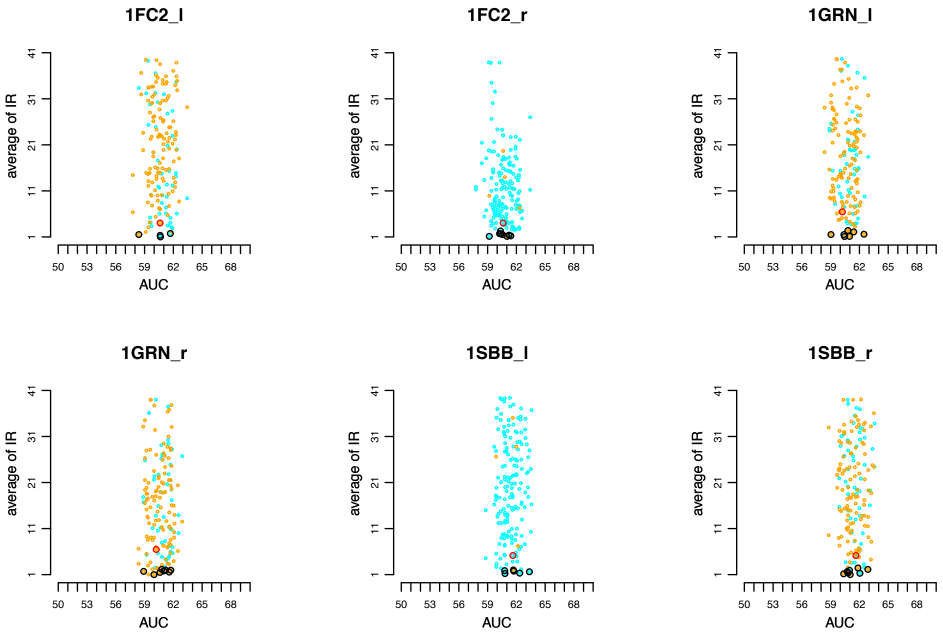

Figure S73: **Species information associated to the IR analysis of false complexes against the true one reported in Fig. S38-S51**. See legend in Fig. S60.

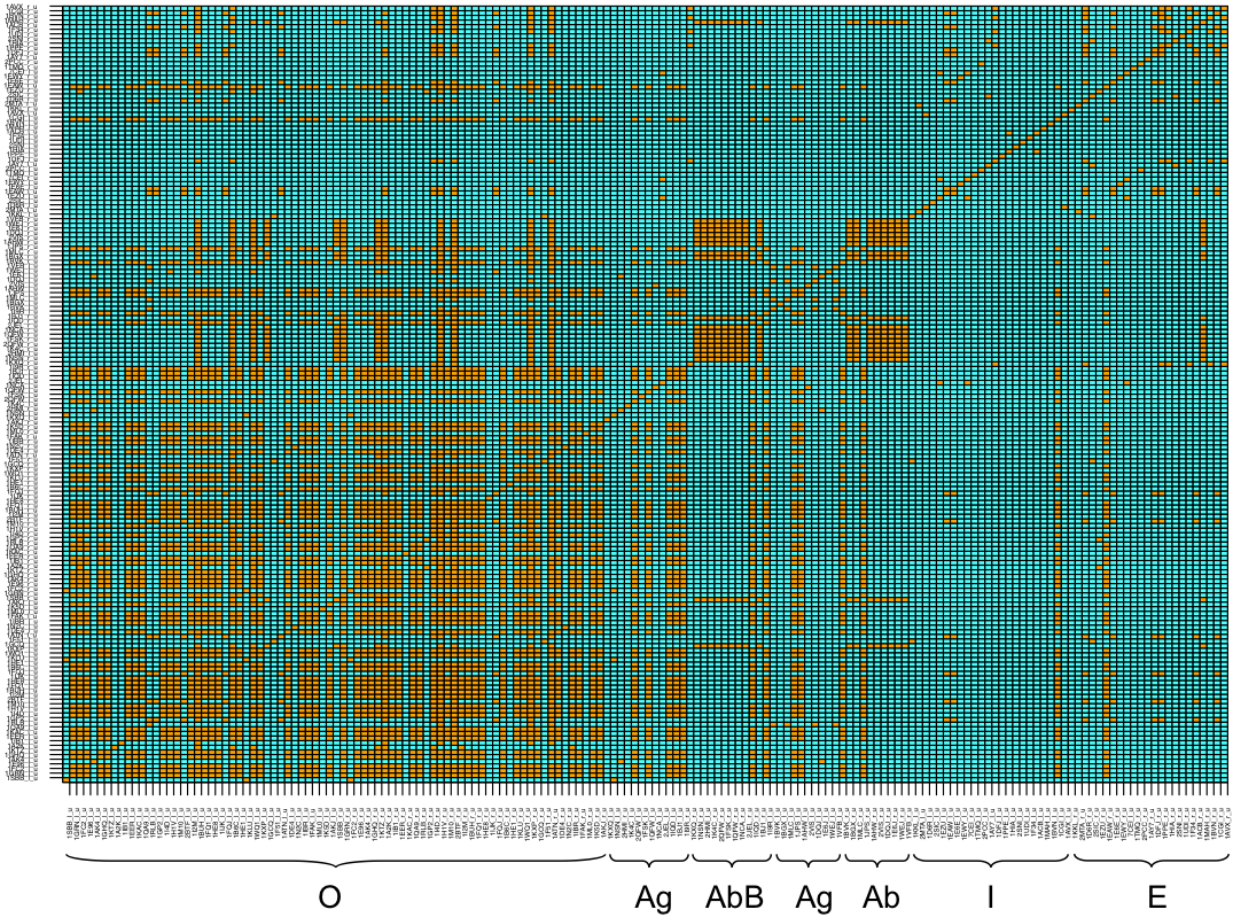

Figure S74: **Species represented in the Mintseris Benchmark 2.0 - homology defined at 100% of sequence identity**. Matrix reporting whether (orange) or not (cyan) any two protein structures of the Mintseris Benchmark 2.0 are represented by a common species at 100% sequence identity. Each line in the matrix represents a protein (see Methods). Proteins are ordered by classes: Others (O), Antibody (Ab), Bounded Antibody (AbB), Antigens (Ag), Inhibitors (I) and Enzymes (E). The y-axis follows the same order as the x-axis, from bottom to top. Compare to Fig. 7, Fig. S75 and Fig. S76.

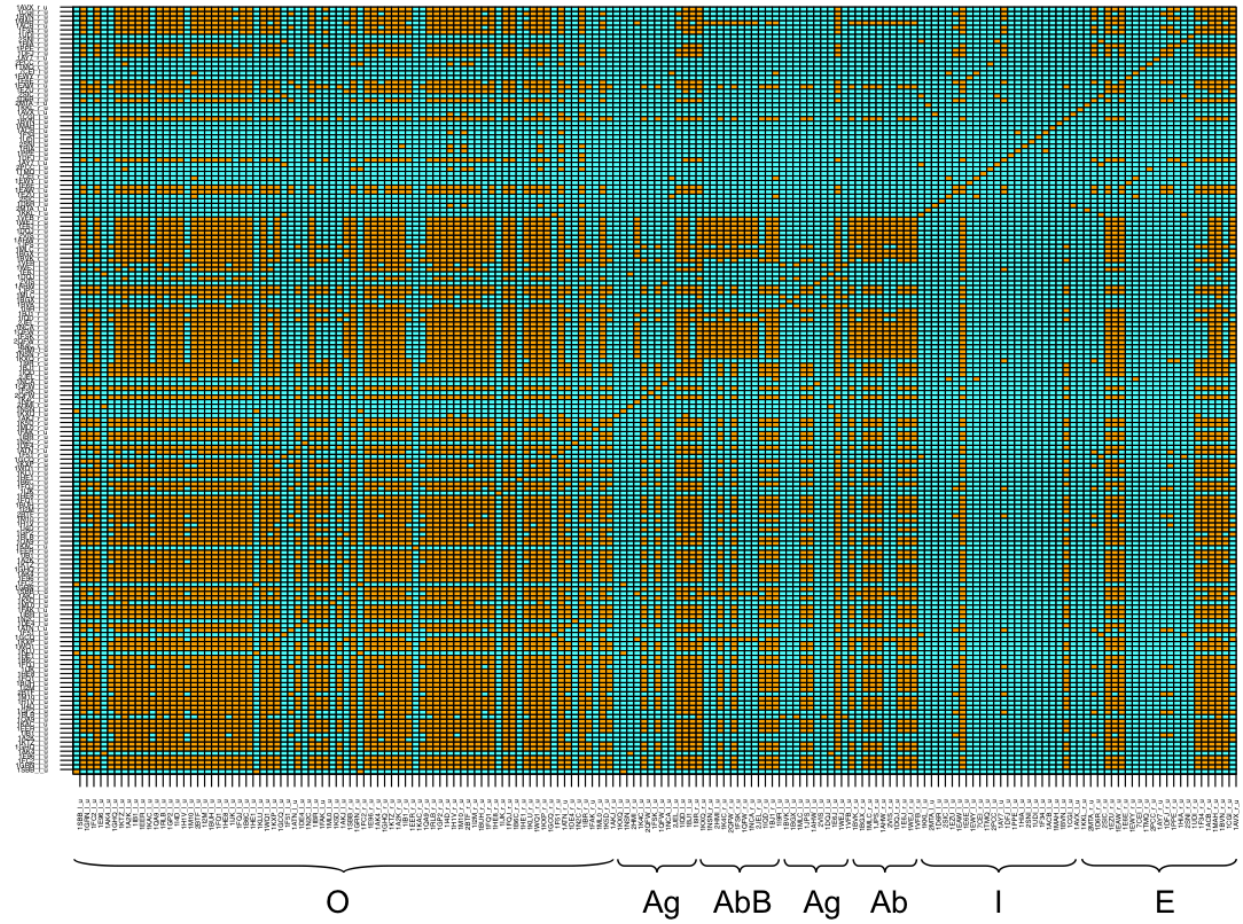

Figure S75: **Species represented in the Mintseris Benchmark 2.0 - homology defined at 80% of sequence identity**. Matrix reporting whether (orange) or not (cyan) any two protein structures of the Mintseris Benchmark 2.0 are represented by a common species at 80% sequence identity. Each line in the matrix represents a protein (see Methods). Proteins are ordered by classes: Others (O), Antibody (Ab), Bounded Antibody (AbB), Antigens (Ag), Inhibitors (I) and Enzymes (E). The y-axis follows the same order as the x-axis, from bottom to top. Compare to Fig. 7, Fig. S74 and Fig. S76.

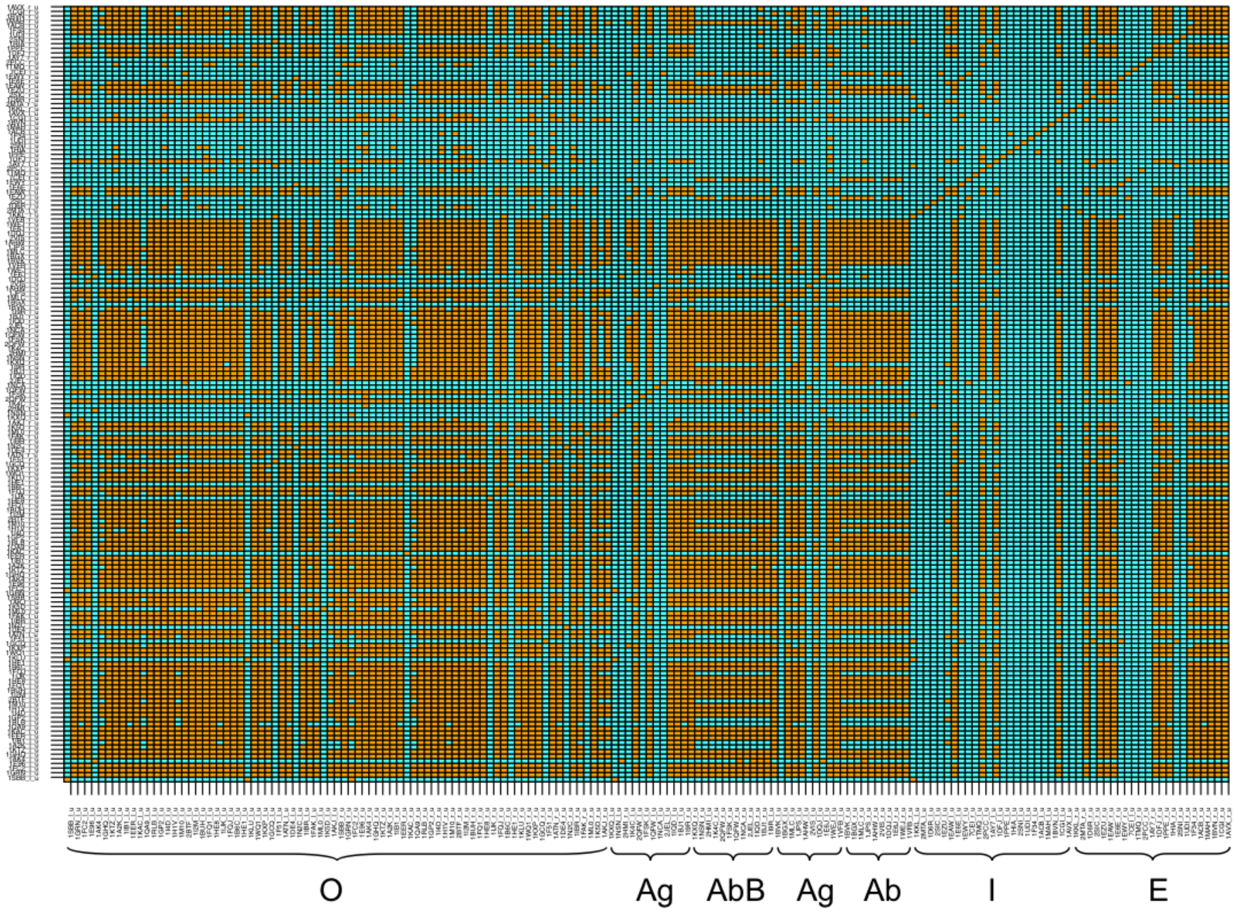

Figure S76: **Species represented in the Mintseris Benchmark 2.0 - homology defined at 60% of sequence identity**. Matrix reporting whether (orange) or not (cyan) any two protein structures of the Mintseris Benchmark 2.0 are represented by a common species at 80% sequence identity. Each line in the matrix represents a protein (see Methods). Proteins are ordered by classes: Others (O), Antibody (Ab), Bounded Antibody (AbB), Antigens (Ag), Inhibitors (I) and Enzymes (E). The y-axis follows the same order as the x-axis, from bottom to top. Compare to Fig. 7, Fig. S74 and Fig. S75.

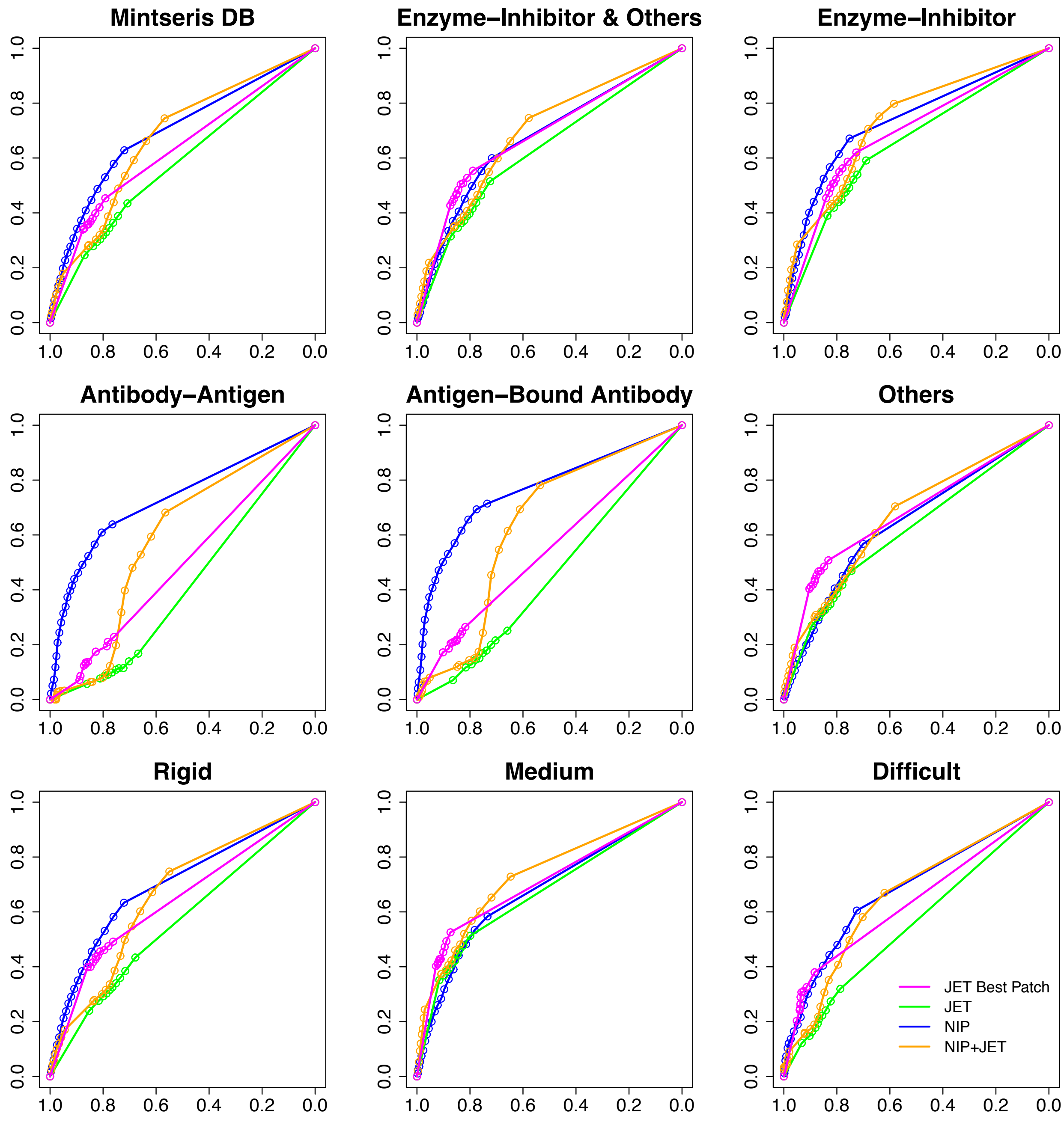

Figure S77: **ROC curves reporting the ability of NIP or JET to predict interface residues on the Mintseris dataset and its different subsets.** Specificity and sensitivity values are reported on the x-axis and y-axis respectively. Four scores are considered: JET Best Patch only, JET only, NIP only and NIP[0.4]+JET[0.6]. The JET Best Patch curve is obtained by considering only the patch that displays the highest coverage of the experimental interface's core, at each iteration of iJET. Score thresholds range from 0 to 1 by 0.05. See also SI Table S10.

IR analysis of true complexes							
**Protein dataset**		**Experimental interfaces**			**Predicted interfaces**		
**Subset type**	**#complexes**	**Max-Min**≤ 5	$\mu IR \leq 5$	$\mu IR \leq 10$	**Max-Min**≤ 5	$\mu IR \leq 5$	$\mu IR \leq 10$
Mintseris DB	84	41	56	69	14	24	41
Enzyme-Inhibitor	23	12	16	17	11	11	19
Antibody-Antigen	10	4	6	9	3	3	4
Antigen-Bound Antibody	12	7	8	11	1	1	3
Others	39	18	26	32	9	9	14

Table S1: **IR analysis of true complexes in the Mintseris Benchmark 2.0.** For the analysis described by the plots in Fig. S10-S16 (relative to experimental interfaces) and Fig. S17-S23 (relative to predicted interfaces) we report the number of complexes displaying a variation of 5 between the minimum and maximum IR value, the number of complexes with an average IR value ≤ 5 and the number of complexes with an average IR value ≤ 10. These values are reorganized with respect to functional classes of proteins (Enzyme-Inhibitor, Antibody-Antigen, Antigen-Bound Antibody and Others).

Complex numbering	Complex name	Average IR
1	1WQ1	1.0
2	1BVN	1.01
3	1A2K	1.39
4	1I4D	1.61
5	1RLB	1.62
6	1PPE	1.78
7	1HIA	2.22
8	1KKL	2.42
9	1GP2	2.61
10	1EZU	2.66
11	1EWY	2.84
12	1BUH	2.93
13	1K5D	3.27
14	1I9R	3.49
15	1FC2	3.76
16	1F34	3.82
17	2SNI	4.12
18	1VFB	4.28
19	2SIC	4.42
20	1WEJ	4.44
21	1BVK	4.45
22	1IB1	4.46
23	1CGI	4.64
24	1E6E	4.77
25	1EAW	5.03
26	1SBB	5.13
27	7CEI	5.26
28	2MTA	5.34
29	1MAH	5.83
30	1I2M	5.87
31	1E96	6.19
32	1GRN	6.62
33	1TMQ	7.48
34	1KXQ	8.12
35	2PCC	8.63
36	1ACB	8.66
37	1BGX	8.98
38	1UDI	9.02
39	1NSN	9.77
40	1KTZ	9.81
41	1AY7	9.95
42	1B6C	10.42
43	1BJ1	10.46
44	1NCA	10.51
45	1FSK	11.11
46	1D6R	12.55
47	1F51	12.8
48	1AHW	12.88
49	2QFW	13.19
50	1E6J	13.38
51	1JPS	13.41
52	1EER	13.8
53	1DQJ	15.74
54	1AKJ	16.26
55	1KAC	17.29
56	1FAK	17.67
57	1N2C	18.1
58	1IBR	18.72
59	1AVX	18.77
60	1ATN	20.07
61	2BTF	21.17
62	2JEL	21.34
63	1M10	21.54
64	1K4C	22.41
65	1DE4	23.49
66	1QA9	23.51
67	1HE1	23.89
68	1QFW	25.74
69	1IQD	25.98
70	1AK4	26.1
71	1H1V	26.39
72	1FQ1	27.54
73	1FQJ	27.83
74	1MLC	29.11
75	2HMI	30.07
76	2VIS	30.38
77	1IJK	33.84
78	1HE8	35.04
79	1KXP	36.06
80	1GHQ	37.41
81	1KLU	38.1
82	1DFJ	38.73
83	1ML0	39.19
84	1GCQ	39.47

Table S2: **Coordinates of Figure 4** The complex numbering used in Figure 4 is reported with the name of the complex and the average rank.

**PDB**	**Species**	**100%**		**80%**		**60%**		**PDB - partners**
		#homo	ratio	#homo	ratio	#homo	ratio	
1AVX_r	*Sus_scrofa*	15	0.00	67	0.33	88	0.33	1E6E_l 1I2M_r
1CGI_r	*Bos_taurus*	21	0.00	69	0.60	90	0.60	1HE1_l 1HE8_l 1KTZ_r
1BVN_r	*Sus_scrofa*	15	0.00	67	0.00	88	0.40	1EAW_l 1GHQ_l
1MAH_r	*Mus_musculus*	30	0.33	82	0.50	111	0.67	1CGI_l 1MLC_r 1IQD_l 1GCQ_l
1ACB_r	*Bos_taurus*	21	0.00	69	0.67	90	0.67	1IBR_l 1KXP_l 1HE1_l 1I4D_l
1F34_r	*Sus_scrofa*	15	0.00	67	0.29	88	0.57	1EAW_l 1WEJ_l 1IQD_l 1ML0_l
1UDI_r	*Human_herpesvirus_1*	1	0.00	1	0.00	1	0.00	
2SNI_r	*Bacillus_amyloliquefaciens*	3	0.00	5	0.00	6	0.00	
1HIA_r	*Sus_scrofa*	15	0.00	67	0.33	88	0.50	1WEJ_l 1IQD_l 1FQJ_r
1PPE_r	*Bos_taurus*	21	0.00	69	0.00	90	0.00	
1DFJ_r	*Bos_taurus*	21	0.20	69	0.20	90	0.20	1E6E_l
1AY7_r	*Streptomyces_aureofaciens*	1	0.00	1	0.00	1	0.00	
2PCC_r	*Saccharomyces_cerevisiae*	2	0.00	11	0.00	20	0.00	
1TMQ_r	*Tenebrio_molitor*	1	0.00	1	0.00	1	0.00	
7CEI_l	*Escherichia_coli*	4	0.00	6	0.00	25	0.17	1JPS_r
1EWY_r	*Nostoc_sp.*	2	0.00	2	0.00	3	0.00	
1E6E_r	*Bos_taurus*	21	0.00	69	0.33	90	0.33	1GCQ_l 1HE1_l
1EAW_r	*Homo_sapiens*	67	0.75	94	0.75	118	1.00	1BGX_r 1QFW_l 2QFW_l 1GHQ_r
1EZU_r	*Rattus_norvegicus*	11	0.25	61	0.50	103	0.75	1DQJ_r 1I4D_l 1FC2_l
2SIC_r	*Bacillus_amyloliquefaciens*	3	0.00	5	0.00	6	0.00	
1D6R_r	*Bos_taurus*	21	0.00	69	0.50	90	0.50	1IBR_r 1I2M_r
2MTA_r	*Paracoccus_denitrificans*	2	0.00	2	0.00	2	0.00	
1KKL_r	*Lactobacillus_casei*	1	0.00	1	0.00	2	0.00	
1AVX_l	*Glycine_max*	2	0.00	7	0.00	19	0.00	
1CGI_l	*Homo_sapiens*	67	0.50	94	0.75	118	0.75	1MAH_r 1I9R_r 1K5D_r 1FAK_r 1DE4_l 1IB1_l
1BVN_l	*Streptomyces_tendae*	1	0.00	1	0.00	1	0.00	
1MAH_l	*Dendroaspis_angusticeps*	1	0.00	1	0.00	1	0.00	
1ACB_l	*Hirudo_medicinalis*	2	0.00	6	0.00	7	0.00	
1F34_l	*Ascaris_suum*	1	0.00	1	0.00	2	0.00	
1UDI_l	*Bacillus_phage_PBS2*	1	0.00	1	0.00	1	0.00	
2SNI_l	*Hordeum_vulgare*	1	0.00	7	0.00	19	0.12	1BUH_r
1HIA_l	*Hirudo_medicinalis*	2	0.00	6	0.09	7	0.09	1H1V_r
1PPE_l	*Cucurbita_maxima*	1	0.00	2	0.00	8	0.00	
1DFJ_l	*Sus_scrofa*	15	0.00	67	0.00	88	0.00	
1AY7_l	*Bacillus_amyloliquefaciens*	3	0.00	5	0.00	6	0.00	
2PCC_l	*Saccharomyces_cerevisiae*	2	0.00	11	0.00	20	0.25	1HE8_l
1TMQ_l	*Eleusine_coracana*	1	0.00	1	0.00	1	0.00	
7CEI_r	*Escherichia_coli*	4	0.00	6	0.00	25	0.14	1IQD_r
1EWY_l	*Nostoc_sp.*	2	0.00	2	0.00	3	0.00	
1E6E_l	*Bos_taurus*	21	0.15	69	0.38	90	0.69	1AVX_r 1DFJ_r 1DQJ_l 1MLC_l 1BVK_l 2QFW_l 1FQJ_r 1EER_r 1GRN_l
1EAW_l	*Bos_taurus*	21	0.00	69	0.50	90	0.60	1BVN_r 1F34_r 1KXP_l 1KLU_r 1EER_l 1SBB_r
1EZU_l	*Escherichia_coli*	4	0.00	6	0.00	25	0.40	1IQD_r 2JEL_r
2SIC_l	*Streptomyces_albogriseolus*	1	0.00	1	0.00	1	0.00	
1D6R_l	*Glycine_max*	2	0.00	7	0.00	19	0.00	
2MTA_l	*Paracoccus_denitrificans*	2	0.00	2	0.00	2	0.00	
1KKL_l	*Bacillus_subtilis*	4	0.00	5	0.00	8	0.10	1B6C_r
1VFB_r	*Mus_musculus*	30	0.29	82	0.57	111	0.57	1E6J_r 2VIS_r 1HE1_l 1GP2_r
1WEJ_r	*Mus_musculus*	30	0.00	82	0.40	111	1.00	1DQJ_l 1JPS_r 1BVK_l 1M10_l 1IB1_l

Table S3: **Analysis of species representation among proteins of the Mintseris dataset.** For each protein structure P in the Mintseris dataset we report: the name of the protein structure (PDB), its species, two values for each one of the three analysis realized by considering homology at 100%, 80% and 60% sequence identity for the partners of P (see Methods). The first value (#homo) corresponds to the number of partners in the database that have a homolog of the same species, at X% sequence identity from P, where X can be 100, 80 or 60. This value corresponds to the number of orange dots in the plots of Fig. S60-S73. The second number (ratio) is the proportion of orange dots among the black dots, that is the most likely partners of P. The last column lists the names of the protein partners among the most likely ones (black dots) that have an homolog of the same species as P.

PDB	Species	100%		80%		60%		PDB - partners
		#homo	ratio	#homo	ratio	#homo	ratio	
1E6J_r	*Mus_musculus*	30	0.43	82	0.86	111	0.86	1VFB_r 2VIS_r 1JPS_r 1NCA_r 1K5D_r 1HE8_l
1DQJ_r	*Mus_musculus*	30	0.25	82	0.50	111	0.75	1EZU_r 1AHW_l 1I9R_l 2JEL_r 1FSK_r 1FAK_r
2VIS_r	*Mus_musculus*	30	0.44	82	0.62	111	1.00	1VFB_l 1VFB_r 1E6J_r 1DQJ_l 1MLC_l 1MLC_r 1BGX_r 1BVK_l 1I9R_r 1BJ1_r 1K4C_l 1KXP_r 1HE1_l 1EER_r 1E96_r 1GRN_r
1AHW_r	*Mus_musculus*	30	0.29	82	0.43	111	0.71	1BGX_r 1BJ1_r 1KXQ_l 1ML0_l 1GP2_r
1JPS_r	*Homo_sapiens*	67	0.23	94	0.31	118	0.54	1D6R_r 1VFB_l 1WEJ_r 1DQJ_l 1M10_l 1GHQ_l 1GRN_l
1MLC_r	*Mus_musculus*	30	0.44	82	0.67	111	0.78	1MAH_r 1DQJ_r 2VIS_r 1KXQ_l 1BUH_l 2BTF_l 1I4D_l
1BGX_r	*Mus_musculus*	30	0.25	82	0.75	111	0.88	1EAW_r 2VIS_r 1AHW_r 1I9R_r 1IQD_l 1H1V_l 1GHQ_r
1BVK_r	*Homo_sapiens*	67	0.57	94	0.71	118	0.86	2PCC_l 1IQD_r 1FAK_l 1HE1_l 1RLB_l 1GHQ_r
1VFB_l	*Gallus_gallus*	9	0.00	41	0.27	65	0.45	1BJ1_l 1ATN_l 1HE8_l 1E96_r 1GRN_r
1WEJ_l	*Equus_caballus*	12	0.00	57	0.38	78	0.50	1HIA_r 1BJ1_l 1E96_r 1GRN_r
1E6J_l	*HIV_1*	3	0.20	3	0.20	8	0.20	1AK4_l
1DQJ_l	*Gallus_gallus*	9	0.00	41	0.25	65	0.31	1E6E_l 1HE8_l 1I2M_l 1KTZ_l 1E96_r
2VIS_l	*Influenza_A_virus*	2	0.00	2	0.00	2	0.00	
1AHW_l	*Homo_sapiens*	67	0.50	94	0.50	118	1.00	1DQJ_r 1NCA_r 1QA9_l 1GRN_r
1JPS_l	*Homo_sapiens*	67	0.25	94	0.25	118	0.50	1BVK_l 1KTZ_r
1MLC_l	*Gallus_gallus*	9	0.12	41	0.25	65	0.38	1E6E_l 1ATN_r 1KAC_l
1BGX_l	*Thermus_aquaticus*	1	0.00	1	0.00	1	0.00	
1BVK_l	*Gallus_gallus*	9	0.00	41	0.36	65	0.45	1BVN_r 1E6E_l 1BUH_l 1KAC_l 1E96_r
1I9R_l	*Homo_sapiens*	67	0.14	94	0.29	118	0.43	1DQJ_r 2VIS_r 1FQJ_l 2BTF_l 1AK4_r 1GRN_l
1BJ1_r	*Mus_musculus*	30	0.60	82	0.60	111	0.80	2VIS_r 1WQ1_l 1M10_l 1GRN_r
1IQD_r	*Homo_sapiens*	67	0.44	94	0.56	118	0.62	1E6E_l 1BVK_r 1KXQ_r 1ATN_l 1WQ1_l 1HE1_l 1HE8_l 1H1V_r 1I4D_l 1EER_l
2JEL_r	*Mus_musculus*	30	0.18	82	0.45	111	0.45	1DQJ_r 1IBR_r 2BTF_l 1GP2_r 1SBB_r
1NCA_r	*Mus_musculus*	30	0.12	82	0.25	111	0.62	1E6J_r 1AHW_l 1EER_r 1IB1_l 1GHQ_l
1QFW_r	*Mus_musculus*	30	0.14	82	0.86	111	0.86	1IQD_l 1FQJ_l 1I2M_r 1GP2_r 1A2K_l 1SBB_r
1FSK_r	*Mus_musculus*	30	0.25	82	0.75	111	0.75	1DQJ_r 1KXQ_r 1K5D_r 1HE1_l 1E96_r 1GRN_r
2QFW_r	*Mus_musculus*	30	0.00	82	0.33	111	1.00	1PPE_r 1KXP_l 1HE8_r
1K4C_r	*Mus_musculus*	30	0.00	82	0.33	111	0.33	1IBR_r
2HMI_l	*Mus_musculus*	30	0.14	82	0.57	111	1.00	1KXQ_l 1IBR_r 1WQ1_l 1I2M_r 1M10_l 1A2K_l 1FC2_l
1NSN_r	*Mus_musculus*	30	0.17	82	0.50	111	0.50	1IBR_r 1A2K_l 1GRN_r
1KXQ_r	*Sus_scrofa*	15	0.00	67	0.17	88	0.17	1GRN_r
1I9R_r	*Homo_sapiens*	67	0.25	94	0.25	118	0.75	2VIS_r 1BGX_r 1BUH_l
1BJ1_l	*Homo_sapiens*	67	0.40	94	0.80	118	1.00	1VFB_l 1WEJ_l 1AKJ_l 1I2M_r 1GP2_r
1IQD_l	*Homo_sapiens*	67	0.29	94	0.43	118	0.64	1MAH_r 1F34_r 1HIA_r 1QFW_r 1ATN_l 1KXP_r 1HE1_l 1IJK_l 2BTF_r
2JEL_l	*Escherichia_coli*	4	0.10	6	0.10	25	0.10	2JEL_l
1NCA_l	*Influenza_A_virus*	2	0.00	2	0.00	2	0.00	
1QFW_l	*Homo_sapiens*	67	0.73	94	0.82	118	0.82	1E6E_l 1EAW_r 1FAK_r 1DE4_r 1ATN_r 1HE1_l 2BTF_r 1M10_r 1E96_r

Table S4: **Analysis of species distribution among proteins of the Mintseris dataset.** Continued; see legend of Fig. S3.

**PDB**	**Species**	**100%**		**80%**		**60%**		**PDB - partners**
		#homo	ratio	#homo	ratio	#homo	ratio	
1FSK_l	*Betula_pendula*	1	0.00	1	0.00	2	0.00	
2QFW_l	*Homo_sapiens*	67	0.73	94	0.82	118	0.82	1EAW_r 1E6J_r 1FAK_r 1ATN_r 1WQ1_l 1HE1_l 2BTF_r 1M10_r 1E96_r
1K4C_l	*Streptomyces_lividans*	1	0.00	1	0.00	3	0.00	
2HMI_r	*HIV_1*	3	0.00	3	0.00	8	0.00	
1NSN_l	*Staphylococcus_aureus*	4	0.07	4	0.07	5	0.07	1FC2_r
1KXQ_l	*Camelus_dromedarius*	1	0.00	8	0.00	11	0.00	
1AKJ_r	*Homo_sapiens*	67	0.40	94	0.40	118	0.40	1HE1_l 1FQ1_r
1K5D_r	*Homo_sapiens*	67	0.33	94	0.67	118	0.83	1E6J_r 1FSK_r 1KXQ_l 1B6C_r 1KTZ_r
1ML0_r	*Murid_herpesvirus_4*	1	0.00	1	0.00	1	0.00	
1FAK_r	*Homo_sapiens*	67	0.40	94	0.40	118	0.60	1DQJ_r 1QFW_l 2QFW_l
1IBR_r	*Homo_sapiens*	67	0.25	94	0.38	118	0.88	1D6R_r 2JEL_r 1K4C_r 2HMI_l 1NSN_r 1IB1_r 1A2K_r
1N2C_r	*Azotobacter_vinelandii*	2	0.00	2	0.00	2	0.00	
1DE4_r	*Homo_sapiens*	67	0.20	94	0.20	118	0.20	1QFW_l
1ATN_r	*Oryctolagus_cuniculus*	9	0.14	59	0.14	79	0.57	1MLC_l 1QFW_l 2QFW_l 1B6C_r
1F51_r	*Bacillus_subtilis*	4	0.00	5	0.00	8	0.00	
1GCQ_r	*Homo_sapiens*	67	0.00	94	0.00	118	0.00	
1KXP_r	*Oryctolagus_cuniculus*	9	0.20	59	0.40	79	0.60	1IQD_l 1ML0_l 1KTZ_r
1WQ1_r	*Homo_sapiens*	67	0.38	94	0.50	118	0.50	1WQ1_l 1GCQ_l 1HE8_l 1M10_r
1KLU_r	*Homo_sapiens*	67	0.29	94	0.43	118	0.57	1ML0_l 1IBR_r 1FQJ_l 1FC2_r
1HE1_r	*Pseudomonas_aeruginosa*	1	0.00	1	0.00	1	0.00	
1B6C_r	*Homo_sapiens*	67	0.71	94	0.71	118	0.71	1K5D_r 1IBR_l 1ATN_r 1EER_r 1GRN_r
1FQJ_r	*Bos_taurus*	21	0.20	69	0.60	90	0.80	1HIA_r 1E6E_l 1HE8_l 1KTZ_l
1IJK_r	*Bothrops_jararaca*	1	0.00	1	0.00	1	0.00	
1HE8_r	*Homo_sapiens*	67	0.17	94	0.33	118	0.50	2QFW_r 1FQJ_l 1A2K_r
1FQ1_r	*Homo_sapiens*	67	0.43	94	0.43	118	0.43	1I9R_l 1AKJ_r 1IBR_l
1BUH_r	*Homo_sapiens*	67	0.00	94	0.00	118	0.17	1FC2_r
1I2M_r	*Homo_sapiens*	67	0.38	94	0.38	118	0.88	1AVX_r 1D6R_r 1BJ1_l 1QFW_r 2HMI_l 1FQ1_l 1A2K_r
2BTF_r	*Bos_taurus*	21	0.00	69	0.43	90	0.86	1IQD_l 1QFW_l 2QFW_l 1ML0_l 1KTZ_r 1AK4_r
1M10_r	*Homo_sapiens*	67	0.62	94	0.62	118	0.62	1QFW_l 2QFW_l 1ML0_l 1WQ1_r 1QA9_r
1H1V_r	*Oryctolagus_cuniculus*	9	0.00	59	0.00	79	0.40	1IQD_r 1ML0_l
1I4D_r	*Homo_sapiens*	67	0.50	94	0.50	118	0.62	1I4D_l 1ATN_l 1WQ1_l 1HE8_l 1E96_r
1GP2_r	*Rattus_norvegicus*	11	0.00	61	0.20	103	0.80	1AHW_r 1BJ1_l 2JEL_r 1QFW_r
1RLB_r	*Homo_sapiens*	67	0.43	94	0.57	118	0.57	1RLB_l 1B6C_l 1I4D_l 1KAC_l
1QA9_r	*Homo_sapiens*	67	0.67	94	0.67	118	0.83	1HE1_l 1M10_r 1I4D_l 1E96_r 1SBB_r
1KAC_r	*Human_adenovirus_12*	1	0.00	1	0.00	1	0.00	
1EER_r	*Homo_sapiens*	67	0.50	94	0.67	118	1.00	1E6E_l 2VIS_r 1NCA_r 1WQ1_l 1B6C_r 1BUH_l
1IB1_r	*Homo_sapiens*	67	0.40	94	0.40	118	0.60	1IBR_r 1BUH_l 1SBB_r
1A2K_r	*Rattus_norvegicus*	11	0.00	61	0.67	103	0.67	1A2K_l 1IBR_r 1HE8_r 1I2M_r
1KTZ_r	*Homo_sapiens*	67	0.50	94	0.62	118	0.75	1CGI_r 1HIA_r 1JPS_l 1K5D_r 1KXP_r 2BTF_r
1GHQ_r	*Homo_sapiens*	67	0.50	94	0.50	118	0.67	1EAW_r 1BGX_r 1BVK_r 1I4D_l
1AK4_r	*Homo_sapiens*	67	0.60	94	0.60	118	0.80	1I9R_l 1ATN_l 1IJK_l 1EER_l
1E96_r	*Homo_sapiens*	67	0.46	94	0.62	118	0.92	1VFB_l 1DQJ_l 2VIS_r 1BVK_l 1QFW_l 1FSK_r 2QFW_l 1I4D_r 1GP2_l 1QA9_r 1GHQ_r 1GRN_l
1FC2_r	*Staphylococcus_aureus*	4	0.12	4	0.12	5	0.12	1NSN_l
1GRN_r	*Homo_sapiens*	67	0.27	94	0.55	118	0.82	1VFB_l 1WEJ_l 2VIS_r 1AHW_l 1BJ1_r 1NSN_r 1KXQ_r 1B6C_r 1FQ1_l

Table S5: **Analysis of species distribution among proteins of the Mintseris dataset.** Continued; see legend of Fig. S3.

PDB	Species	100%		80%		60%		PDB - partners
		#homo	ratio	#homo	ratio	#homo	ratio	
1SBB_r	*Mus_musculus*	30	0.38	82	0.62	111	0.62	2JEL_r 1QFW_r 1B6C_l 1I4D_l 1IB1_r
1AKJ_l	*Homo_sapiens*	67	0.50	94	0.50	118	0.75	1BJ1_l 1NCA_r 1NSN_r 1KXP_l 1HE8_r 1EER_l
1K5D_l	*Schizosaccharomyces_pombe*	1	0.00	9	0.00	20	0.00	
1ML0_l	*Homo_sapiens*	67	0.67	94	0.75	118	0.75	1AHW_r 1KXP_r 1KLU_r 1IJK_l 1I2M_l 2BTF_r 1M10_r 1H1V_r 1EER_l
1FAK_l	*Homo_sapiens*	67	0.00	94	0.00	118	0.50	2JEL_r
1IBR_l	*Homo_sapiens*	67	0.43	94	0.71	118	0.71	1ACB_r 1E6J_r 1B6C_r 1FQ1_r 1E96_l
1N2C_l	*Azotobacter_vinelandii*	2	0.00	2	0.00	2	0.00	
1DE4_l	*Homo_sapiens*	67	0.20	94	0.20	118	0.20	1CGI_l
1ATN_l	*Bos_taurus*	21	0.20	69	0.60	90	0.80	1VFB_l 1IQD_l 1I4D_r 1AK4_r
1F51_l	*Bacillus_subtilis*	4	0.00	5	0.00	8	0.00	
1GCQ_l	*Mus_musculus*	30	0.25	82	0.38	111	0.75	1MAH_r 1ACB_r 1E6E_r 2QFW_l 1B6C_l 1FC2_l
1KXP_l	*Homo_sapiens*	67	0.17	94	0.50	118	0.67	1ACB_r 2QFW_r 1AKJ_l 1RLB_l
1WQ1_l	*Homo_sapiens*	67	0.50	94	0.62	118	0.75	1WQ1_r 1BJ1_r 2HMI_l 1I4D_r 1EER_r 1GHQ_l
1KLU_l	*Staphylococcus_aureus*	4	0.00	4	0.00	5	0.00	
1HE1_l	*Homo_sapiens*	67	0.53	94	0.88	118	0.94	1CGI_r 1ACB_r 1E6E_r 1VFB_r 2VIS_r 1BVK_r 1IQD_r 1QFW_l 1FSK_r 2QFW_l 1AKJ_r 1I4D_r 1GP2_l 1QA9_r 1KAC_l 1GHQ_l
1B6C_l	*Homo_sapiens*	67	0.17	94	0.33	118	0.33	1GCQ_l 1RLB_r
1FQJ_l	*Bos_taurus*	21	0.00	69	0.38	90	0.38	1KLU_r 1HE8_r 1EER_l
1IJK_l	*Homo_sapiens*	67	0.50	94	0.50	118	0.50	1IQD_l 1ML0_l 1AK4_r
1HE8_l	*Homo_sapiens*	67	0.38	94	0.62	118	0.92	1CGI_r 2PCC_l 1VFB_l 1E6J_r 1DQJ_l 1MLC_l 1IQD_r 1WQ1_r 1FQJ_r 1I4D_r 1AK4_r 1GRN_l
1FQ1_l	*Homo_sapiens*	67	0.60	94	0.60	118	0.60	1DE4_r 1I2M_r 1GRN_r
1BUH_l	*Homo_sapiens*	67	0.71	94	0.86	118	1.00	1MLC_r 1BVK_l 1I9R_r 1H1V_l 1EER_r 1IB1_r 1E96_l
1I2M_l	*Homo_sapiens*	67	0.14	94	0.14	118	0.29	1DQJ_l 1ML0_l
2BTF_l	*Bos_taurus*	21	0.00	69	0.14	90	0.14	1EER_l
1M10_l	*Homo_sapiens*	67	0.40	94	0.80	118	1.00	1WEJ_r 1JPS_r 1BJ1_r 2HMI_l 1KTZ_l
1H1V_l	*Homo_sapiens*	67	0.00	94	0.00	118	0.20	1BGX_r
1I4D_l	*Homo_sapiens*	67	0.45	94	0.73	118	0.91	1I4D_r 1ACB_r 1EZU_r 1MLC_r 1IQD_r 1NSN_r 1QA9_r 1KAC_l 1GHQ_r 1SBB_r
1GP2_l	*Bos_taurus*	21	0.00	69	0.50	90	0.75	2PCC_l 1HE1_l 1E96_r
1RLB_l	*Gallus_gallus*	9	0.00	41	0.00	65	0.33	1RLB_r
1QA9_l	*Homo_sapiens*	67	0.20	94	0.60	118	0.60	1E6E_l 1AHW_l 1MLC_r
1KAC_l	*Homo_sapiens*	67	0.60	94	0.60	118	1.00	1MLC_l 1BVK_l 1HE1_l 1I4D_l 1RLB_r
1EER_l	*Homo_sapiens*	67	1.00	94	1.00	118	1.00	1IQD_r 1AKJ_l 1ML0_l 1AK4_r
1IB1_l	*Ovis_aries*	7	0.00	65	0.00	85	0.00	
1A2K_l	*Canis_lupus*	9	0.20	64	0.20	90	0.20	1A2K_r
1KTZ_l	*Homo_sapiens*	67	0.43	94	0.57	118	0.86	2PCC_l 1DQJ_l 1QFW_l 1FQJ_r 1M10_l 1E96_l
1GHQ_l	*Homo_sapiens*	67	0.60	94	0.80	118	1.00	1BVN_r 1JPS_r 1NCA_r 1WQ1_l 1HE1_l
1AK4_l	*HIV_1*	3	0.20	3	0.20	8	0.60	1E6J_l 1JPS_r 1I9R_l
1E96_l	*Homo_sapiens*	67	0.60	94	0.60	118	0.60	1IBR_l 1BUH_l 1KTZ_l
1FC2_l	*Homo_sapiens*	67	0.00	94	0.00	118	0.25	2HMI_l
1GRN_l	*Homo_sapiens*	67	0.71	94	0.86	118	0.86	1E6E_l 1JPS_r 1I9R_l 1ATN_r 1HE8_l 1E96_r
1SBB_l	*Staphylococcus_aureus*	4	0.00	4	0.00	5	0.17	1KKL_l

Table S6: **Analysis of species distribution among proteins of the Mintseris dataset.** Continued; see legend of Fig. S3.

HEX parameterization	
**Parameter**	**Value**
RECEPTOR_RANGE_ANGLE	180
LIGAND_RANGE_ANGLE	180
TWIST_RANGE_ANGLE	360
DOCKING_R12_RANGE	40
DOCKING_R12_STEP	0.75
DOCKING_R12_SUBSTEPS	2
MAX_DOCKING_SOLUTIONS	3000
MAX_DOCKING_CLUSTERS	2000
DOCKING_CLUSTER_WINDOW	200
DOCKING_CLUSTER_THRESHOLD	3
DOCKING_CLUSTER_MODE	1
DOCKING_CORRELATION	0
DOCKING_MAIN_SCAN	18
DOCKING_MAIN_SEARCH	25

Table S7: **Parameters used for HEX CC-D computation.**

Evaluation of partner prediction based on experimental interfaces							
**NII score threshold = 0.4**							
**Subset Type**	**# proteins**	**AUC**	**Sen**	**Spe**	**Prec**	**F1**	**MCC**
Mintseris DB	168	0.84	65	86	3	5	11
Enzyme-Inhibitors & Others	124	0.84	64	85	4	7	12
Enzyme-Inhibitors	46	0.85	63	81	7	13	16
Antibody-Antigens	20	0.89	100	50	10	17	22
Antibody-bound Antigens	24	0.91	96	68	12	21	27
Others	78	0.84	72	82	5	9	15
Rigid	126	0.87	76	84	4	7	14
Medium	26	0.85	88	71	11	19	25
Difficult	16	0.77	69	67	12	21	18
Mono (both partners)	86	0.87	76	82	5	9	16
Multi (at least one partner)	82	0.81	71	79	4	8	13
**NII score threshold = 0.5**							
Mintseris DB	168	0.84	52	92	4	7	12
Enzyme-Inhibitors & Others	124	0.84	54	92	5	9	15
Enzyme-Inhibitors	46	0.85	59	88	10	17	20
Antibody-Antigens	20	0.89	95	66	13	23	28
Antibody-bound Antigens	24	0.91	79	80	15	25	28
Others	78	0.84	62	89	7	12	18
Rigid	126	0.87	59	91	5	9	15
Medium	26	0.85	73	81	13	22	25
Difficult	16	0.77	69	78	17	28	26
Mono (both partners)	86	0.87	66	89	7	12	19
Multi (at least one partner)	82	0.81	59	88	6	10	15
**NII score threshold = 0.6**							
Mintseris DB	168	0.84	43	96	6	10	14
Enzyme-Inhibitors & Others	124	0.84	47	95	8	13	17
Enzyme-Inhibitors	46	0.85	52	93	14	22	24
Antibody-Antigens	20	0.89	75	77	15	25	26
Antibody-bound Antigens	24	0.91	71	86	18	29	31
Others	78	0.84	49	94	9	15	19
Rigid	126	0.87	47	95	7	11	16
Medium	26	0.85	62	88	17	26	27
Difficult	16	0.77	62	82	19	29	27
Mono (both partners)	86	0.87	52	94	9	15	20
Multi (at least one partner)	82	0.81	45	93	7	13	16

Table S8: **Evaluation of partner identification prediction based on experimental interfaces.** The evaluation is realized at different NII score thresholds: 0.4, 0.5 and 0.6. Recall that the NII score threshold defines the set of predicted interacting complexes (that is, true and false positives, TP+FP). We use five standard measures of performance: sensitivity $Sen = TP/(TP + FN)$, specificity $Spe = TN/(TN + FP)$, precision or positive predictive value $Prec = TP/(TP + FP)$, balanced F-score $F1 = 2 \cdot \frac{Prec \cdot Sen}{Prec \cdot Sen}$ and Matthews correlation coefficient $MCC = (TP \cdot TN - FP \cdot FN)/K$ where $K = \sqrt{(TP + FP)(TP + FN)(TN + FP)(TN + FN)}$.

Evaluation of partner prediction based on predicted interfaces (JET+NIP)							
**NII score threshold = 0.15**							
**Subset Type**	**# proteins**	**AUC**	**Sen**	**Spe**	**Prec**	**F1**	**MCC**
Mintseris DB *	168	0.61	35	80	1	2	3
Enzyme-Inhibitors & Others *	124	0.66	43	77	2	3	4
Enzyme-Inhibitors	46	0.77	76	66	4	8	10
Antibody-Antigens	20	0.58	20	57	2	4	-10
Antibody-bound Antigens	24	0.56	50	65	6	10	7
Others *	78	0.61	37	79	2	4	5
Rigid *	126	0.60	37	74	1	2	2
Medium	26	0.68	58	71	7	13	12
Difficult	16	0.65	38	75	9	15	7
Mono (both partners)	86	0.63	47	74	2	4	5
Multi (at least one partner)	80	0.61	42	77	2	4	5
**NII score threshold = 0.20**							
Mintseris DB *	168	0.61	32	85	1	3	4
Enzyme-Inhibitors & Others *	124	0.66	39	83	2	4	5
Enzyme-Inhibitors	46	0.77	70	73	4	8	11
Antibody-Antigens	20	0.58	15	66	2	4	-9
Antibody-bound Antigens	24	0.56	46	71	6	11	7
Others *	78	0.61	30	84	3	5	5
Rigid *	126	0.60	30	81	1	2	3
Medium	26	0.68	58	76	9	15	15
Difficult	16	0.65	38	78	10	16	9
Mono (both partners)	86	0.63	38	80	2	4	5
Multi (at least one partner)	80	0.61	36	82	3	5	5
**NII score threshold = 0.25**							
Mintseris DB *	168	0.61	25	89	1	3	4
Enzyme-Inhibitors & Others *	124	0.66	34	87	2	4	6
Enzyme-Inhibitors	46	0.77	65	78	5	9	12
Antibody-Antigens	20	0.58	15	70	3	4	-7
Antibody-bound Antigens	24	0.56	38	74	6	10	5
Others *	78	0.61	25	88	3	5	4
Rigid *	126	0.60	29	85	2	3	4
Medium	26	0.68	58	80	10	18	18
Difficult	16	0.65	38	80	11	17	10
Mono (both partners)	86	0.63	36	85	3	5	6
Multi * (at least one partner)	80	0.61	32	86	3	5	6

Table S9: **Evaluation of partner identification prediction based on predicted interfaces (JET+NIP).** We report three analyses realized at three different NII score thresholds (0.15, 0.20 and 0.25). Thresholds are used to define the set of predicted interacting complexes (that is, true and false positives, TP+FP). We use the five standard measures of performance defined in the legend of SI Table. S8. For each analysis, performance is evaluated on different categories of the Minseris dataset: functional, difficulty and multimeric vs monomeric complexes. The * symbol refers to values computed on subsets that have been cleaned of the complex 1ML0 for which JET provided no interaction site.

Evaluation of the performance of binding sites predictions						
**NIP[0.4]+JET[0.6] - score threshold of 0.5**						
**Subset Type**	**# proteins**	**Sen**	**Spe**	**Prec**	**F1**	**MCC**
Mintseris DB *	166	30	83	25	31	13
Enzyme-Inhibitors & Others *	122	37	84	31	37	20
Enzyme-Inhibitors	46	45	80	41	42	26
Antibody-Antigens	20	8	80	5	9	-10
Antibody-bound Antigens	24	14	80	10	15	-3
Others *	76	32	86	25	33	17
Rigid *	124	30	80	24	30	11
Medium	26	39	89	34	39	26
Difficult	16	17	90	18	25	8
**NIP - score threshold of 0.5**						
Mintseris DB *	166	28	92	37	37	22
Enzyme-Inhibitors & Others *	122	24	92	35	35	19
Enzyme-Inhibitors	46	28	93	50	38	27
Antibody-Antigens	20	37	93	45	57	33
Antibody-bound Antigens	24	41	94	45	48	36
Others *	76	20	92	26	30	13
Rigid *	124	29	92	38	40	23
Medium	26	24	93	35	31	20
Difficult	16	22	94	27	26	16
**JET - score threshold of 0.5**						
Mintseris DB *	166	33	79	23	30	12
Enzyme-Inhibitors & Others *	122	40	80	29	35	18
Enzyme-Inhibitors	46	48	77	38	42	24
Antibody-Antigens	20	11	75	6	11	-10
Antibody-bound Antigens	24	17	75	9	14	-5
Others *	76	35	82	23	30	15
Rigid *	124	32	76	22	29	9
Medium	26	42	86	32	36	25
Difficult	16	21	87	17	22	7
**JET on the good patch - score threshold of 0.5**						
Mintseris DB *	141	37	85	36	34	23
Enzyme-Inhibitors & Others *	94	48	84	48	45	34
Enzyme-Inhibitors	42	52	80	53	45	35
Antibody-Antigens	8	14	86	19	14	1
Antibody-bound Antigens	13	21	85	21	20	7
Others *	52	45	88	44	41	33
Rigid *	84	44	82	41	39	27
Medium	24	43	91	47	42	35
Difficult	7	31	93	53	36	30

Table S10: **Evaluation of the performance of binding sites predictions.** Different types of predictive scores are considered: NIP[0.4]+JET[0.6], NIP, JET and JET on the patch displaying the highest coverage of the experimental interface. The score cut-off is 0.5, for all types of scores. Performance is evaluated on proteins for which JET predicts at least one cluster overlapping the experimental interface (second column). The * symbol refers to values computed on subsets that have been cleaned of the complex 1ML0 for which JET provided no interaction site. See also Fig. S77 for the ROC curves reporting the ability of NIP or JET to predict interface residues on the Mintseris dataset and its different subsets.

**Subset Type**	**Protein Type**	**# proteins**	**Sen**	**Spe**	**Prec**	**F1**	**MCC**
Mintseris DB *	Mono	110	37	82	33	37	19
	Multi	56	18	83	10	18	2
Enzyme-Inhibitors & Others *	Mono	94	40	83	35	40	23
	Multi	28	27	85	17	24	10
Enzyme-Inhibitors	Mono	42	46	79	43	43	26
	Multi	4	34	87	23	26	19
Antibody-Antigens	Mono	9	10	76	8	11	-13
	Multi	11	6	83	3	6	-7
Antibody-bound Antigens	Mono	7	24	79	25	24	5
	Multi	17	11	81	4	9	-6
Others *	Mono	52	35	86	29	38	20
	Multi	24	26	85	15	24	9
Rigid *	Mono	86	37	80	32	36	17
	Multi	38	14	81	6	13	-3
Medium	Mono	16	40	91	40	47	30
	Multi	10	37	86	25	29	20
Difficult	Mono	8	22	91	27	31	13
	Multi	8	13	89	9	16	2

Table S11: **Evaluation of the performance of NIP+JET binding site predictions on monomeric (Mono) and multimeric (Multi) receptors or ligands, using a score cut-off of 0.5.** To properly evaluate NIP+JET performance on a given protein we rely on the following quantities: the number of residues correctly predicted as interacting (true positives, TP), the number of residues correctly predicted as non-interacting (true negatives, TN), the number of non-interacting residues incorrectly predicted as interacting (false positives, FP) and the number of interacting residues incorrectly predicted as non-interacting (false negatives, FN). We use the five standard measures of performance defined in the legend of Table S8.
